# *N*-Benzylated 5-Hydroxybenzothiophene-2-carboxamides as Multi-Targeted Clk/Dyrk Inhibitors and Potential Anticancer Agents

**DOI:** 10.3390/cancers16112033

**Published:** 2024-05-27

**Authors:** Noha Mostafa, Po-Jen Chen, Sarah S. Darwish, Yu-Chieh Su, Ming-Hua Shiao, Gary A. Piazza, Ashraf H. Abadi, Matthias Engel, Mohammad Abdel-Halim

**Affiliations:** 1 Department of Pharmaceutical Chemistry, Faculty of Pharmacy and Biotechnology, German University in Cairo, Cairo 11835, Egypt; nohamostafaa1997@gmail.com (N.M.); s.sameh@gaf.edu.eg (S.S.D.); ashraf.abadi@guc.edu.eg (A.H.A.); 2 Department of Pharmaceutical Chemistry, School of Pharmacy, Newgiza University, Cairo 12256, Egypt; 3 Department of Medical Research, E-Da Hospital, I-Shou University, Kaohsiung 824410, Taiwan; ed113510@edah.org.tw; 4 Graduate Institute of Medicine, College of Medicine, I-Shou University, Kaohsiung 824410, Taiwan; hepatoma@gmail.com; 5 School of Life and Medical Sciences, University of Hertfordshire Hosted by Global Academic Foundation, New Administrative Capital, Cairo 11578, Egypt; 6 Division of Hematology-Oncology, Department of Internal Medicine, E-Da Hospital, I-Shou University, Kaohsiung 824410, Taiwan; 7 School of Medicine, College of Medicine, I-Shou University, Kaohsiung 824410, Taiwan; 8 Taiwan Instrument Research Institute, National Applied Research Laboratories, Hsinchu 300092, Taiwan; mhshiao@narlabs.org.tw; 9 Department of Drug Discovery and Development, Harrison College of Pharmacy, Auburn University, Auburn, AL 36832, USA; gap0034@auburn.edu; 10 Pharmaceutical and Medicinal Chemistry, Saarland University, Campus C2.3, D-66123 Saarbrücken, Germany

**Keywords:** Dyrk1A, Clk1, 5-hyrdoxybenzothiophene, multi-targeting, cell cycle analysis, apoptosis induction

## Abstract

**Simple Summary:**

The demand for multitarget-directed anticancer agents has been steadily increasing, as cancer remains a leading cause of death worldwide. Dyrk and Clk kinases play crucial roles in cancer cell division and survival. Our study presents novel Dyrk1/Dyrk1B/Clk1 inhibitors, developed by modifying our previous class of Clk1 inhibitors. These potent new inhibitors successfully halted the division of cancer cells without impacting normal cells. Additionally, we examined the effect of these compounds on the cell cycle to identify the specific phase affected. Finally, our compounds demonstrated the ability to activate pathways that induce cell death in cancer cells.

**Abstract:**

Numerous studies have reported that Dyrk1A, Dyrk1B, and Clk1 are overexpressed in multiple cancers, suggesting a role in malignant disease. Here, we introduce a novel class of group-selective kinase inhibitors targeting Dyrk1A, Dyrk1B, and Clk1. This was achieved by modifying our earlier selective Clk1 inhibitors, which were based on the 5-methoxybenzothiophene-2-carboxamide scaffold. By incorporating a 5-hydroxy group, we increased the potential for additional hydrogen bond interactions that broadened the inhibitory effect to include Dyrk1A and Dyrk1B kinases. Within this series, compounds **12** and **17** emerged as the most potent multi-kinase inhibitors against Dyrk1A, Dyrk1B, and Clk1. Furthermore, when assessed against the most closely related kinases also implicated in cancer, the frontrunner compounds revealed additional inhibitory activity against Haspin and Clk2. Compounds **12** and **17** displayed high potency across various cancer cell lines with minimal effect on non-tumor cells. By examining the effect of these inhibitors on cell cycle distribution, compound **17** retained cells in the G2/M phase and induced apoptosis. Compounds **12** and **17** could also increase levels of cleaved caspase-3 and Bax, while decreasing the expression of the antiapoptotic Bcl-2 protein. These findings support the further study and development of these compounds as novel anticancer therapeutics.

## 1. Introduction

Cancer is a major cause of death globally with reports of 19.3 million new cases and 10 million fatalities in 2020 [1,2]. Clearly, there is an urgent need for new target-direct anticancer agents.

Previously, the ‘single molecule–single target’ approach represented the most common strategy for the design of new pharmaceutical agents to avoid off-target side effects [3]. However, there is an increasing doubt that a single, selective, anticancer drug is an optimum strategy for treatment owing to the heterogenous, complex mutational landscape of most cancers [4]. Multi-targeted therapy is now regarded as a promising solution for advanced-stage disease. Multi-targeting can be attained using a combination of single targeted drugs [5] but often suffers from dose-limiting toxicities. Alternatively, multi-targeted therapy can be achieved using a single agent that can suppress the activity of different oncogenic targets, with the potential for an improved safety profile [3]. Kinases are recognized as vital regulators of cellular functions, and any slight imbalance in the protein phosphorylation process might result in cell proliferation disorder and cancer [6]. Given the homogeneity among many kinases, it is reasonable to consider small molecule kinase inhibitors as potential multi-targeted therapeutic options.

Dual-specificity tyrosine-regulated kinases (Dyrks) and Cdc2-like kinases (Clks) are classified as dual-specificity kinases that play an important role in cancer cell proliferation and survival by regulating protein phosphorylation [7,8,9]. Both Dyrks and Clks belong to the CMGC family of kinases [10]. There are five members within the mammalian Dyrk subfamily, in which they are categorized into two classes: class I, the most studied class, consists of Dyrk1A and Dyrk1B; while class II consists of Dyrk2, Dyrk3, and Dyrk4 [11].

In addition to the well-documented involvement of Dyrk1A in neurodegenerative diseases, research has shown that both Dyrk1A and its close relative Dyrk1B are overexpressed in a range of cancers, including glioblastoma, ovarian, lung, colorectal, and pancreatic cancers, suggesting a role in tumorigenesis [12,13,14] According to a study by Pozo et al., Dyrk1A inhibition increased EGFR degradation and inhibited glioblastoma growth via reducing EGFR-dependent tumor growth [12]. Furthermore, Dyrk1A plays an important role in cell survival by phosphorylating caspase 9 at Thr-125 to increase cancer cell survival by blocking apoptosis [15]. In addition, Dyrk1A maintains cancer cells in a quiescence state through the formation of the DREAM complex, which maintains cells in a noncycling G_0_ phase by stabilizing p27^Kip1^, a cyclin-dependent kinase (CDK) inhibitor, and finally through inducing the degradation of cyclin D isoforms [16]. Altogether, this leads to cell cycle exit, causing the chemoresistance of non-cycling cancer cells, as non-dividing cancer cells are relatively resistant to chemotherapeutic drugs [16,17]. Dyrk1A inhibitors such as harmine exhibit anticancer activity through apoptosis of human gastric cancer cells in vitro [18,19], in addition to leucettines [20], and EGCG which decrease prostate cancer xenograft growth [21,22]. Another potent Dyrk1A inhibitor, harmol, demonstrated anti-proliferative action and caused apoptosis in various cancer cell models [19,23,24]. Additionally, other Dyrk1A inhibitors such as pyrrolopyrimidine derivatives suppressed the growth of glioblastoma xenografts [25]. Furthermore, macrocyclic inhibitors, which were reported as potent Dyrk1A inhibitors, also exhibited antiproliferative effects and decreased colony formation in head and neck squamous cell carcinoma [26]. Similarly, some thiazolidines derivatives revealed antitumor activity against colorectal carcinoma cell lines [27].

Similar to the Dyrk1A homologue, Dyrk1B was shown to induce cancer cells into a dormant state, thus rendering the cells resistant to chemotherapy [16,17]. In addition, cancer cell survival is enhanced by the antiapoptotic activities of Dyrk1B [28,29,30]. In accordance, the overexpression of Dyrk1B was observed in many solid tumors such as colon and lung cancer, pancreatic ductal adenocarcinomas, and rhabdomyosarcomas [31]. The chemical inhibition of Dyrk1B using a selective and potent 6-azaindole-based compound abolished the degradation of certain cellular regulators in the cell cycle by inhibiting Dyrk1B phosphorylation activity and inducing apoptosis [29,32].

On the other hand, Clks, another member of CMGC family, plays an important role in regulating RNA splicing through the phosphorylation of members of the serine- and arginine-rich (SR) family of splicing factors, releasing these SR proteins from nuclear speckles into the nucleoplasm where they modulate the selection of splice sites during pre-mRNA splicing [33,34]. The most investigated Clk isoform to date is Clk1 [35]. In cancer, the overexpression of Clk1 results in the increased subcellular localization of SR proteins, increasing their concentration in the nucleus, which leads to aberrant alternative splicing producing pro-oncogenic variants and antiapoptotic proteins [36]. Many Clk1 inhibitors that have revealed anticancer efficacy have been reported, such as 5-methoxybenzothiophene-2-carboxamides, imides, and hydrazides, which had antiproliferative activity against bladder carcinoma cells [37,38,39,40]. Another inhibitor, 3,6-disubstutited-imidazo [1,2-a]pyridine, changed the subcellular rearrangement and repressed the phosphorylation of SR proteins in an ovarian cancer cell line [41]. These findings indicate that the combined suppression of Dyrk1A/1B and the simultaneous inhibition of Dyrk1A/1B/Clk1 kinases demonstrate favorable anticancer activity [42,43,44]. Noteworthy, it has been previously reported that Dyrk1A/Clk1 co-inhibition increased the efficacy of pre-mRNA splicing modulation, which could correct the abnormal splicing examined in cancer [45]. Moreover, a preliminary study has shown evidence that that the combination of a pan Dyrk/Clk inhibitor showed synergistic anticancer activity when combined with paclitaxel in vitro [46]. Collectively, the strategy of targeting Dyrk1A, Dyrk1B, and Clk1 simultaneously with a small molecule could offer significant benefits in tackling the complex nature of cancer.

**Figure 1:**
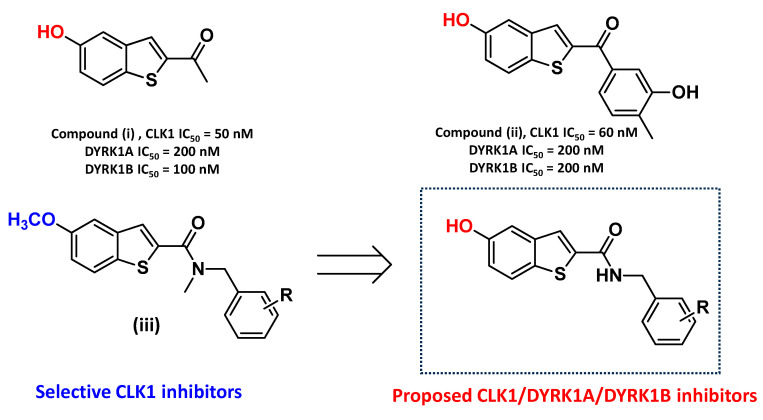
Design of a novel, group-selective scaffold by combining the hydroxybenzothiophene motif from our previous Dyrk1A/Clk1 inhibitors (**i**) and (**ii**) with the benzylamide moiety from our Clk1-selective scaffold (**iii**).

Based on the inhibitory profile of the previously reported 5-hydroxybenzothiophene ketones (compounds **i** and **ii**, Figure 1), we surmised that the exchange of the 5-methoxy in our previously presented 5-methoxybenzothiophene-2-carboxamides selective Clk1 inhibitors (**iii**) with a free hydroxyl possibly expands the inhibitory profile toward the related kinases, Dyrk1A and Dyrk1B, which should provide multi-targeted anticancer kinase inhibitors [38].

In the current study, we present a new class of group-selective (Dyrk1A/Dyrk1B/Clk1) inhibitors through the modification of the previously published selective Clk1 inhibitor 5-methoxybenzothiophene-2-carboxamide scaffold [38] by adopting the 5-hydroxy group to extend the inhibition towards multiple targeted kinases.

## 2. Results and Discussion

### 2.1. Compound Design

The design of the current compounds is based on the previously published 5-hydroxybenzothiophene ketones and 5-methoxybenzothiophene-2-carboxamides [38,45]. It has been previously shown that 5-hydroxybenzothiophene ketones such as compounds (**i**) and (**ii**) possess multiple targeting activities towards Clk1, Dyrk1A, and Dyrk1B (Figure 1) [38,45]. On the other hand, 5-methoxybenzothiophene-2-carboxamides (**iii**) elicited high selectivity for Clk1 vs. all other common off-targets (Figure 1) [38]. Therefore, in this study, we developed a new class of multi-target group-selective kinase inhibitors by adopting the 5-hydroxy group to the benzothiophene-2-carboxamides. The use of the smaller 5-hydroxy instead of the 5-methoxy is assumed to be better tolerated by the active site of many kinases. We also hypothesized that the hydroxyl group will be able to perform extra hydrogen bond interaction(s) with the homologous set of kinases of Clk1, Dyrk1A, and Dyrk1B. Our assumption was inspired by the similarity between the ATP binding pockets of the three kinases. Dyrk1A and Clk1 both are similar in terms of sequence and surface shape; in both kinases, the gatekeeper (gk) residue is phenylalanine, and also the three following amino acids are identical (gk+1; gk+3) or similar (gk+2: leucine in Clk1 and methionine in Dyrk1A) [47]. Furthermore, Dyrk1B’s ATP binding pocket is almost identical to Dyrk1A with just one amino acid substitution, Leu240 in Dyrk1A into Met192 in Dyrk1B, and this difference influenced neither hinge conformation nor ligand interactions [47,48,49,50]. Additionally, by varying the substitution pattern at the benzyl moiety linked to the carboxamide (Figure 1), we aimed to explore the inhibitory activity of this scaffold against the closely related family of kinases, to analyze the potential for group-selective inhibitors, where placing different substituents can tune selectivity toward single or pairs of kinases.

### 2.2. Chemistry

**Scheme 1:**
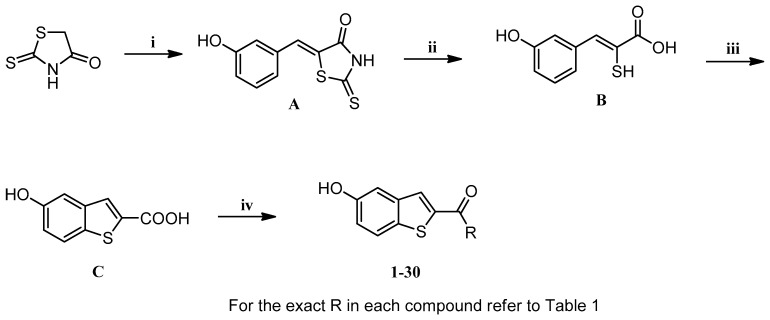
Reagents and conditions: (i) 1 equiv. of 3-hydroxybenzaldehyde, glacial CH_3_COOH, 0.15 equiv. anhydrous CH_3_COONa, toluene, 170 °C, reflux, 5 h; (ii) 15% NaOH, reflux, 1 h; (iii) 1.5 equiv. of iodine, THF, reflux, 2 days; (iv) 2 equiv. of EDC, 2 equiv. of DMAP, 1.2 equiv. of the corresponding amine, DMF, room temperature, overnight.

All planned final compounds were obtained through an amide coupling reaction between different benzyl amines and 5-hydroxy-benzo[b]thiophene-2-carboxylic acid (compound **C**, Scheme 1). The synthesis of the carboxylic acid (compound **C**) was successfully achieved through a trio of reactions outlined in Scheme 1. Initially, a reaction involving aldol condensation was conducted between rhodanine and 3-hydroxybenzaldehyde, utilizing glacial acetic acid and anhydrous sodium acetate, resulting in the formation of an α, β-unsaturated aldehyde (compound **A**). Subsequently, compound **A** underwent alkaline hydrolysis, employing sodium hydroxide, transforming into β-substituted-α-mercaptoacrylic acid (compound **B**). The third reaction involved the cyclization of compound **B** in the presence of iodine, leading to the formation of 5-hydroxy-benzo[b]thiophene-2-carboxylic acid (**C**). A comprehensive set of amide derivatives (**1**–**30**) was synthesized by reacting compound **C** with a variety of amines, employing EDC as the coupling agent and DMAP as the base, all within a DMF solvent environment (Scheme 1).

### 2.3. Biological Evaluation

The inhibitory activity of the synthesized compounds was evaluated against recombinant Dyrk1A, Dyrk1B, and Clk1 enzymes at a screening dose of 1 µM, which was comparable to the [ATP] = K_m_ value for each kinase. For compounds that showed more than 60%, the IC_50_ value was determined (Table 1).

#### 2.3.1. Structure–Activity Relationships (SARs) for Dyrk1A, Dyrk1B, and Clk1 Inhibition

Primarily, we tested the effect of adopting a free hydroxyl group instead of the 5-methoxy at the benzo[b]thiophene-2-carboxylic acid benzylamide scaffold, yielding probe compound **1**. Compound **1** exhibited modest inhibitory activity with 46% inhibition at 1 µM against Dyrk1A, and a slightly higher inhibitory activity against Dyrk1B and Clk1 with IC_50_ values of 833 nM and 740 nM, respectively. The inhibitory profile of compound **1** confirmed our assumption that uncovering the OH group at position 5 will lead to the loss of the scaffold selectivity towards Clk1.

Evaluation of electron-withdrawing substituents (EWG). To explore the structure–kinase selectivity relationship and improve the potency of compound **1**, different substituents at variable positions of the phenyl ring were tested. First, mono-substitution using EWGs was investigated at different positions. The *m*-chloro derivative (compound **3**, IC_50_: Dyrk1A = 751 nM, Dyrk1B = 530 nM, Clk1 = 257 nM) revealed an increase in potency against all kinases, compared to compound **1** with a slight preference towards Clk1. However, the *o*-chloro derivative kept the inhibitory activity against Dyrk1A (compound **2**, IC_50_ = 700 nM) and Clk1 (IC_50_ = 769 nM) but reduced the inhibitory activity against Dyrk1B. Finally, the *p*-chloro derivative (compound **4**) deteriorated inhibitory activity against all three kinases (Table 1), indicating a possible steric clash with the pocket. The same preference for the *meta* position was reproduced with the more electronegative fluorine, where the 3-fluorobenzyl derivative (compound **6**, IC_50_: Dyrk1A = 666 nM, Dyrk1B = 406 nM, Clk1 = 410 nM) exhibited improved activity against all three kinases, while the 4-fluorobenzyl derivative (compound **7**) revealed the least inhibitory activity compared to the *ortho* and *meta* congeners (Table 1).

Evaluation of electron-donating substituents (EDGs). To explore the effect of monosubstitution with EDGs, a methyl substituent was probed at the three positions (*o*, *m*, and *p*). The *o*-methyl analogue (compound **8**) showed a better inhibitory profile than the respective *m*- and *p*-congeners, with a slightly decreased inhibitory activity against Dyrk1B (% Inh. = 48%), while it maintained potency against Dyrk1A and Clk1 (IC_50_ = 853 nM and 594 nM, respectively). As for the methoxy substituent, the *ortho-* and *meta*-substituted analogues (**11** and **12,** respectively) revealed enhanced inhibitory activity, with compound **12** showing the most potent inhibition among all the tried substituents (IC_50_: Dyrk1A = 536 nM, Dyrk1B = 238 nM, Clk1 = 153 nM), keeping its preference in inhibition towards Clk1. Similar to all tested mono substituents, the *p*-methoxy showed a significant reduction in activity (Table 1).

Effect of multiple substitutions. Next, disubstitution patterns were evaluated using combinations of the substituents described above. Initially, the 3,5-dichloro analogue (compound **14**) was investigated, which resulted in decreased inhibitory activity against Dyrk1A and Dyrk1B but selective potency against Clk1 (IC_50_ = 744 nM). Consequently, difluoro derivatives were synthesized and evaluated (compounds **15**–**17**). The 3,5-difluorobenzyl analogue (compound **17**, IC_50_: Dyrk1A = 495 nM, Dyrk1B = 242 nM, Clk1 = 168 nM) was found to be one of the most potent derivatives of all synthesized compounds with a 4-fold improvement in inhibitory activity against Dyrk1B and Clk1 kinases compared to compound **1**. These results indicate that the effect of combining two fluorine atoms at the favored *meta* position (as shown previously) is at least additive in boosting the inhibitory activity. The 2,3-difluoro (compound **15**) did not boost the activity when compared to the monosubstituted fluorine analogues, and as expected, the 2,4-difluoro analogue (compound **16**) exhibited a general decrease in inhibitory activity against the three kinases (Table 1), confirming the deleterious effect of the *p*-fluoro on inhibitory activity. However, the replacement of the 3,5-difluorobenzyl analogue with a 3,5-trifluoromethyl (compound **18**) resulted in a sharp drop in activity against all three kinases (Table 1). The further testing of disubstitution patterns containing EDGs followed. An initial 3,5-dimethyl analogue (compound **19**) resulted in decreased inhibitory activity against the three kinases compared to compound **1** and compound **9** (the 3-methylbenyzl analogue). Testing the congeners containing dimethoxy substitution at variable positions **20**–**23** almost abolished the inhibitory activity against Dyrk1A, Dyrk1B, and Clk1 kinases (Table 1). This was not expected for the 3,5-dimethoxybenzyl (**23**) since the 3-methoxybenzyl derivative (**12**) was among the best monosubstituted compounds. These findings lead us to conclude that disubstitution by EDGs results in deleterious inhibitory activity against all three kinases.

Effect of rigidified analogues. The rigidification of the lead compound **1** with an ethylene bridge in compound **24** reduced inhibition against all three kinases. However, rigidifying the *m*-chlorobenzyl analogue compound **3** with a methylene spacer, yielding the isoindoline analogue compound **25,** conserved the inhibitory activity against Clk1 (IC_50_ = 608 nM), resulting in a drop in activity against Dyrk1A and Dyrk1B.

Replacing the phenyl ring with other heteroaryl moieties. To evaluate the importance of the phenyl ring in compound **1**, we replaced this moiety with a variety of heteroaryl groups. Initially, the replacement of the phenyl ring by a 3-pyridinyl moiety resulted in decreased activity against all three kinases. Conversely, when we assessed the impact of substituting the phenyl ring with a 2-thienyl or a 2-furanyl ring, as observed in compounds **27** and **28,** respectively, both replacements exhibited comparable inhibitory effects across all three kinases (Table 1). This indicates that the phenyl ring can be replaced with other heteroaryl structures while maintaining a similar level of potency.

Insertion of a spacer between -NH and aromatic moieties. To examine the effect of extending the spacer between the amino group and the aryl moieties, a two-carbon spacer was employed with phenyl and indolyl rings, yielding compounds **29**–**30**. Both compounds exhibited decreased inhibitory activity against Dyrk1A and Dyrk1B kinases (Table 1), while selectively inhibiting Clk1 (IC_50_ = 753 nM and 350 nM, respectively).

Since our goal was to develop multi-targeting inhibitors, five compounds (**3**, **6**, **11**, **12,** and **17**) were selected for further evaluation based on their superior potency against all three kinases, showing more than 60% inhibition at 1 µM.

#### 2.3.2. Screening against Relevant Kinases That Are Overexpressed in Several Types of Cancer

As mentioned above, our major objective was to extend the activity of the previously mentioned methoxybenzothiophene-2-carboxamide class towards Dyrk1A/Dyrk1B along with Clk1. Five compounds (**3**, **6**, **11**, **12,** and **17**) were selected based on their superior potency against all three kinases and were further evaluated for their inhibitory activity against the three relevant kinases that were frequently reported as being overexpressed in several types of cancer. Clk2 and Clk3 phosphorylate SR proteins involved in mRNA splicing [51,52]. Several studies reported the overexpression of Clk2 in breast tumors [51], colorectal cancer [53], and glioblastoma [54]. Compounds **12** and **17** exhibited inhibitory activity against Clk2 with IC_50_ values of 617 nM and 708 nM, as shown in Table 2. Notably, the overexpression of Clk3 in colorectal cancer [55] and hepatocellular carcinoma [56] was previously highlighted. However, none of the tested compounds revealed any significant inhibitory activity against Clk3. Another serine/threonine kinase known as Haspin (Haploid Germ Cell-Specific Nuclear Protein Kinase) is essential to normal mitosis progression and the mitotic phosphorylation of histone H3 at threonine 3 in mammalian cells [57,58,59]. Importantly, several studies have demonstrated that the expression of Haspin is significant in several cancer cells in addition to normal proliferating somatic cells [60]. Specifically, the overexpression of Haspin in breast [61], pancreas [62], skin [63,64], lung [63,64], bone [63,64], prostate [62], and bladder [65] was reported. By interfering with normal mitotic progression, Haspin knockdown or small molecule inhibition could stop the proliferation of cancer cells and trigger apoptosis [60]. Remarkably, 3 out of the 5 tested compounds revealed significant inhibitory activity towards Haspin as shown in Table 2, namely, compounds **6** (IC_50_ = 207), **12** (IC_50_ = 167 nM), and **17** (IC_50_ = 256 nM).

#### 2.3.3. Inhibition of Tumor Cell Viability in a Panel of Human Cancer Cell Lines

As previously mentioned, Dyrk1A, Dyrk1B, Clk1, Clk2, and Haspin play a significant role in the progression of different types of cancer. Dyrk1A was proposed to increase cell survival in cancer cells by blocking apoptosis in addition to causing cell cycle exit through the formation of the DREAM complex as well as degrading cyclin D. Similarly, Dyrk1B phosphorylates several cell cycle regulators (e.g., cyclin D) leading to a quiescent state. Moreover, Dyrk1B inhibition is correlated with DNA damage, apoptosis, and sensitivity to chemotherapeutic drugs [66]. Clk overexpression increases SR proteins in nucleus, leading to aberrant alternative splicing and producing pro-oncogenic variants and antiapoptotic proteins. Haspin is also essential in the normal mitotic progression; therefore, its inhibition could stop cancer cells’ proliferation. To this end, the growth-inhibitory activity of the most potent multi-kinase inhibitors (compounds **3**, **6**, **11**, **12,** and **17**) was evaluated against a panel of human cancer cell lines at a concentration of 20 µM. The six cell lines were selected based on the high expression level of the targeted kinases (Supporting Information Table S1), as published in the Protein Atlas database (https://www.proteinatlas.org/; accessed on 1 February 2023). Based on our findings (Table 3), the most sensitive cell lines were HCT-116, T24, and Hela cells, in agreement with the high expression levels of the targeted kinases displayed by these cells compared to the rest of the tested cell lines. Out of the 5 tested compounds, compounds **12** (IC_50_ = 6.4 µM (HCT-116), 6 µM (T24), 16.5 (U87)) and **17** (IC_50_ = 8.1 µM (HCT-116), 14.9 µM (T24), 18.1 (Hela)) displayed the most potent growth inhibition against the highest number of cell lines. Interestingly, compounds **12** and **17** (Table 3) showed potent and extended inhibitory activity against DyrkA/1B, Clk1/2, and Haspin. These results suggest that the multi-targeting of different kinases may be an efficient strategy for enhancing growth inhibition in cancer cells.

#### 2.3.4. Evaluation of Safety against Normal Cell Lines

We next assessed the effect of the most potent multi-targeting compounds, **12** and **17**, in normal human keratinocytes (HaCaT) and rat intestine epithelial cells (IEC-6) (Table 4). Although both compounds showed a potent inhibitory effect on the cell viability of cancer lines shown in Table 3, they exhibited a much weaker effect on cell viability against normal cells (Table 4), suggesting that both compounds have cancer cell selectivity and merit further study.

#### 2.3.5. Effect of Compounds **12** and **17** on Cell Cycle in T24 Cells

**Figure 2:**
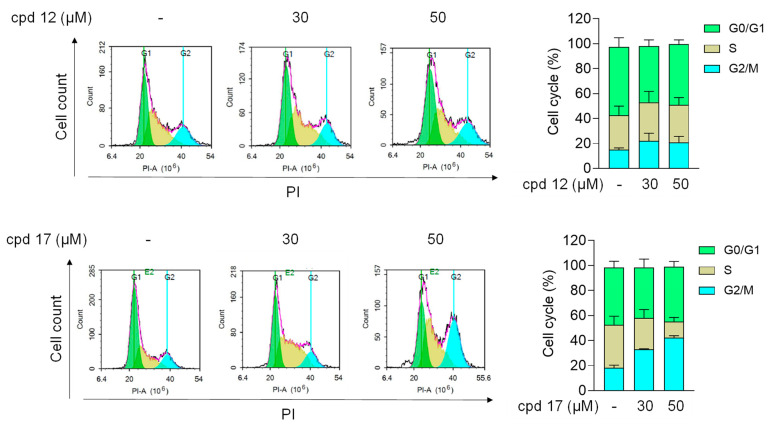
Analysis of cell cycle progression upon treatment with compounds **12** and **17**. T24 cells were treated with DMSO (0.1%), **12** (30–50 µM), or **17** (30–50 µM) for 24 h. Cell cycle was examined by propidium iodide (PI) staining and flow cytometry. All data are expressed as the mean ± S.E.M. (n = 3).

Both compounds **12** and **17** exhibit inhibitory effects on multiple kinases that have been documented to modulate cell cycle progression [17,67,68]. In the T24 cells, compound **17** was found to retain the cells in the G2/M phase during cell cycle progression, preventing the cells from entering the mitotic phase (Figure 2). Considering the multi-target profile of compound **17**, this cell cycle arrest might be partially attributable to inhibition of Haspin, which was found to be the most prominent target. It has been previously reported that the inhibition of Haspin-mediated histone H3 threonine 3 (H3T3) phosphorylation results in G2/M arrest and defects in centromeric localization [68]. Surprisingly, the inhibitory effects of compound **12** on the cell cycle of T24 cells were much weaker than those of compound **17**.

#### 2.3.6. Compounds **12** and **17** Lead to Apoptotic Cell Death in T24 Cells

**Figure 3:**
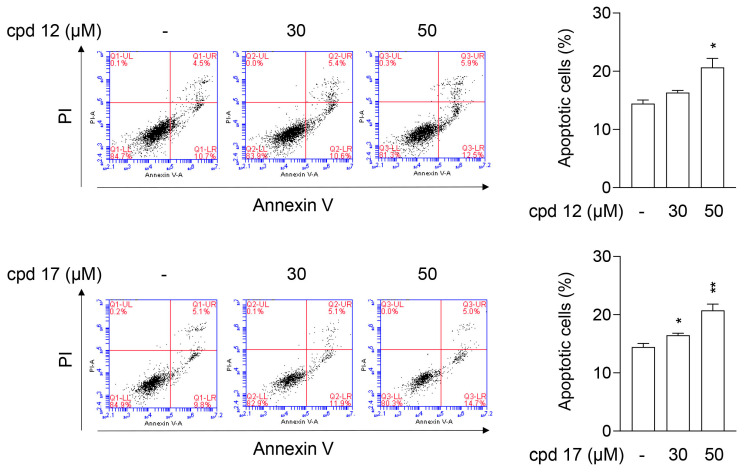
Annexin V and PI staining in T24 cells to investigate if compounds **12** and **17** could induce apoptotic death. T24 cells were treated with DMSO (0.1%), **12** (30–50 µM), or **17** (30–50 µM) for 24 h and then stained with Annexin V-FITC antibody and PI. The Annexin V-positive apoptotic cells were monitored using flow cytometry. All data are expressed as the mean ± S.E.M. (n = 3). * *p* < 0.05, ** *p* < 0.01 compared with the DMSO group.

Previous studies suggest that G2/M arrest leads to apoptosis [69,70,71]. Therefore, we determined if compounds **12** and **17** could induce apoptotic cell death in T24 cells, as measured by Annexin V and PI staining. Compound **17** dose-dependently increased the apoptotic cell population in T24 cells at 30 and 50 μM and compound **12** at 50 µM (Figure 3).

#### 2.3.7. Compounds **12** and **17** Trigger Proapoptotic Pathways in T24 Cells

**Figure 4:**
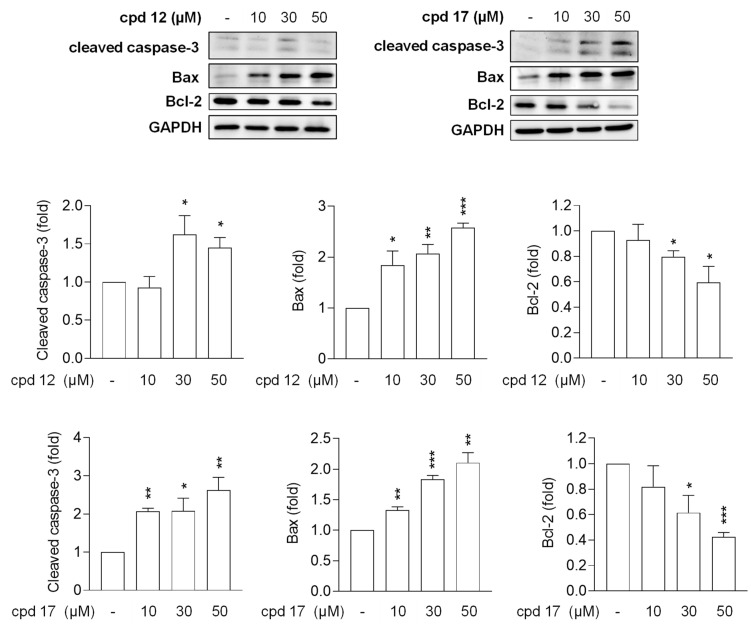
Examining the effect of **12** and **17** on proapoptotic and antiapoptotic pathways. T24 cells were treated with DMSO (0.1%), **12** (30–50 µM), or **17** (30–50 µM) for 24 h. Expression levels of cleaved caspase-3, Bax, Bcl-2, and GAPDH were analyzed by Western blot by corresponding antibodies. The quantitation of the cleaved caspase-3/GAPDH, Bax/GAPDH, and Bcl-2/GAPDH ratio are shown. All data are expressed as the mean ± S.E.M. (n = 3). * *p* < 0.05, ** *p* < 0.01, *** *p* < 0.001 compared with the DMSO group.

In addition, proapoptotic and antiapoptotic pathways were assayed to further determine the basis for the growth-inhibitory activity of compounds **12** and **17** using T24 cells. Both compounds dose-dependently (10–50 μM) increased the proapoptotic levels of cleaved caspase-3 and Bax. By contrast, both compounds decreased the expression of the antiapoptotic protein, Bcl-2 (Figure 4)

These results are consistent with previous literature reports that the gene knockdown of Dyrk1B causes the activation of caspase 3 in myoblasts [72]. In addition, 2,4-bisheterocyclic substituted thiophene derivatives showing potent Dyrk1B inhibitory activity increased caspase 3 activity [44]. Moreover, treatment using the Clk inhibitor, TG003, caused an elevation of activated caspase 3 levels in prostate cancer cells (PC3 and DU145) [73]. In addition, Haspin inhibition by CHR6494 caused an induction of the apoptotic protein levels of cleaved caspase 3 and Bax [74].

In accordance with the observed cellular growth inhibition, compounds **12** and **17** showed a dose-dependent decrease in the antiapoptotic marker Bcl-2 by approximately 0.5-fold at 50 μM. This can be explained by the previously reported effect of Dyrk1A and Clk1 on the increased expression of antiapoptotic proteins, Bcl-XL and Mcl-1, which are members of Bcl2 family [43,75].

## 3. Conclusions

A multi-targeting approach using a single agent is regarded as an attractive solution for the treatment of cancers having a complex mutational landscape. One suggested multi-targeting strategy involves the inhibition of Dyrk1A, Dyrk1B, and Clk1 kinases. These kinases play pivotal roles in various cellular processes that contribute to cancer development and progression. In this study, 5-hydroxybenzothiophene derivatives were designed and synthesized by modifying our previously published selective Clk1 inhibitor 5-methoxybenzothiophene-2-carboxamide scaffold. By replacing the 5-methoxy group with a 5-hydroxy, we were able to extend the inhibition towards multiple targeted kinases. Our SAR study showed that compounds **12** and **17** revealed the most potent inhibition against the three kinases (Dyrk1A/1B and Clk1). The most potent analogues were further tested against three highly homologous kinases, Clk2, Clk3, and Haspin, which are equally involved in several types of cancers. Of note, compounds **12** and **17** showed potent inhibition against Clk2 and Haspin. The testing of our most potent analogues was carried out against a panel of cancer cell lines known to express these kinases to determine growth-inhibitory activity. Noteworthy, compounds **12** and **17** displayed remarkable growth inhibition against the highest number of cell lines (HCT-116, T24, U8). Consequently, cell cycle analysis data showed that compound **17** could retain cells in the G2/M phase, leading to apoptosis in T24 cells. Additionally, compounds **12** and **17** could dose-dependently increase the proapoptotic levels of cleaved caspase-3 and Bax in T24 cells, while decreasing the expression of antiapoptotic Bcl-2. Our Dyrk1/Clk1/Haspin inhibitors showed very low toxicity against proliferating non-tumor cells, in contrast to many chemotherapeutic agents. These results show that several kinases can be inhibited simultaneously with selectivity for tumor cells; thus, it can be expected that adverse side effects are not strongly increased when co-administering with other chemotherapeutic drugs, e.g., taxanes. On the other hand, it has been shown that the simultaneous inhibition of multiple cancer-relevant targets increases the efficiency of and might delay resistance development in anticancer therapy. The partial inhibition of multiple kinases may resemble the situation created by low-dose drug combinations, which are now gaining more attention, as they demonstrate high efficacy with minimal adverse effects [76,77]. Altogether, our findings suggest that compounds **12** and **17** represent promising candidates for further evaluation as novel anticancer multi-targeting agents.

## 4. Experimental

### 4.1. Chemistry

All solvents and reagents were sourced from commercial suppliers and used without any further purification. Melting points were measured using a Stuart SMP3 melting point apparatus. The final compounds achieved a minimum purity of 95%, confirmed through high-performance liquid chromatography (HPLC) coupled with mass spectrometry. Mass spectra (HPLC-ESI-MS) were acquired using a TSQ Quantum instrument from Thermo Electron Corporation, equipped with a triple quadrupole mass detector and an electrospray ionization (ESI) source. Sample injections were performed using an autosampler (Surveyor, Thermo Finnigan) with an injection volume set to 10 μL. Mass spectrometric detection employed a source collision-induced dissociation (CID) voltage of 10 V, a spray voltage of 4.2 kV, nitrogen sheath gas at a pressure of 4.0 × 10^5^ Pa, a capillary temperature of 400 °C, a capillary voltage of 35 V, and an auxiliary gas pressure of 1.0 × 10^5^ Pa. The chromatographic separation utilized a reversed-phase C18 NUCLEODUR column (100-3, 125 mm × 3 mm) from Macherey & Nagel. The mobile phase consisted of two solvents: solvent A (water with 0.1% trifluoroacetic acid, TFA) and solvent B (acetonitrile with 0.1% TFA). The HPLC method was conducted with a flow rate of 400 μL/min, starting with 5% solvent B, linearly increasing to 100% over a span of 7 min, maintaining 100% for 2 min, then returning to 5% within 2 min, and held at 5% for another 2 min. Nuclear magnetic resonance (NMR) spectroscopy was performed on a Bruker DRX 400 spectrometer to obtain ^1^H and ^13^C NMR spectra. Chemical shifts were calibrated against the residual signals of the protonated solvent.

#### 4.1.1. General Synthetic Procedures and Experimental Details

##### Procedure for Synthesis of Compound **1**–**30** (Scheme 1)

A quantity of 40 mmol of 3-hydroxybenzaldehyde was added to 40 mmol of 2-thioxo-4-thiazolidinone in a round bottom flask containing 1 mL of glacial acetic acid, 0.5 g of anhydrous sodium acetate, and 70 mL toluene. The flask was refluxed for 5 h. A yellow precipitate was formed and then was vacuum filtered until complete dryness to yield compound **A**. A solution of 15% of NaOH was prepared, and 40 mL was added to a 250 mL round conical flask containing 44 mmol of compound **A**. The reaction flask then was refluxed for 1 h and stirred at 250 rpm. TLC was conducted to monitor the reaction progression. The reaction flask was placed in an ice bath. Then, 40 mL of 10% HCl was added to the reaction flask in the ice bath, where precipitation occurs. Extraction of the product was performed using ethyl acetate. The organic layer was collected and filtered over anhydrous MgSO_4_ in a 250 mL conical flask. The flask content was evaporated under reduced pressure until complete dryness to yield compound **B**. After that, 15 g of iodine was added to the reaction flask containing 40 mmol of compound **B** and 120 mL of tetrahydrofuran. The reaction flask was then refluxed for 2 days and stirred at 250 rpm. After 2 days, the reaction flask was removed, and consequently, 10% potassium iodide was prepared and 100 mL was added to the reaction flask, and the flask was left to stir. Around 90 g of sodium thiosulfate were added portionwise to the reaction flask and stirred simultaneously until two layers formed. Sodium thiosulfate was added until decolorization of the aqueous layer occurred. Extraction of the two layers was performed using 200 mL of diethyl ether. The organic phase was collected in a 250 mL conical flask. Then, extraction of the organic layer, from the previous step, was conducted using saturated sodium bicarbonate solution. The aqueous phase was collected in a 1000 mL beaker and placed in an ice bath. In a fume hood, 30–40 mL of conc. HCl was added dropwise to the 1000 mL beaker contents until no further effervescence occurs and precipitation of the product takes place. Finally, 150 mL of diethyl ether was used to extract the beaker contents. The organic layer was filtered over anhydrous MgSO_4_ in a weighed 250 mL conical flask and was evaporated under reduced pressure until complete dryness.

For purification, the least amount of dichloromethane (5 mL) was added along with 3–4 mL of hexane, and then the organic layer was evaporated in vacuo until completely dry to yield compound **C**.

5-Hydroxybenzothiophene carboxylic acid (1 mmol) was added along with 2 eq. of DMAP and EDC in 5 mL DMF. Then, 1.2 equivalent of the amine was added to the reaction mixture. The reaction mixture was left to stir overnight at room temperature. The product was then extracted using dichloromethane and water. The organic layer was then filtered over anhydrous MgSO_4_ in a 200 mL conical flask. Finally, the flask content was evaporated under vacuum until dry, and the products were further purified using silica gel column chromatography.

##### *N*-Benzyl-5-hydroxybenzo[b]thiophene-2-carboxamide (**1**)

The compound was synthesized according to Scheme 1 using benzylamine; yield: 74%. The product was purified by CC (DCM/MeOH 100:1); ^1^H NMR (400 MHz, DMSO-*d_6_*) δ 9.55 (s, 1H), 9.20 (s, 1H), 7.95 (s, 1H), 7.77 (d, *J* = 8.2 Hz, 1H), 7.34 (s, 3H), 7.27 (s, 1H), 7.23 (d, *J* = 12.3 Hz, 2H), 6.97 (d, *J* = 8.2 Hz, 1H), 4.48 (s, 2H). ^13^C NMR (101 MHz, DMSO-*d_6_*) δ 162.05, 155.64, 140.91, 139.73, 131.66, 128.76, 127.75, 127.42, 127.30, 124.81, 123.87, 117.37, 109.51, 43.10. MS (ESI): *m*/*z* = 284 (M+H)^+^.

##### *N*-(2-Chlorobenzyl)-5-hydroxybenzo[b]thiophene-2-carboxamide (**2**)

The compound was synthesized according to Scheme 1 using 2-chlorobenzylamine; yield: 45.31%. The product was purified by CC (DCM/MeOH 100:1); mp 140.2–142 °C; ^1^H NMR (400 MHz, DMSO-*d_6_*) δ 9.57 (s, 1H), 9.21 (s, 1H), 8.00 (s, 1H), 7.95 (s, 1H), 7.79 (d, *J* = 8.6 Hz, 1H), 7.46 (d, *J* = 7.2 Hz, 1H), 7.40 (d, *J* = 6.8 Hz, 1H), 7.33 (d, *J* = 6.3 Hz, 1H), 7.25 (d, *J* = 10.8 Hz, 1H), 6.98 (d, *J* = 8.7 Hz, 1H), 4.55 (d, *J* = 3.8 Hz, 2H). ^13^C NMR (101 MHz, DMSO-*d_6_*) δ 162.26, 155.68, 140.90, 140.50, 136.48, 132.47, 131.71, 129.61, 129.32, 129.17, 127.67, 125.13, 123.91, 117.48, 109.55, 41.04. MS (ESI): *m*/*z* = 318 (M+H)^+^.

##### *N*-(3-Chlorobenzyl)-5-hydroxybenzo[b]thiophene-2-carboxamide (**3**)

The compound was synthesized according to Scheme 1 using 3-chlorobenzylamine; yield: 58.98%. The product was purified by CC (DCM/MeOH 100:1); mp 211.2–212.3 °C; ^1^H NMR (400 MHz, DMSO-*d_6_*) δ 9.59 (s, 1H), 9.25 (s, 1H), 7.95 (s, 1H), 7.78 (d, *J* = 8.5 Hz, 1H), 7.37 (d, *J* = 8.9 Hz, 2H), 7.32 (d, *J* = 7.4 Hz, 2H), 7.23 (s, 1H), 6.98 (d, *J* = 8.7 Hz, 1H), 4.47 (d, *J* = 4.8 Hz, 2H). ^13^C NMR (101 MHz, DMSO-*d_6_*) δ 162.17, 155.66, 142.33, 140.88, 140.56, 133.42, 131.69, 130.70, 127.56, 127.29, 126.47, 125.02, 123.91, 117.48, 109.55, 42.60. MS (ESI): *m*/*z* = 318 (M+H)^+^.

##### *N*-(4-Chlorobenzyl)-5-hydroxybenzo[b]thiophene-2-carboxamide (**4**)

The compound was synthesized according to Scheme 1 using 4-chlorobenzylamine; yield: 37.70%. The product was purified by CC (DCM/MeOH 100:1); mp 182–183.7 °C; ^1^H NMR (400 MHz, DMSO-*d_6_*) δ 9.56 (s, 1H), 9.23 (s, 1H), 7.95 (s, 1H), 7.78 (d, *J* = 8.5 Hz, 1H), 7.38 (dd, *J* = 18.1, 7.4 Hz, 4H), 7.22 (s, 1H), 6.97 (d, *J* = 8.5 Hz, 1H), 4.46 (d, *J* = 4.6 Hz, 2H). ^13^C NMR (101 MHz, DMSO-*d_6_*) δ 162.12, 155.67, 140.89, 140.70, 138.80, 131.87, 131.68, 129.65, 128.72, 124.94, 123.90, 117.44, 109.53, 42.47. MS (ESI): *m*/*z* = 318 (M+H)^+^.

##### *N*-(2-Fluorobenzyl)-5-hydroxybenzo[b]thiophene-2-carboxamide (**5**)

The compound was synthesized according to Scheme 1 using 2-fluorobenzylamine; yield: 24.88%. The product was purified by CC (DCM/MeOH 100:1); mp 199.8–201.7 °C; ^1^H NMR (400 MHz, DMSO-*d_6_*) δ 9.57 (s, 1H), 9.19 (t, *J* = 5.6 Hz, 1H), 7.97 (s, 1H), 7.78 (d, *J* = 8.7 Hz, 1H), 7.40 (t, *J* = 7.6 Hz, 1H), 7.33 (dd, *J* = 13.9, 6.7 Hz, 1H), 7.22 (d, *J* = 2.0 Hz, 1H), 7.19 (d, *J* = 7.0 Hz, 1H), 7.17 (s, 1H), 6.97 (dd, *J* = 8.7, 2.0 Hz, 1H), 4.52 (d, *J* = 5.6 Hz, 2H). ^13^C NMR (101 MHz, DMSO-*d_6_*) δ 162.17, 160.50 (d, *J_C_*_-*F*_ = 244.6 Hz), 155.66, 140.90, 140.61, 131.69, 130.12 (d, *J_C_*_-*F*_ = 4.4 Hz), 129.46 (d, *J_C_*_-*F*_ = 8.1 Hz), 126.22 (d, *J_C_*_-*F*_ = 14.7 Hz), 125.03, 124.80 (d, *J_C_*_-*F*_ = 3.5 Hz), 123.90, 117.45, 115.56 (d, *J_C_*_-*F*_ = 21.2 Hz), 109.54, 36.99. MS (ESI): *m*/*z* = 302 (M+H)^+^.

##### *N*-(3-Fluorobenzyl)-5-hydroxybenzo[b]thiophene-2-carboxamide (**6**)

The compound was synthesized according to Scheme 1 using 3-fluorobenzylamine; yield: 13.6%. The product was purified by CC (DCM/MeOH 100:1); mp 176.7–177.7 °C; ^1^H NMR (400 MHz, DMSO-*d_6_*) δ 9.59 (s, 1H), 9.25 (s, 1H), 7.96 (s, 1H), 7.78 (d, *J* = 8.1 Hz, 1H), 7.38 (dd, *J* = 13.3, 6.2 Hz, 1H), 7.23 (s, 1H), 7.17 (d, *J* = 8.6 Hz, 1H), 7.13 (s, 1H), 7.08 (t, *J* = 8.3 Hz, 1H), 6.98 (d, *J* = 8.7 Hz, 1H), 4.49 (d, *J* = 4.2 Hz, 2H). ^13^C NMR (101 MHz, DMSO-*d_6_*) δ 162.65 (d, *J_C_*_-*F*_ = 243.4 Hz), 162.19, 155.66, 142.74 (d, *J_C_*_-*F*_ = 7.0 Hz), 140.89, 140.61, 131.69, 130.74 (d, *J_C_*_-*F*_ = 8.2 Hz), 125.00, 123.91, 123.72 (d, *J_C_*_-*F*_ = 2.6 Hz), 117.46, 114.39 (d, *J_C_*_-*F*_ = 21.6 Hz), 114.08 (d, *J_C_*_-*F*_ = 20.9 Hz), 109.55, 42.64. MS (ESI): *m*/*z* = 302 (M+H)^+^.

##### *N*-(4-Fluorobenzyl)-5-hydroxybenzo[b]thiophene-2-carboxamide (**7**)

The compound was synthesized according to Scheme 1 using 4-fluorobenzylamine; yield: 24.88%. The product was purified by CC (DCM/MeOH 100:1.5); mp 195–197.2 °C; ^1^H NMR (400 MHz, DMSO-*d_6_*) δ 9.56 (s, 1H), 9.21 (t, *J* = 5.8 Hz, 1H), 7.94 (s, 1H), 7.78 (d, *J* = 8.7 Hz, 1H), 7.39 (s, 1H), 7.38–7.35 (m, 1H), 7.22 (d, *J* = 1.9 Hz, 1H), 7.16 (t, *J* = 8.8 Hz, 2H), 6.97 (dd, *J* = 8.7, 2.1 Hz, 1H), 4.45 (d, *J* = 5.8 Hz, 2H). ^13^C NMR (101 MHz, DMSO-*d_6_*) δ 162.06, 161.66 (d, *J_C_*_-*F*_ = 242.2 Hz), 155.66, 140.90, 140.79, 135.93 (d, *J_C_*_-*F*_ = 3.0 Hz), 131.67, 129.79 (d, *J_C_*_-*F*_ = 8.2 Hz), 124.89, 123.89, 117.41, 115.49 (d, *J_C_*_-*F*_ = 21.3 Hz), 109.52, 42.42. MS (ESI): *m*/*z* = 302 (M+H)^+^.

##### 5-Hydroxy-*N*-(2-methylbenzyl)benzo[b]thiophene-2-carboxamide (**8**)

The compound was synthesized according to Scheme 1 using 2-methylbenzylamine; yield: 3.45%. The product was purified by CC (DCM/MeOH 100:2); mp 159.1–161.2 °C; ^1^H NMR (400 MHz, DMSO-*d_6_*) δ 9.55 (s, 1H), 9.05 (s, 1H), 7.98 (s, 1H), 7.77 (d, *J* = 7.1 Hz, 1H), 7.27 (s, 1H), 7.18 (s, 3H), 6.96 (d, *J* = 7.2 Hz, 2H), 4.45 (s, 2H), 2.33 (s, 3H). ^13^C NMR (101 MHz, DMSO-*d_6_*) δ 161.98, 155.64, 140.94, 140.89, 137.21, 136.07, 131.65, 130.38, 128.07, 127.37, 126.20, 124.85, 123.88, 117.36, 109.51, 41.22, 19.17. MS (ESI): *m*/*z* = 298 (M+H)^+^.

##### 5-Hydroxy-*N*-(3-methylbenzyl)benzo[b]thiophene-2-carboxamide (**9**)

The compound was synthesized according to Scheme 1 using 3-methylbenzylamine; yield: 50.40%. The product was purified by CC (DCM/MeOH 100:2); mp 176.3–178.4 °C; ^1^H NMR (400 MHz, DMSO-*d_6_*) δ 9.59 (s, 1H), 9.17 (t, *J* = 5.9 Hz, 1H), 7.94 (s, 1H), 7.77 (d, *J* = 8.7 Hz, 1H), 7.21 (t, *J* = 7.4 Hz, 2H), 7.12 (d, *J* = 11.2 Hz, 2H), 7.06 (d, *J* = 7.4 Hz, 1H), 6.97 (dd, *J* = 8.7, 2.3 Hz, 1H), 4.43 (d, *J* = 5.9 Hz, 2H), 2.28 (s, 3H). ^13^C NMR (101 MHz, DMSO-*d_6_*) δ 162.02, 155.63, 140.92, 140.91, 139.61, 137.84, 131.67, 128.69, 128.37, 127.95, 124.89, 124.80, 123.89, 117.39, 109.52, 43.07, 21.46. MS (ESI): *m*/*z* = 298 (M+H)^+^.

##### 5-Hydroxy-*N*-(4-methylbenzyl)benzo[b]thiophene-2-carboxamide (**10**)

The compound was synthesized according to Scheme 1 using 4-methylbenzylamine; yield: 36.99%. The product was purified by CC (DCM/MeOH 100:1.5); mp 186.6–187.6 °C; ^1^H NMR (400 MHz, DMSO-*d_6_*) δ 9.56 (s, 1H), 9.16 (s, 1H), 7.94 (s, 1H), 7.77 (d, *J* = 8.7 Hz, 1H), 7.23 (s, 1H), 7.21 (d, *J* = 2.2 Hz, 2H), 7.14 (d, *J* = 7.8 Hz, 2H), 6.97 (dd, *J* = 8.7, 2.4 Hz, 1H), 4.43 (d, *J* = 5.9 Hz, 2H), 2.27 (s, 3H). ^13^C NMR (101 MHz, DMSO-*d_6_*) δ 161.97, 155.64, 140.98, 140.92, 136.69, 136.37, 131.65, 129.29, 127.77, 124.76, 123.88, 117.36, 109.51, 42.85, 21.11. MS (ESI): *m*/*z* = 298 (M+H)^+^.

##### 5-Hydroxy-*N*-(2-methoxybenzyl)benzo[b]thiophene-2-carboxamide (**11**)

The compound was synthesized according to Scheme 1 using 2-methoxybenzylamine; yield: 76.58%. The product was purified by CC (DCM/MeOH 100:1); mp 128.5–130.7 °C; ^1^H NMR (400 MHz, DMSO-*d_6_*) δ 9.60 (s, 1H), 9.02 (t, *J* = 5.8 Hz, 1H), 7.99 (s, 1H), 7.78 (d, *J* = 8.7 Hz, 1H), 7.32–7.24 (m, 1H), 7.23 (d, *J* = 2.3 Hz, 1H), 7.21 (s, 1H), 6.98 (dd, *J* = 13.6, 5.0 Hz, 2H), 6.92 (t, *J* = 7.4 Hz, 1H), 4.45 (d, *J* = 5.8 Hz, 2H), 3.83 (s, 3H). ^13^C NMR (101 MHz, DMSO-*d_6_*)) δ 162.81 (s), 162.18 (s), 157.04 (s), 155.63 (s), 140.95 (s), 131.66 (s), 128.55 (s), 127.99 (s), 126.92 (s), 124.83 (s), 123.89 (s), 120.59 (s), 117.37 (s), 110.95 (s), 109.52 (s), 55.79 (s), 38.15 (s).

##### 5-Hydroxy-*N*-(3-methoxybenzyl)benzo[b]thiophene-2-carboxamide (**12**)

The compound was synthesized according to Scheme 1 using 3-methoxybenzylamine; yield: 44.35%. The product was purified by CC (DCM/MeOH 100:1); mp 145–146.5 °C; ^1^H NMR (400 MHz, DMSO-*d_6_*) δ 9.60 (s, 1H), 9.19 (t, *J* = 6.0 Hz, 1H), 7.95 (s, 1H), 7.78 (d, *J* = 8.7 Hz, 1H), 7.26 (d, *J* = 8.1 Hz, 1H), 7.24–7.20 (m, 1H), 6.97 (dd, *J* = 8.7, 2.4 Hz, 1H), 6.91 (s, 1H), 6.90 (d, *J* = 1.3 Hz, 1H), 6.82 (dd, *J* = 7.6, 2.1 Hz, 1H), 4.45 (d, *J* = 5.9 Hz, 2H), 3.73 (s, 3H). ^13^C NMR (101 MHz, DMSO-*d_6_*) δ 162.07, 159.74, 155.64, 141.30, 140.91, 140.84, 131.67, 129.87, 124.83, 123.90, 119.91, 117.40, 113.51, 112.61, 109.53, 55.43, 43.04. MS (ESI): *m*/*z* = 314 (M+H)^+^.

##### 5-Hydroxy-*N*-(4-methoxybenzyl)benzo[b]thiophene-2-carboxamide (**13**)

The compound was synthesized according to Scheme 1 using 4-methoxybenzylamine; yield: 37.65%. The product was purified by CC (DCM/MeOH 100:1); mp 176.5–178.0 °C; ^1^H NMR (400 MHz, DMSO-*d_6_*) δ 9.55 (s, 1H), 9.13 (s, 1H), 7.93 (s, 1H), 7.77 (d, *J* = 8.2 Hz, 1H), 7.26 (d, *J* = 7.1 Hz, 2H), 7.21 (s, 1H), 6.97 (d, *J* = 8.7 Hz, 1H), 6.90 (d, *J* = 7.0 Hz, 2H), 4.40 (d, *J* = 4.3 Hz, 2H), 3.73 (s, 3H). ^13^C NMR (101 MHz, DMSO-*d_6_*) δ 161.91, 158.73, 155.64, 141.04, 140.93, 131.68, 131.64, 129.18, 124.73, 123.88, 117.34, 114.17, 109.50, 55.51, 42.58. MS (ESI): *m*/*z* = 314 (M+H)^+^.

##### *N*-(3,5-Dichlorobenzyl)-5-hydroxybenzo[b]thiophene-2-carboxamide (**14**)

The compound was synthesized according to Scheme 1 using 3,5-dichlorobenzylamine; yield: 18.53%. The product was purified by CC (DCM/MeOH 100:1); mp 189.4–192.1 °C; ^1^H NMR (400 MHz, DMSO-*d_6_*) δ 9.61 (s, 1H), 9.27 (t, *J* = 6.0 Hz, 1H), 7.95 (s, 1H), 7.78 (d, *J* = 8.8 Hz, 1H), 7.50 (t, *J* = 1.9 Hz, 1H), 7.38 (d, *J* = 1.9 Hz, 2H), 7.24 (d, *J* = 2.3 Hz, 1H), 6.98 (dd, *J* = 8.7, 2.4 Hz, 1H), 4.47 (d, *J* = 5.9 Hz, 2H). ^13^C NMR (101 MHz, DMSO-*d_6_*) δ 162.30, 155.68, 144.14, 140.86, 140.26, 134.41, 131.72, 126.99, 126.55, 125.22, 123.93, 117.56, 109.59, 42.28. MS (ESI): *m*/*z* = 352.0 (M+H)^+^.

##### N-(2,3-Difluorobenzyl)-5-hydroxybenzo[b]thiophene-2-carboxamide (15)

The compound was synthesized according to Scheme 1 using 2,3-difluorobenzylamine; yield: 2.66%. The product was purified by CC (DCM/MeOH 100:0.75); mp 199.7–201.9 °C; ^1^H NMR (400 MHz, DMSO-*d_6_*) δ 9.56 (s, 1H), 9.25–9.22 (m, 1H), 8.06 (s, 1H), 7.96 (s, 1H), 7.78 (d, *J* = 7.1 Hz, 1H), 7.34 (d, *J* = 6.7 Hz, 1H), 7.22 (s, 2H), 6.98 (d, *J* = 5.2 Hz, 1H), 4.55 (s, 2H). ^13^C NMR (101 MHz, DMSO-*d_6_*) δ 162.20, 155.68, 148.01 (dd, *J_C_*_-*F*_ = 202.1, 1.7 Hz), 147.88 (dd, *J_C_*_-*F*_ = 200.7, 1.4 Hz), 140.86, 140.39, 131.70, 130.01, 128.96 (dd, *J_C_*_-*F*_ = 11.7, 2.1 Hz), 125.26 (dd, *J_C_*_-*F*_ = 5.5, 2.2 Hz), 125.10 (dd, *J_C_*_-*F*_ = 6.6, 4.0 Hz), 123.91, 117.50, 116.52 (d, *J_C_*_-*F*_ = 16.9 Hz), 109.55, 36.78. MS (ESI): *m*/*z* = 320 (M+H)^+^.

##### *N*-(2,4-Difluorobenzyl)-5-hydroxybenzo[b]thiophene-2-carboxamide (**16**)

The compound was synthesized according to Scheme 1 using 2,4-difluorobenzylamine; yield: 25.67%. The product was purified by CC (DCM/MeOH 100:1); ^1^H NMR (400 MHz, DMSO-*d_6_*) δ 9.56 (s, 1H), 9.18 (t, *J* = 5.7 Hz, 1H), 7.95 (s, 1H), 7.78 (d, *J* = 8.7 Hz, 1H), 7.45 (d, *J* = 6.8 Hz, 1H), 7.25 (dd, *J* = 10.0, 2.0 Hz, 1H), 7.21 (d, *J* = 2.2 Hz, 1H), 7.08 (td, *J* = 8.5, 1.7 Hz, 1H), 6.97 (dd, *J* = 8.7, 2.3 Hz, 1H), 4.47 (d, *J* = 5.6 Hz, 2H). ^13^C NMR (101 MHz, DMSO-*d_6_*) δ 162.16, 160.03 (dd, *J_C_*_-*F*_ = 141.4, 2.8 Hz), 159.90 (dd, *J_C_*_-*F*_ = 142.6, 1.3 Hz) 155.67, 140.87, 140.51, 131.68, 131.48 (t, *J_C_*_-*F*_ = 2.8 Hz), 125.08, 123.90, 122.61 (dd, *J_C_*_-*F*_ = 12.3, 0.8 Hz),117.47, 111.79 (dd, *J_C_*_-*F*_ = 21.1, 3.6 Hz), 109.54, 104.13 (t, *J_C_*_-*F*_ = 25.8 Hz), 36.64. MS (ESI): *m*/*z* = 320 (M+H)^+^.

##### *N*-(3,5-Difluorobenzyl)-5-hydroxybenzo[b]thiophene-2-carboxamide (**17**)

The compound was synthesized according to Scheme 1 using 3,5-difluorobenzylamine; yield: 12.21%. The product was purified by CC (DCM/MeOH 100:1); mp 198.9–201.1 °C; ^1^H NMR (400 MHz, DMSO-*d_6_*) δ 9.58 (s, 1H), 9.27 (s, 1H), 7.97 (s, 1H), 7.79 (d, *J* = 8.3 Hz, 1H), 7.24 (s, 1H), 7.12 (s, 1H), 7.05 (d, *J* = 5.8 Hz, 2H), 6.98 (d, *J* = 7.9 Hz, 1H), 4.49 (d, *J* = 3.9 Hz, 2H). ^13^C NMR (101 MHz, DMSO-*d_6_*) δ 162.81 (dd, *J_C_*_-*F*_ = 246.1, 13.3 Hz), 162.30, 155.68, 144.58 (t, *J_C_*_-*F*_ = 8.9 Hz), 140.86, 140.36, 131.71, 125.18, 123.91, 117.52, 110.68 (dd, *J_C_*_-*F*_ = 25.1, 11.8 Hz), 109.57, 102.74 (t, *J_C_*_-*F*_ = 25.9 Hz), 42.46. MS (ESI): *m*/*z* = 320 (M+H)^+^.

##### *N*-(3,5-Bis(trifluoromethyl)benzyl)-5-hydroxybenzo[b]thiophene-2-carboxamide (**18**)

The compound was synthesized according to Scheme 1 using 3,5-bis-trifluoromethylbenzylamine; yield: 29.44%. The product was purified by CC (DCM/MeOH 100:1); mp 199–201 °C; ^1^H NMR (400 MHz, DMSO-*d_6_*) δ 9.58 (s, 1H), 9.35 (s, 1H), 8.04 (s, 3H), 7.96 (s, 1H), 7.79 (d, *J* = 8.5 Hz, 1H), 7.24 (s, 1H), 6.98 (d, *J* = 8.6 Hz, 1H), 4.65 (d, *J* = 2.8 Hz, 2H). ^13^C NMR (101 MHz, DMSO-*d_6_*) δ 162.42, 155.71, 143.36, 140.84, 140.12, 131.70, 130.66 (d, *J_C_*_-*F*_ = 32.8 Hz), 128.71, 125.15, 124.62 (d, *J_C_*_-*F*_ = 138.2 Hz), 122.44, 121.23, 117.59, 109.60, 42.48. MS (ESI): *m*/*z* = 420 (M+H)^+^.

##### *N*-(3,5-Dimethylbenzyl)-5-hydroxybenzo[b]thiophene-2-carboxamide (**19**)

The compound was synthesized according to Scheme 1 using 3,5-dimethylbenzylamine; yield: 35.47%. The product was purified by CC (DCM/MeOH 100:1); mp 140.2–141.8 °C; ^1^H NMR (400 MHz, DMSO-*d_6_*) δ 9.55 (s, 1H), 9.13 (s, 1H), 7.95 (s, 1H), 7.77 (d, *J* = 6.6 Hz, 1H), 7.21 (s, 1H), 6.93 (s, 3H), 6.88 (s, 1H), 4.39 (s, 2H), 2.25 (s, 6H). ^13^C NMR (101 MHz, DMSO-*d_6_*) δ 161.92, 155.64, 140.98, 140.93, 139.54, 137.70, 131.65, 128.71, 125.58, 124.77, 123.88, 117.36, 109.51, 43.03, 21.36. MS (ESI): *m*/*z* = 312 (M+H)^+^.

##### *N*-(2,3-Dimethoxybenzyl)-5-hydroxybenzo[b]thiophene-2-carboxamide (**20**)

The compound was synthesized according to Scheme 1 using 2,3-dimethoxybenzylamine; yield: 27.40%. The product was purified by CC (DCM/MeOH 100:1); mp 181.1–183.3 °C; ^1^H NMR (400 MHz, DMSO-*d_6_*) δ 9.55 (s, 1H), 9.05 (s, 1H), 7.97 (s, 1H), 7.77 (d, *J* = 8.1 Hz, 1H), 7.21 (s, 1H), 7.02 (d, *J* = 6.9 Hz, 1H), 6.96 (d, *J* = 8.1 Hz, 2H), 6.87 (s, 1H), 4.47 (s, 2H), 3.80 (s, 3H), 3.77 (s, 3H). ^13^C NMR (101 MHz, DMSO-*d_6_*) δ 162.04, 155.64, 152.74, 146.67, 140.98, 140.94, 132.83, 131.64, 124.80, 124.25, 123.88, 120.51, 117.35, 112.18, 109.50, 60.51, 56.14, 37.94. MS (ESI): *m*/*z* = 342.0 (M–H)^−^.

##### *N*-(2,4-Dimethoxybenzyl)-5-hydroxybenzo[b]thiophene-2-carboxamide (**21**)

The compound was synthesized according to Scheme 1 using 2,4-dimethoxybenzylamine; yield: 25.33%. The product was purified by CC (DCM/MeOH 100:1); mp 163.3–164.5 °C; ^1^H NMR (400 MHz, DMSO-*d_6_*) δ 9.54 (s, 1H), 8.90 (t, *J* = 5.7 Hz, 1H), 7.97 (s, 1H), 7.76 (d, *J* = 8.7 Hz, 1H), 7.20 (d, *J* = 2.3 Hz, 1H), 7.13 (d, *J* = 8.3 Hz, 1H), 6.96 (dd, *J* = 8.7, 2.4 Hz, 1H), 6.56 (d, *J* = 2.4 Hz, 1H), 6.49 (dd, *J* = 8.4, 2.4 Hz, 1H), 4.36 (d, *J* = 5.7 Hz, 2H), 3.81 (s, 3H), 3.74 (s, 3H). ^13^C NMR (101 MHz, DMSO-*d_6_*) δ 162.02, 160.17, 158.08, 155.62, 141.11, 140.97, 131.63, 129.18, 124.73, 123.86, 119.12, 117.30, 109.49, 104.82, 98.67, 55.88, 55.64, 37.86. MS (ESI): *m*/*z* = 342.0 (M–H)^−^.

##### *N*-(3,4-Dimethoxybenzyl)-5-hydroxybenzo[b]thiophene-2-carboxamide (**22**)

The compound was synthesized according to Scheme 1 using 3,4-dimethoxybenzylamine; yield: 17.97%. The product was purified by CC (DCM/MeOH 100:1); mp 188.3–190.3 °C; ^1^H NMR (400 MHz, DMSO-*d_6_*) δ 9.58 (s, 1H), 9.12 (s, 1H), 7.93 (s, 1H), 7.77 (d, *J* = 8.2 Hz, 1H), 7.21 (s, 1H), 6.96 (d, *J* = 8.3 Hz, 2H), 6.90 (d, *J* = 7.6 Hz, 1H), 6.86 (s, 1H), 4.40 (s, 2H), 3.73 (s, 3H), 3.72 (s, 3H). ^13^C NMR (101 MHz, DMSO-*d_6_*) δ 161.95, 155.62, 149.07, 148.30, 140.99, 140.92, 132.09, 131.64, 124.74, 123.88, 120.01, 117.36, 112.22, 112.01, 109.51, 56.01, 55.89, 42.93. MS (ESI): *m*/*z* = 344 (M+H)^+^.

##### *N*-(3,5-Dimethoxybenzyl)-5-hydroxybenzo[b]thiophene-2-carboxamide (**23**)

The compound was synthesized according to Scheme 1 using 3,5-dimethoxybenzylamine; yield: 29.12%. The product was purified by CC (DCM/MeOH 100:1); ^1^H NMR (400 MHz, DMSO-*d_6_*) δ 9.56 (s, 1H), 9.16 (s, 1H), 7.96 (s, 1H), 7.77 (d, *J* = 8.5 Hz, 1H), 7.22 (s, 1H), 6.97 (d, *J* = 8.7 Hz, 1H), 6.49 (s, 2H), 6.39 (s, 1H), 4.40 (d, *J* = 3.5 Hz, 2H), 3.72 (s, 6H). ^13^C NMR (101 MHz, DMSO-*d_6_*) δ 162.05, 160.92, 155.65, 142.08, 140.91, 140.84, 131.65, 124.85, 123.89, 117.39, 109.53, 105.79, 98.92, 55.57, 43.14. MS (ESI): *m*/*z* = 344 (M+H)^+^.

##### (3,4-Dihydroisoquinolin-2(1H)-yl)(5-hydroxybenzo[b]thiophen-2-yl)methanone (**24**)

The compound was synthesized according to Scheme 1 using 1,2,3,4-tetrahydro-isoquinoline; yield: 30.18%. The product was purified by CC (DCM/MeOH 100:1); mp 198.8–200.8 °C; ^1^H NMR (400 MHz, DMSO-*d_6_*) δ 9.59 (s, 1H), 7.78 (d, *J* = 8.7 Hz, 1H), 7.68 (s, 1H), 7.27 (d, *J* = 2.3 Hz, 1H), 7.20 (s, 4H), 6.97 (dd, *J* = 8.7, 2.4 Hz, 1H), 4.83 (s, 2H), 3.88 (t, *J* = 5.5 Hz, 2H), 2.93 (t, *J* = 5.5 Hz, 2H). ^13^C NMR (101 MHz, DMSO-*d_6_*) δ 163.46, 155.71, 140.54, 138.30, 134.96, 133.50, 130.72, 128.94, 127.05, 126.70, 126.58, 126.36, 123.59, 117.12, 109.61, 45.69, 41.27, 35.33. MS (ESI): *m*/*z* = 310.1 (M+H)^+^.

##### (5-Chloroisoindolin-2-yl)(5-hydroxybenzo[b]thiophen-2-yl)methanone (**25**)

The compound was synthesized according to Scheme 1 using 5-chloro-2,3-dihydro-1*H*-isoindole; yield: 33%. The product was purified by CC (DCM/MeOH 100:1); mp 224.9–226.0 °C; ^1^H NMR (400 MHz, DMSO-*d_6_*) δ 9.61 (s, 1H), 7.95 (s, 1H), 7.79 (s, 1H), 7.52 (s, 1H), 7.44 (s, 1H), 7.39 (s, 1H), 7.29 (s, 1H), 7.00 (s, 1H), 5.23 (s, 2H), 4.89 (s, 2H). ^13^C NMR (101 MHz, DMSO-*d_6_*) δ 161.78, 155.66, 141.23, 139.93, 138.29, 134.92, 131.27, 128.01, 126.75, 125.13, 125.03, 123.56, 123.33, 117.63, 109.74, 54.16, 53.98. MS (ESI): *m*/*z* = 330.07 (M+H)^+^.

##### 5-Hydroxy-*N*-(pyridin-3-ylmethyl)benzo[b]thiophene-2-carboxamide (**26**)

The compound was synthesized according to Scheme 1 using 3-aminomethylpyridine; yield: 100%. The product was purified by CC (DCM/MeOH 100:6.5); mp 198.0–200.8 °C; ^1^H NMR (400 MHz, DMSO-*d_6_*) δ 9.56 (s, 1H), 9.25 (s, 1H), 8.57 (s, 1H), 8.47 (s, 1H), 7.94 (s, 1H), 7.76 (dd, *J* = 13.9, 8.2 Hz, 2H), 7.35 (d, *J* = 18.1 Hz, 1H), 7.22 (s, 1H), 6.97 (d, *J* = 8.2 Hz, 1H), 4.50 (s, 2H). ^13^C NMR (101 MHz, DMSO-*d_6_*) δ 162.20, 155.67, 149.34, 148.65, 140.87, 140.54, 135.69, 135.18, 131.67, 125.03, 123.96, 123.91, 117.46, 109.54, 40.89. MS (ESI): *m*/*z* = 283 (M-H)^−^.

##### 5-Hydroxy-*N*-(thiophen-2-ylmethyl)benzo[b]thiophene-2-carboxamide (**27**)

The compound was synthesized according to Scheme 1 using thiophen-2-ylmethylamine; yield: 9.12%. The product was purified by CC (DCM/MeOH 100:1); mp 139.6–141.4 °C; ^1^H NMR (400 MHz, DMSO-*d_6_*) δ 9.33 (s, 1H), 9.06 (s, 1H), 7.69 (s, 2H), 7.55 (s, 1H), 7.17 (s, 1H), 6.98 (s, 1H), 6.78 (d, *J* = 19.0 Hz, 2H), 4.40 (s, 2H). ^13^C NMR (101 MHz, DMSO-*d_6_*) δ 162.72, 161.88, 155.66, 142.58, 140.86, 131.68, 127.11, 126.09, 125.58, 124.99, 123.89, 117.45, 109.53, 38.16. MS (ESI): *m*/*z* = 288 (M–H)^−^.

##### *N*-(Furan-2-Ylmethyl)-5-hydroxybenzo[b]thiophene-2-carboxamide (**28**)

The compound was synthesized according to Scheme 1 using furan-2-ylmethylamine: yield; 17.19%. The product was purified by CC (DCM/MeOH 100:1); mp 99.2–101.6 °C; ^1^H NMR (400 MHz, DMSO-*d_6_*) δ 9.55 (s, 1H), 9.14 (t, *J* = 5.7 Hz, 1H), 7.94 (s, 1H), 7.77 (d, *J* = 8.7 Hz, 1H), 7.59 (dd, *J* = 1.7, 0.8 Hz, 1H), 7.20 (d, *J* = 2.3 Hz, 1H), 6.97 (dd, *J* = 8.7, 2.4 Hz, 1H), 6.41 (dd, *J* = 3.2, 1.9 Hz, 1H), 6.31 (dd, *J* = 3.1, 0.5 Hz, 1H), 4.46 (d, *J* = 5.6 Hz, 2H). ^13^C NMR (101 MHz, DMSO-*d_6_*) δ 161.91, 155.65, 152.43, 142.61, 140.89, 140.65, 131.68, 125.01, 123.89, 117.43, 110.94, 109.54, 107.60, 36.43. MS (ESI): *m*/*z* = 272.03 (M–H)^−^.

##### 5-Hydroxy-*N*-phenethylbenzo[b]thiophene-2-carboxamide (**29**)

The compound was synthesized according to Scheme 1 using phenethylamine; yield: 13% The product was purified by CC (DCM/MeOH 100:1); mp 99.2–101.1 °C; ^1^H NMR (400 MHz, DMSO-*d_6_*) δ 9.54 (s, 1H), 8.74 (s, 1H), 7.86 (s, 1H), 7.76 (d, *J* = 8.5 Hz, 1H), 7.29 (d, *J* = 6.8 Hz, 2H), 7.25 (d, *J* = 6.8 Hz, 3H), 7.21 (s, 1H), 6.96 (d, *J* = 8.6 Hz, 1H), 3.48 (d, *J* = 5.9 Hz, 2H), 2.85 (t, *J* = 6.6 Hz, 2H). ^13^C NMR (101 MHz, DMSO-*d_6_*) δ 161.95, 155.63, 141.16, 140.89, 139.80, 131.56, 129.09, 128.80, 126.57, 124.46, 123.87, 117.28, 109.47, 41.32, 35.49. MS (ESI): *m*/*z* = 298.16 (M+H)^+^.

##### *N*-(2-(1H-Indol-3-yl)ethyl)-5-hydroxybenzo[b]thiophene-2-carboxamide (**30**)

The compound was synthesized according to Scheme 1 using 2-(1*H*-Indol-3-yl)ethylamine; yield: 9.54%. The product was purified by CC (DCM/MeOH 100:3); mp 211.4–213.0 °C; ^1^H NMR (400 MHz, DMSO-*d_6_*) δ 10.81 (s, 1H), 9.54 (s, 1H), 8.78 (t, *J* = 5.5 Hz, 1H), 7.87 (s, 1H), 7.77 (d, *J* = 8.7 Hz, 1H), 7.59 (d, *J* = 7.8 Hz, 1H), 7.34 (d, *J* = 8.0 Hz, 1H), 7.24–7.16 (m, 2H), 7.07 (t, *J* = 7.3 Hz, 1H), 7.02–6.94 (m, 2H), 3.59–3.48 (m, 2H), 2.96 (t, *J* = 7.4 Hz, 2H). ^13^C NMR (101 MHz, DMSO-*d_6_*) δ 161.95, 155.63, 141.35, 140.92, 136.68, 131.57, 127.67, 124.44, 123.88, 123.10, 121.37, 118.70, 118.68, 117.25, 112.14, 111.82, 109.46, 40.66, 25.59. MS (ESI): *m*/*z* = 337.27 (M–H)^−^.

### 4.2. Biological Assays

#### 4.2.1. Protein Kinases and Inhibition Assays

Assays are described in Supporting Information.

#### 4.2.2. Cell Culture

All cells were purchased from Bioresource Collection and Research Center (Hsinchu, Taiwan) and cultured in corresponding media supplemented with 10% fetal bovine serum, 1 × Antibiotic-Antimycotic at 37 °C in a humidified incubator with 5% CO_2_. HCT-116, MCF-7, A549, U87, Hela, and HaCaT cells were cultured in DMEM media. T24 cells were cultured in McCoy’s 5a media. IEC-6 cells were cultured in DMEM media supplemented with 5% fetal bovine serum, 1 × Antibiotic–Antimycotic, and 0.1 unit/mL insulin.

#### 4.2.3. Cell Viability

Cells were incubated with the Cell Counting Kit-8 (CCK-8) reagent (TargetMol, Boston, MA, USA) at 37 °C for 2 h. The cell viability was determined by spectrophotometer at 450 nm.

#### 4.2.4. Cell Cycle Assay

Cells were fixed with 75% ethanol and then stained with 50 μg/mL propidium iodide (PI; Cat# P4170, Sigma-Aldrich, St. Louis, MO, USA) at room temperature for 1 h. Cell cycle was analyzed by flow cytometry.

#### 4.2.5. Apoptosis Assay

Apoptosis was examined by Annexin V-FITC/PI Apoptosis Kit (Cat# E-CK-A211, Elabscience, Houston, TX, USA) following the manufacturer’s protocol and analyzed by flow cytometry.

#### 4.2.6. Western Blot

Cell lysates were sequentially suspended in commercial RIPA Buffer (pH 7.4; Cat# RB4475, Bio Basic, Markham, Ontario, Canada) containing Protease Inhibitor Cocktail and PhosSTOP (Merck, Darmstadt, Germany), boiled in sodium dodecyl sulphate (SDS) sample buffer, separated by SDS-polyacrylamide gel electrophoresis (PAGE), and electrophoresed onto a PVDF membrane. Antibodies used in this study were Bcl-2 (Cat#A19693, RRID: AB_2862738) and Bax (Cat# A0207, RRID: AB_2757021) from ABclonal (Boston, MA, USA); cleaved caspase-3 (Cat# 9662, RRID: AB_331439) and GAPDH (Cat# 8884, RRID:AB_11129865) from Cell Signaling Technology (Beverly, MA, USA). The labeled signals were determined using HRP-conjugated secondary antibodies and the Bio-rad ChemiDoc™ MP Imaging System (Berkeley, CA, USA).

## Figures and Tables

**Table 1:** Inhibition of Dyrk1A/Dyrk1B/Clk1 kinases (compounds **1**–**30**).

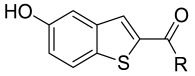							
Cpd. No.	R	Dyrk1A		Dyrk1B		Clk1	
		% Inh. At 1 µM ^a^	IC_50_ (nM) ^a^	% Inh. At 1 µM ^a^	IC_50_ (nM) ^a^	% Inh. At 1 µM ^a^	IC_50_ (nM) ^a^
**1**	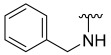	46	ND	60	833	60	740
**2**	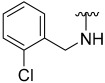	61	700	46	ND	61	769
**3**	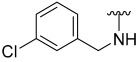	60	751	66	530	79	257
**4**	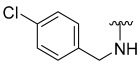	7	ND	0	ND	40	ND
**5**	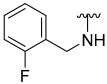	48	ND	62	722	63	778
**6**	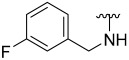	60	666	71	406	72	410
**7**	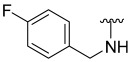	26	ND	38	ND	50	ND
**8**	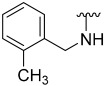	61	853	48	ND	63	594
**9**	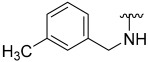	33	ND	40	ND	61	787
**10**	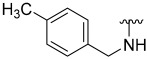	8	ND	0	ND	30	ND
**11**	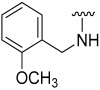	64	565	64	578	60	725
**12**	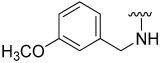	65	536	81	238	86	153
**13**	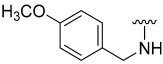	9	ND	16	ND	17	ND
**14**	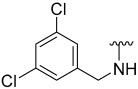	41	ND	12	ND	61	744
**15**	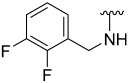	53	ND	61	645	61	779
**16**	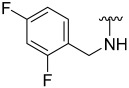	40	ND	53	ND	50	ND
**17**	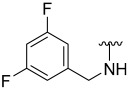	67	495	81	242	85	168
**18**	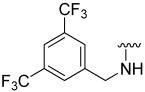	8	ND	0	ND	26	ND
**19**	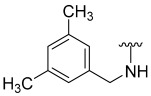	22	ND	0	ND	53	ND
**20**	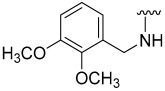	14	ND	19	ND	21	ND
**21**	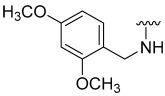	9	ND	17	ND	43	ND
**22**	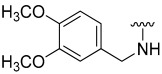	8	ND	16	ND	18	ND
**23**	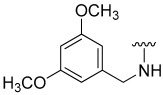	12	ND	22	ND	22	ND
**24**	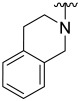	12	ND	21	ND	33	ND
**25**	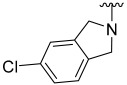	13	ND	24	ND	62	608
**26**	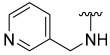	34	ND	39	ND	47	ND
**27**	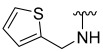	53	ND	61	703	60	705
**28**	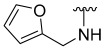	66	779	52	ND	61	585
**29**	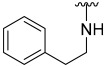	29	ND	48	ND	61	753
**30**	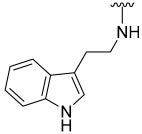	20	ND	40	ND	74	350

^a^ Values are mean values of at least two experiments; standard deviation <10%; the assay was carried out at an ATP conc. equivalent to the K_m_ of the tested kinase; ND: not determined.

**Table 2:** Testing against Clk2, Clk3, and Haspin.

Cpd No.	% Inhibition at 1 µM ^a^ (IC_50_ in nM) ^b^		
	Clk2	Clk3	Haspin
**3**	46	22	54
**6**	38	27	83 (207)
**11**	50	24	46
**12**	62 (617)	26	85 (167)
**17**	62 (708)	26	80 (256)

^a^ Screenings were performed as a service by Thermo Fisher Scientific (Massachusetts, USA). Inhibition values represent mean values of duplicates that differed by less than 10%. ^b^ IC_50_ values were determined for inhibition values >60% at an ATP concentration equivalent to the K_m_ of the tested kinase.

**Table 3:** In vitro cell viability inhibition of cancer cell lines.

Cpd.	HCT-116 % Inh. at 20 μM ^a^ (IC_50_ in µM) ^b^	MCF-7 % Inh. at μM ^a^ (IC_50_ in µM) ^b^	T24 % Inh. at μM ^a^ (IC_50_ in µM) ^b^	A549 % Inh. at μM ^a^ (IC_50_ in µM) ^b^	U87 % Inh. at μM ^a^ (IC_50_ in µM) ^b^	Hela % Inh. at μM ^a^ (IC_50_ in µM) ^b^
**3**	25.9 ± 2.54	15.6 ± 3.85	68.6 ± 3.95 (14)	54.7 ± 3.77	32.17 ± 4.76	50.5 ± 3.93
**6**	60.1 ± 3.11	16.2 ± 2.20	49.3 ± 6.04	36.5 ± 1.57	21.04 ± 5.07	53.3 ± 2.22
**11**	0	56.4 ± 4.47	55.3 ± 3.30	34.7 ± 3.91	18.09 ± 0.81	62.1 ± 1.97 (19.3)
**12**	75.7 ± 0.31 (6.4)	93.5 ± 0.17	100 (6)	41.0 ± 8.96	60.12 ± 6.16 (16.5)	50.7 ± 2.41
**17**	67.6 ± 0.58 (8.1)	20.9 ± 2.02	59.9 ± 2.05 (14.9)	38.8 ± 4.06	47.28 ± 5.65	60.9 ± 2.82 (18.1)

^a^ Cells were treated with vehicle (DMSO, 0.1%) or indicated compounds (20 μM). Cell viability was determined using a CCK-8 assay following 72 h of treatment. The inhibitory percentage (% Inh.) is expressed as mean ± S.E.M. (n = 3). ^b^ IC_50_ is defined as the concentration necessary for 50% inhibition of viability.

**Table 4:** Effects of compounds on cell viability in normal cells.

Cpd.	IEC-6				HaCaT			
	Viability (%) ^a^			IC_50_ (μM) ^b^	Viability (%) ^a^			IC_50_ (μM) ^b^
**12**	95.99	±	2.22	>20	67.63	±	6.09	>20
**17**	76.83	±	3.00	>20	64.90	±	1.97	>20

^a^ Percentage of cell viability of 20 μM. Results are expressed as mean ± S.E.M. (n = 3). *p* < 0.001 compared with DMSO alone. ^b^ Concentration necessary for 50% inhibition (IC_50_).

## Data Availability

All data underlying the results are available as part of the article and no additional source data are required.

## Supplementary Materials

The following supporting information can be downloaded at: https://www.mdpi.com/article/10.3390/cancers16112033/s1, Table S1: The expression levels of targeted kinases in selected cancer cell lines according to the protein Atlas database, ^1^H NMR, ^13^C NMR, UV and mass charts of representative compounds, Experimental procedure for protein kinases and inhibition assays, Dose–response curves, Uncropped Western blot figures.

## Abbreviations

Bax, Bcl-2 Associated X-protein; Bcl-2, B-cell lymphoma 2; Clk, CDC-like kinase; CMGC, Cyclin-dependent kinases, Mitogen-activated protein kinases, Glycogen synthase kinases, CDC-like kinases; DREAM, Dimerization partner, RB-like, E2F and multi-vulval class B; Dyrk, Dual specificity tyrosine regulated kinase; EGCG, Epigallocatechin gallate; EGFR, Epidermal growth factor receptor; GAPDH; Glyceraldehyde 3-phosphate dehydrogenase; gk, Gatekeeper; Haspin, Haploid Germ Cell-Specific Nuclear Protein Kinase; H3T3, Haspin-mediated histone H3 threonine 3; PI, Propidium iodide; SR, Serine- and arginine-rich.
