# Supplementary material for: N-Benzylated 5-Hydroxybenzothiophene-2-carboxamides as Multi-Targeted Clk/Dyrk Inhibitors and Potential Anticancer Agents
Source: Cancers (Basel). 2024 May 27;16(11):2033. doi: 10.3390/cancers16112033 (PMC11171218; doi:10.3390/cancers16112033)
Supplement: Supplementary file 1 [file cancers-16-02033-s001.zip › cancers-2979784-supplementary.pdf]

N-Benzylated 5-Hydroxybenzothiophene-2-carboxamides as Multi-Targeted Clk/Dyrk Inhibitors and Potential Anticancer Agents

AUTHOR NAMES

Noha M. Mohamed[1,2‡], Po-Jen Chen[‡3,4], Sarah S. Darwish[1,5], Yu-Chieh Su[4,6,7], Ming-Hua Shiao[8], Gary A. Piazza[9], Ashraf H. Abadi[1], Matthias Engel[10], Mohammad Abdel-Halim*[1]*

AUTHOR ADDRESS

[1]Department of Pharmaceutical Chemistry, Faculty of Pharmacy and Biotechnology, German University in Cairo, Cairo 11835, Egypt

[2]Department of Pharmaceutical Chemistry, School of Pharmacy, Newgiza University, Cairo 12256, Egypt

[3]Department of Medical Research, E-Da Hospital, I-Shou University, Kaohsiung 824410, Taiwan

[4]Graduate Institute of Medicine, College of Medicine, I-Shou University, Kaohsiung 824410, Taiwan

[5]School of Life and Medical Sciences, University of Hertfordshire hosted by Global Academic Foundation, New Administrative Capital, 11578 Cairo, Egypt

[6]Division of Hematology-Oncology, Department of Internal Medicine, E-Da Hospital, I-Shou University, Kaohsiung 824410, Taiwan

[7]School of Medicine, College of Medicine, I-Shou University, Kaohsiung 824410, Taiwan

[8]Taiwan Instrument Research Institute, National Applied Research Laboratories, Hsinchu 300092, Taiwan

[9]Department of Drug Discovery and Development, Harrison College of Pharmacy, Auburn University, Auburn, Alabama, 36832

[10]Pharmaceutical and Medicinal Chemistry, Saarland University, Campus C2.3, D-66123 Saarbrücken, Germany

‡ Authors contributed equally to this work

*Correspondence:

Mohammad Abdel-Halim (mohammad.abdel-halim@guc.edu.eg) and Matthias Engel (ma.engel@mx.uni-saarland.de)

Table of content

1. Table S1: The expression levels of targeted kinases in selected cancer cell lines according to the protein Atlas database …………………………………………………………….……. Page S4

2. ^{1}H NMR, ^{13}C NMR, UV, and mass charts of representative compounds………………Page S5

3. Experimental procedure for protein kinases and inhibition assays…………………. Page S25

4. Dose response curves…………………………………………………………………Page S28

5. Uncropped western blot figures……………………….………………………………Page 37

1. Table S1: The expression levels of targeted kinases in selected cancer cell lines.

	Targeted Kinases				
	**Dyrk1A**	**Dyrk1B**	**Clk1**	**Clk2**	**Haspin**
**Cell line**	**Expression levels**				
**T24**	17.5	2.5	28	8.5	11.8
**Hela**	24.7	5.1	31.3	11	12.1
**MCF-7**	21.6	5.4	15.4	14	8.3
**HCT-116**	22.1	1.3	16.4	9.9	16.7
**A549**	13.7	6	20.1	8.5	12.3
**U87**	20	5.8	14.8	10.1	5.7

2. ^{1}H NMR and ^{13}C NMR UV and mass charts of representative compounds.

Compound 3

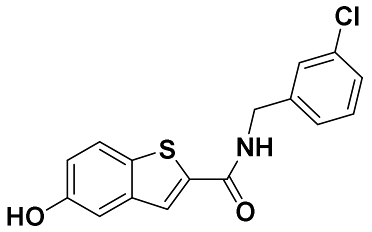

3

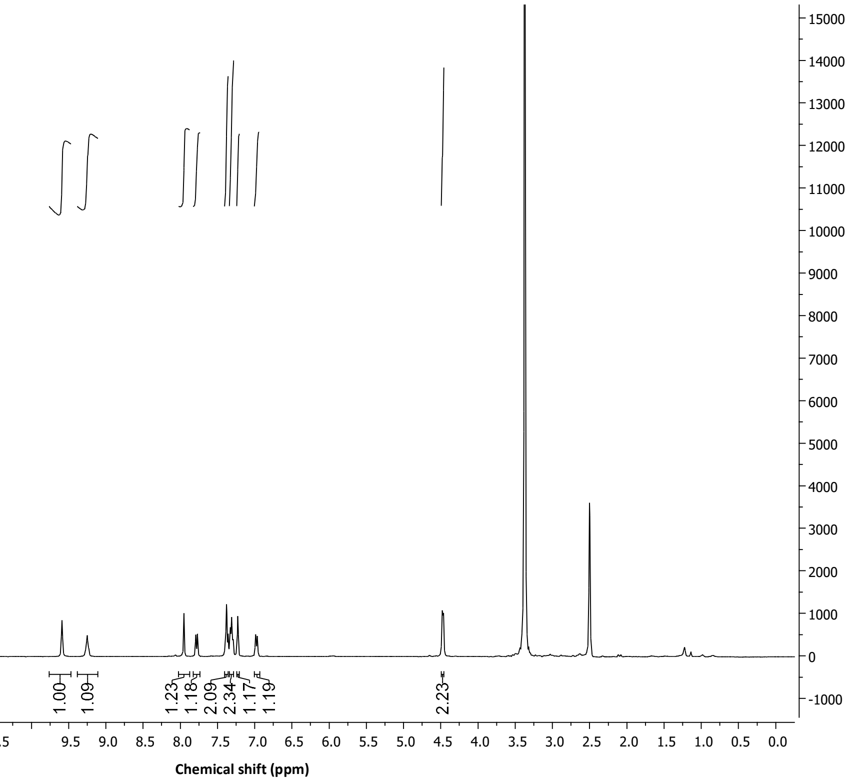

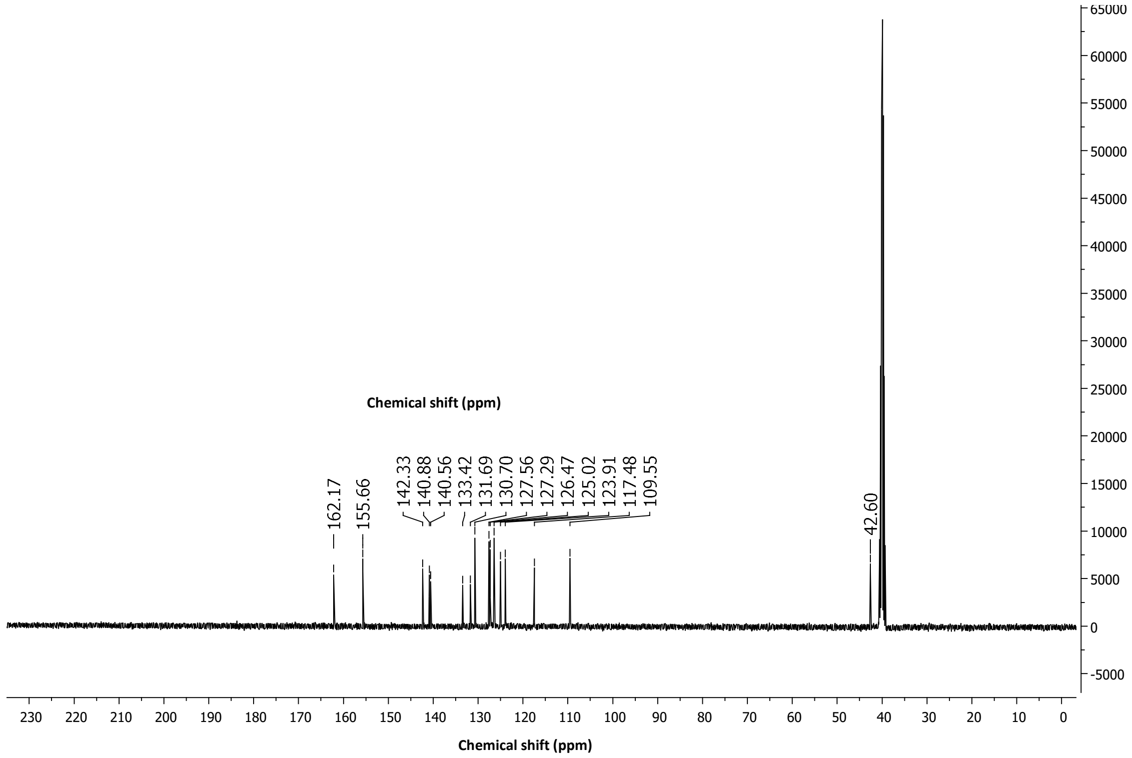

Compound 3

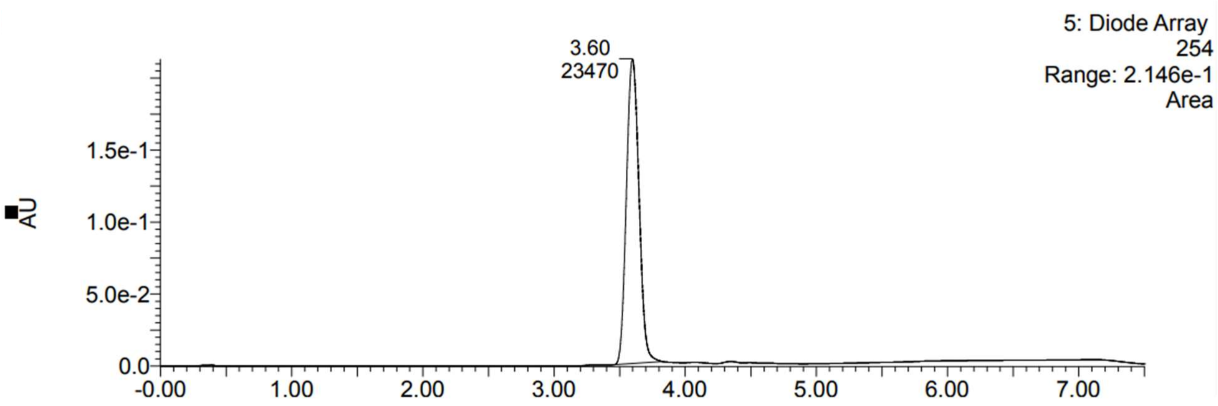
5: Diode Array
254
Range: 2.146e-1
Area
3.60
23470
AU
1.5e-1
1.0e-1
5.0e-2
0.0
-0.00
1.00
2.00
3.00
4.00
5.00
6.00
7.00

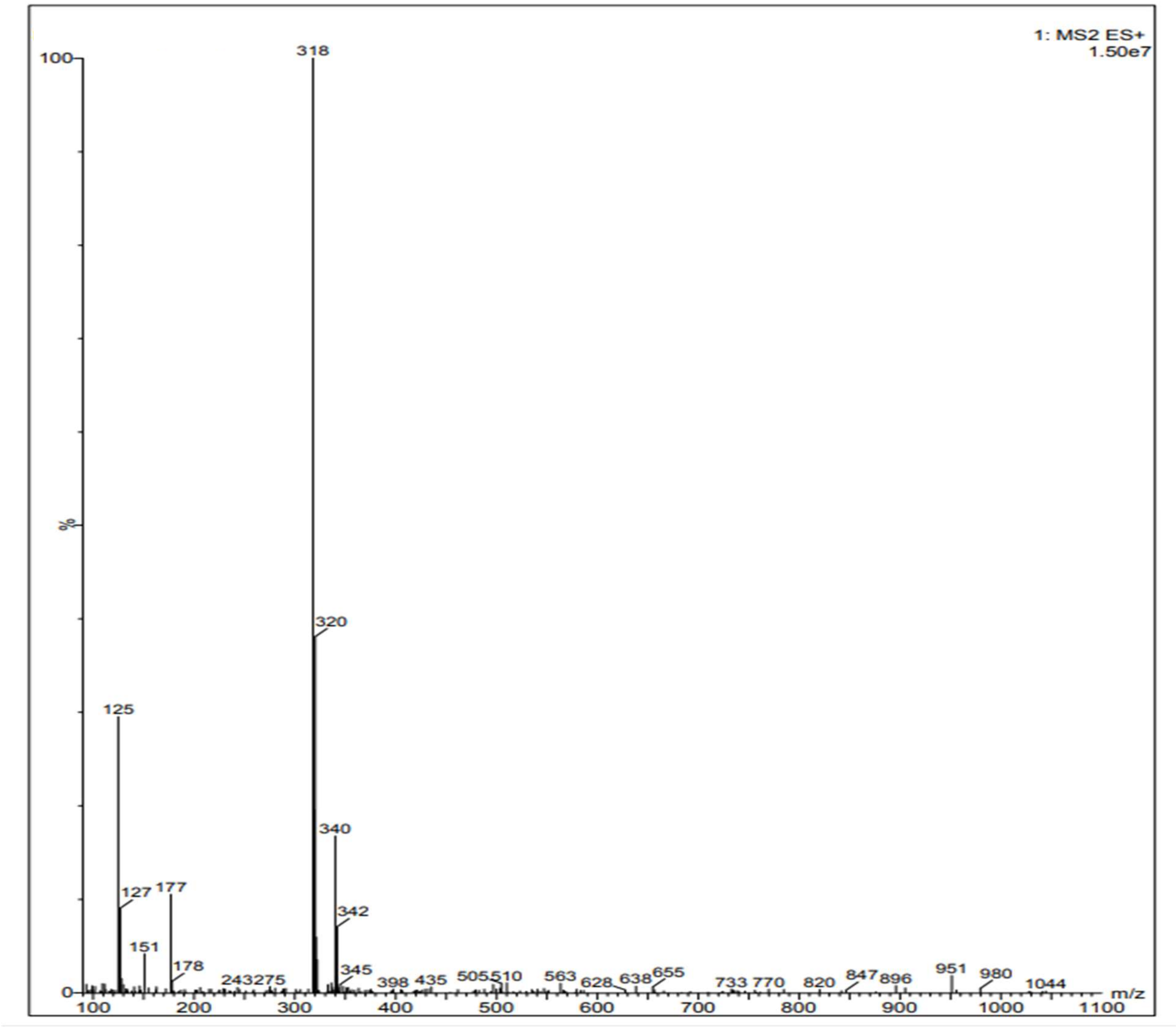
1: MS2 ES+
1.50e7
100
%
0
318
320
125
340
127
177
342
151
178
243275
345
398
435
505510
563
628
638655
733
770
820
847896
951
980
1044
m/z
100
200
300
400
500
600
700
800
900
1000
1100

Compound 5

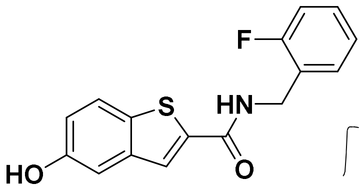
F
HN
S
HO
O

5

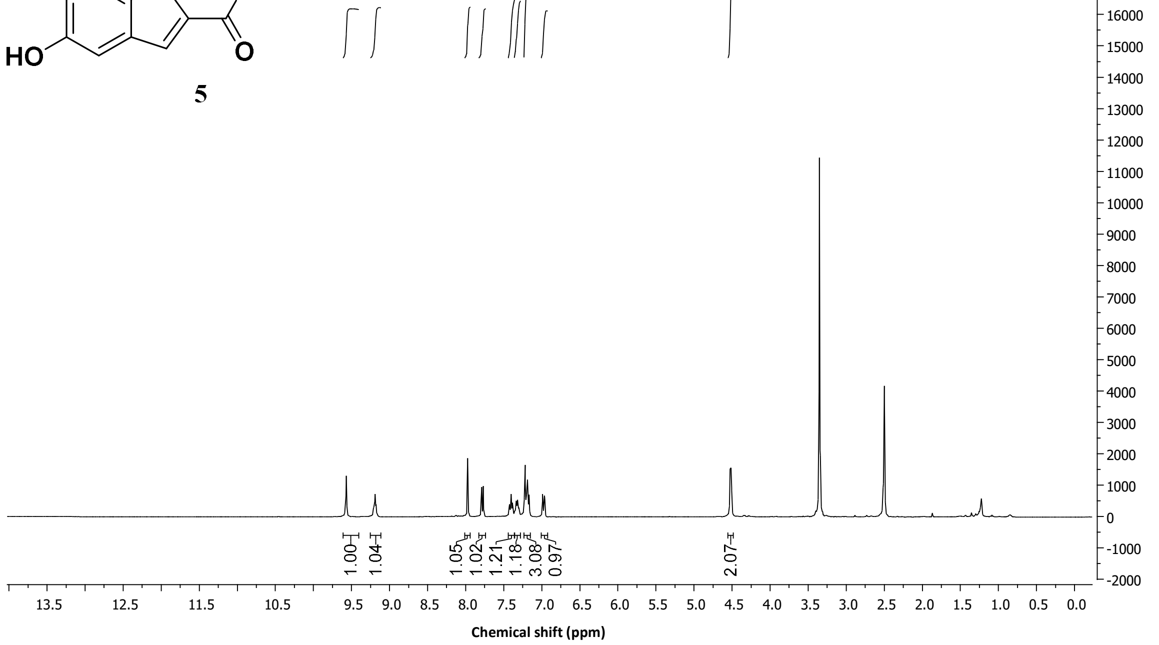
1.00
1.04
1.05
1.02
1.21
1.18
3.08
0.97
2.07
Chemical shift (ppm)

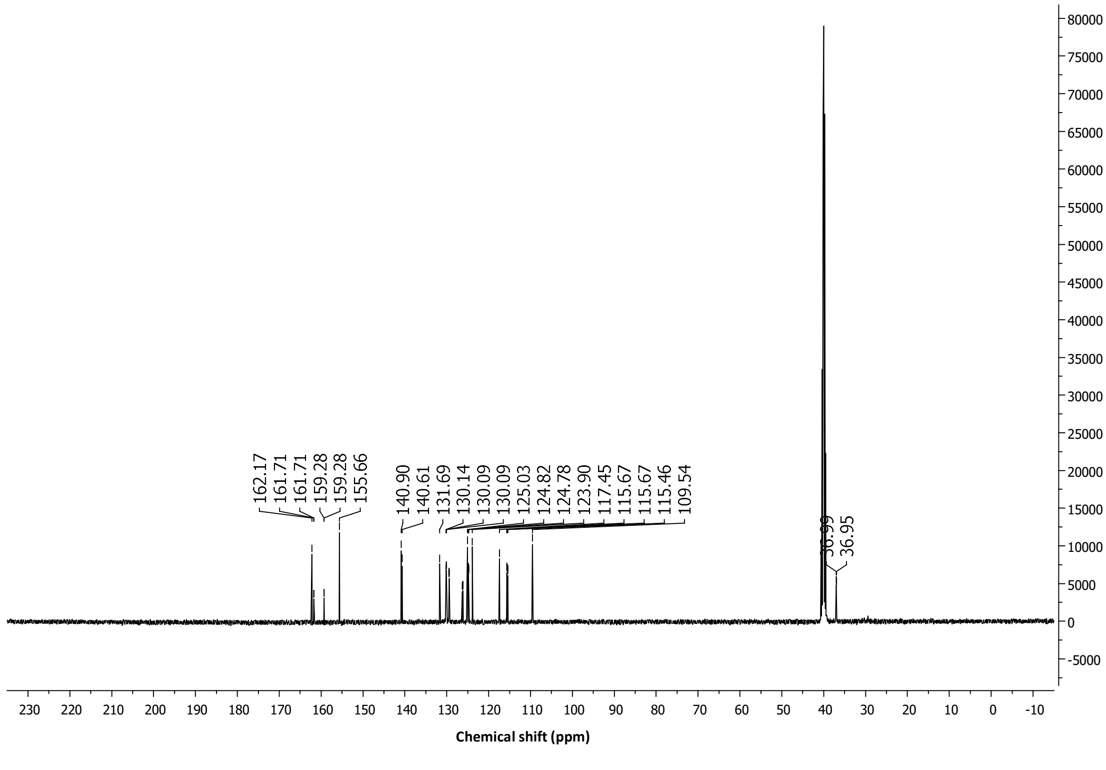
162.17
161.71
161.71
159.28
159.28
155.66
140.90
140.61
131.69
130.14
130.09
130.09
125.03
124.82
124.78
123.90
117.45
115.67
115.67
115.46
109.54
36.99
36.95
Chemical shift (ppm)

Compound 5

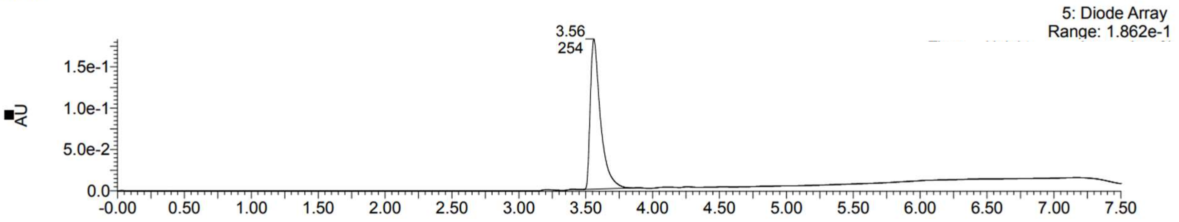

5: Diode Array
Range: 1.862e-1
3.56
254
AU
1.5e-1
1.0e-1
5.0e-2
0.0
-0.00 0.50 1.00 1.50 2.00 2.50 3.00 3.50 4.00 4.50 5.00 5.50 6.00 6.50 7.00 7.50

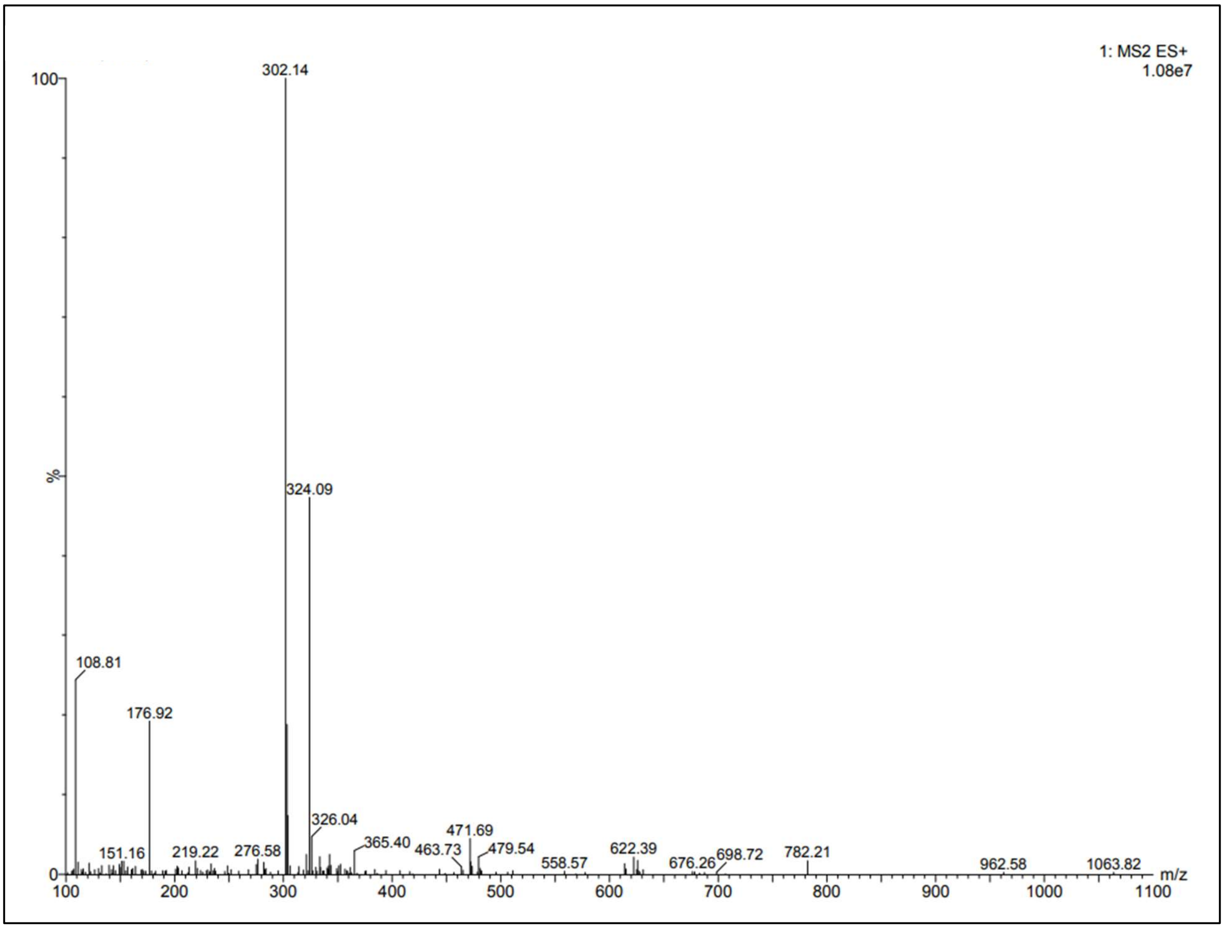

1: MS2 ES+
1.08e7
100
%
0
302.14
324.09
108.81
176.92
326.04
471.69
365.40
463.73
479.54
151.16
219.22
276.58
558.57
622.39
676.26
698.72
782.21
962.58
1063.82
m/z
100 200 300 400 500 600 700 800 900 1000 1100

Compound 6

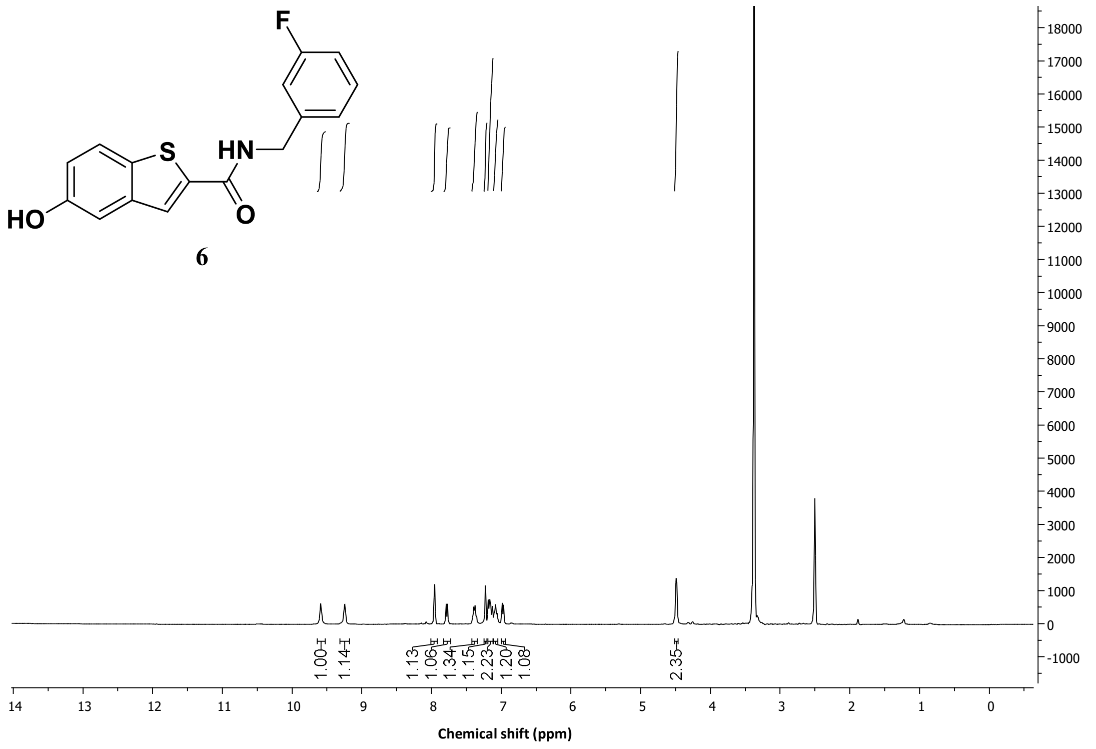
F
HN
S
HO
O
6
1.00
1.14
1.13
1.06
1.34
1.15
2.23
1.20
1.08
2.35
Chemical shift (ppm)

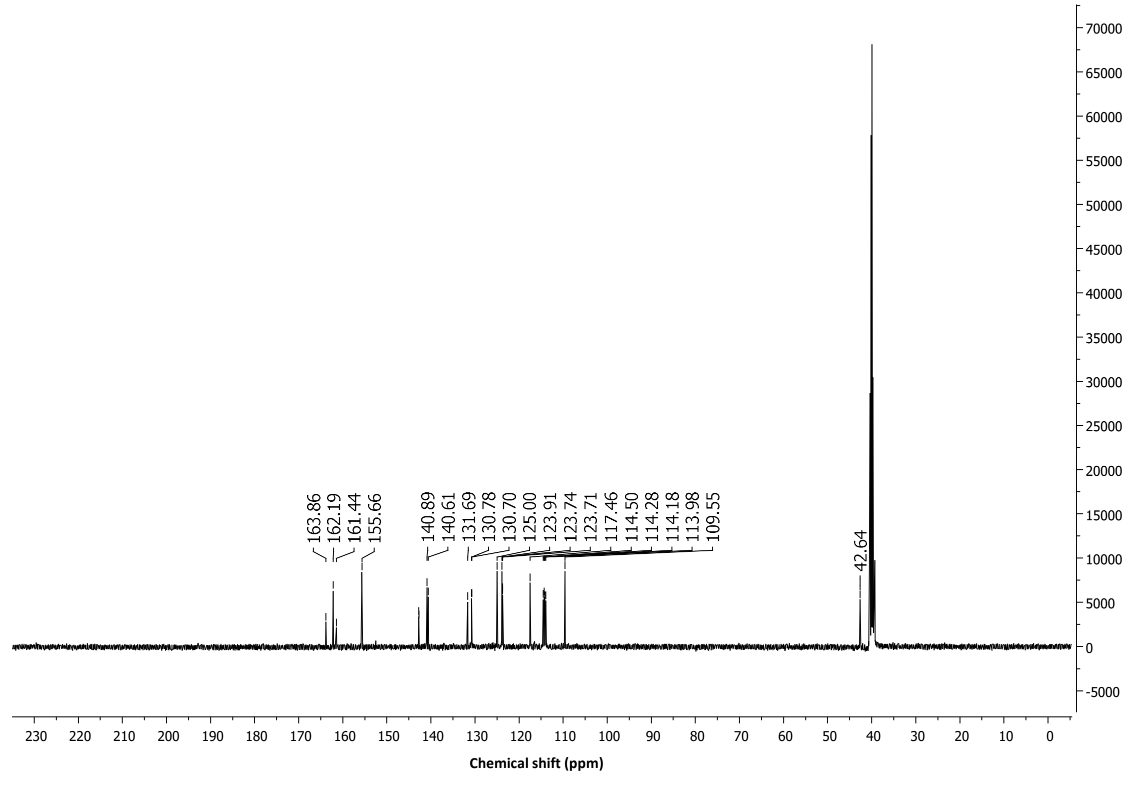
163.86
162.19
161.44
155.66
140.89
140.61
131.69
130.78
130.70
125.00
123.91
123.74
123.71
117.46
114.50
114.28
114.18
113.98
109.55
42.64
Chemical shift (ppm)

Compound 6

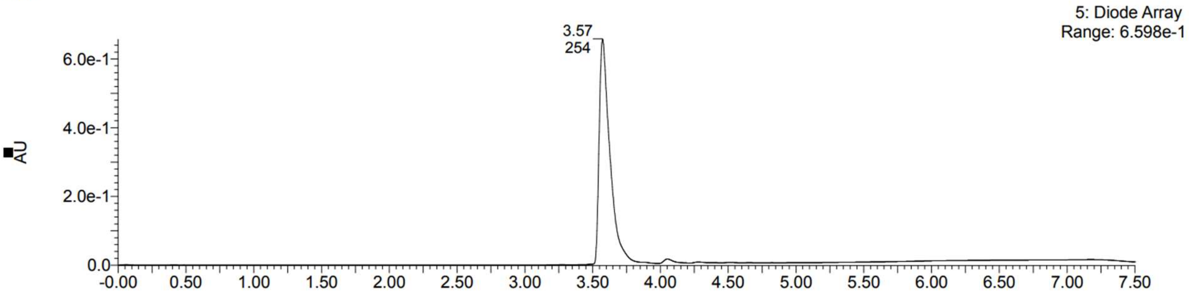

5: Diode Array
Range: 6.598e-1
3.57
254
AU

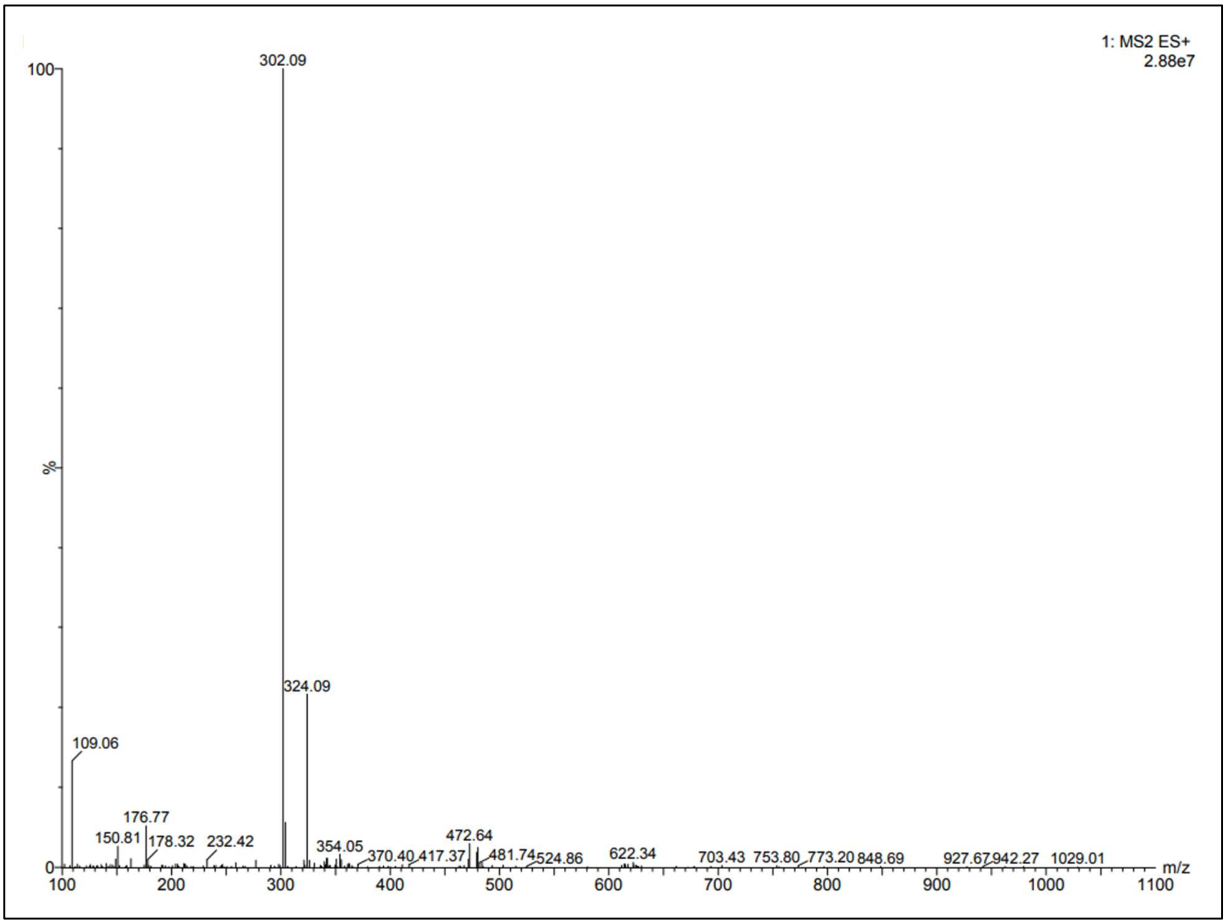

1: MS2 ES+
2.88e7
302.09
324.09
109.06
176.77
150.81
178.32
232.42
354.05
370.40
417.37
472.64
481.74
524.86
622.34
703.43
753.80
773.20
848.69
927.67
942.27
1029.01
%
m/z

Compound 8

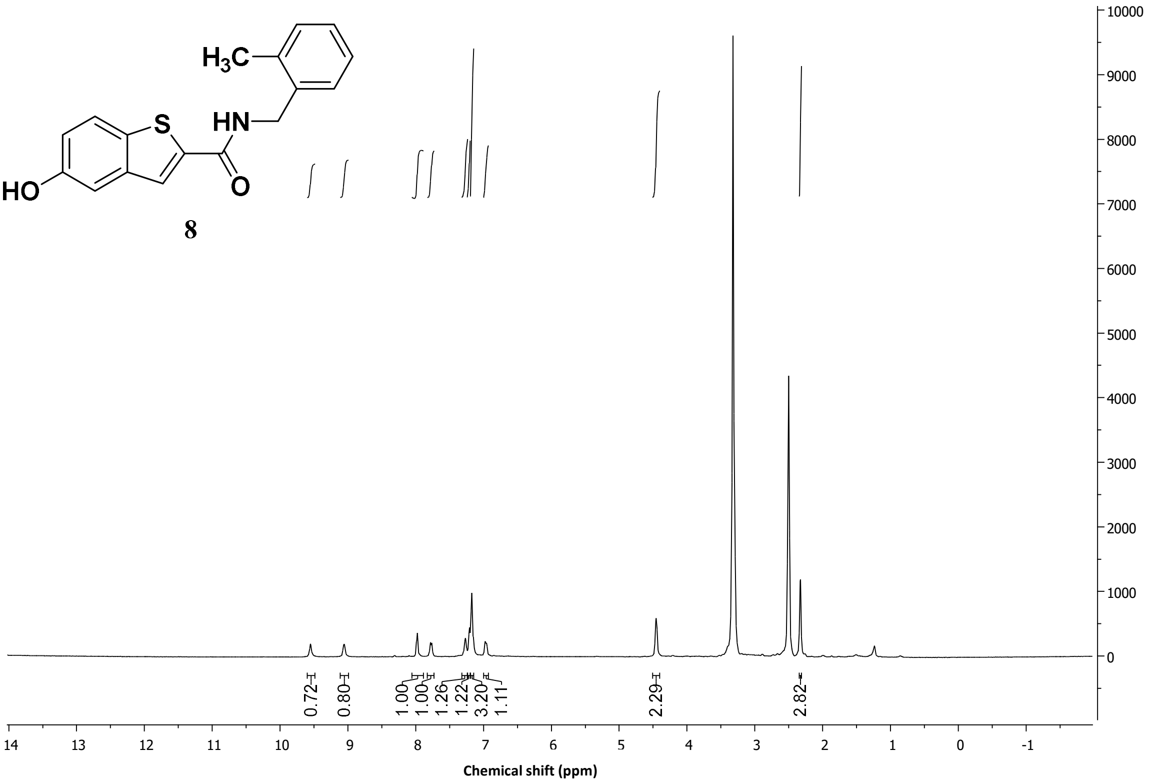

H3C
HN
S
HO
O
8
0.72
0.80
1.00
1.00
1.26
1.22
3.20
1.11
2.29
2.82
Chemical shift (ppm)

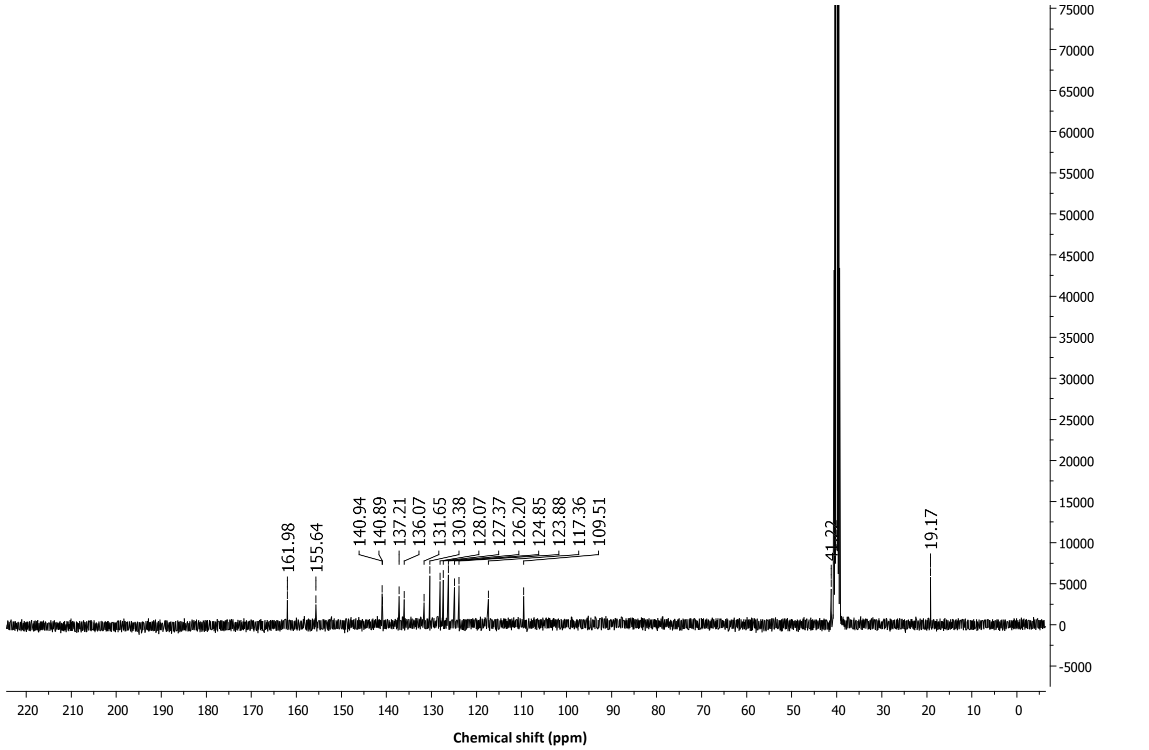

161.98
155.64
140.94
140.89
137.21
136.07
131.65
130.38
128.07
127.37
126.20
124.85
123.88
117.36
109.51
19.17
Chemical shift (ppm)

Compound 8

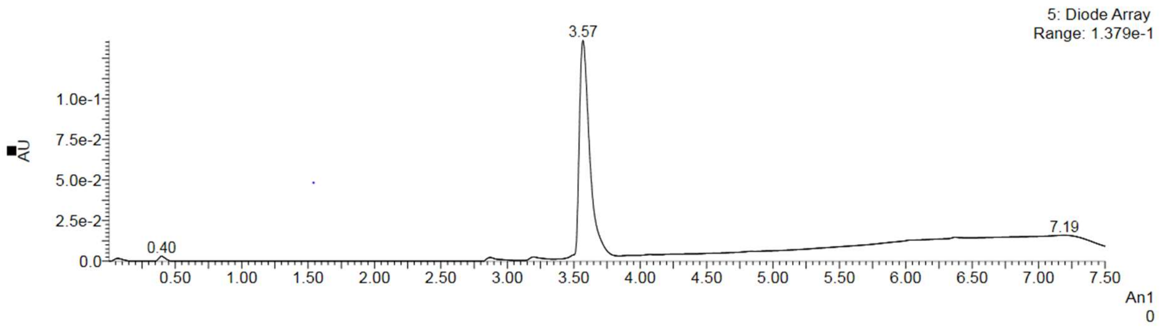
5: Diode Array
Range: 1.379e-1
3.57
7.19
0.40
AU
An1
0

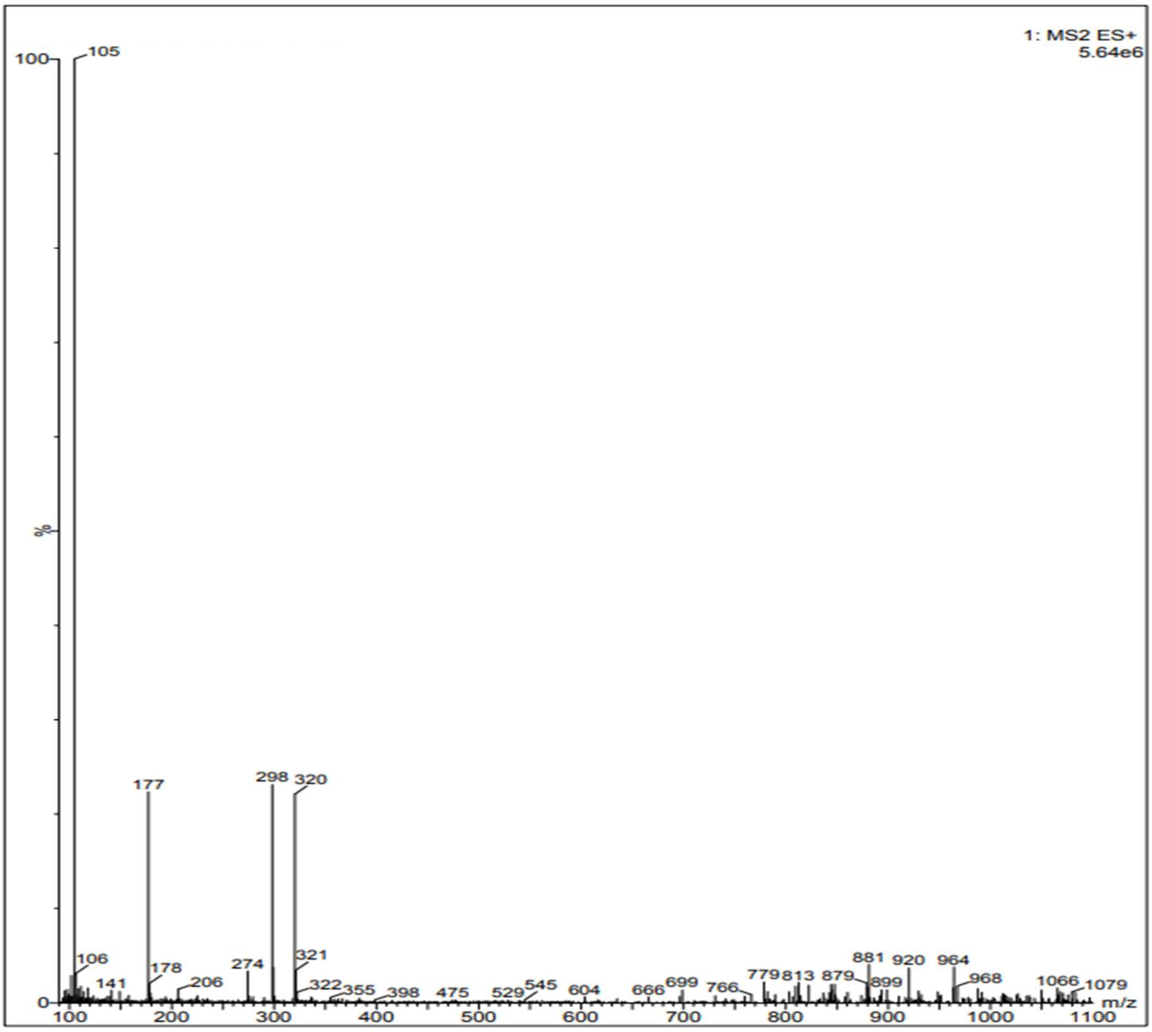
1: MS2 ES+
5.64e6
105
177
298
320
321
274
106
178
141
206
322
355
398
475
529
545
604
666
699
766
779
813
879
881
899
920
964
968
1066
1079
%
m/z

Compound 10

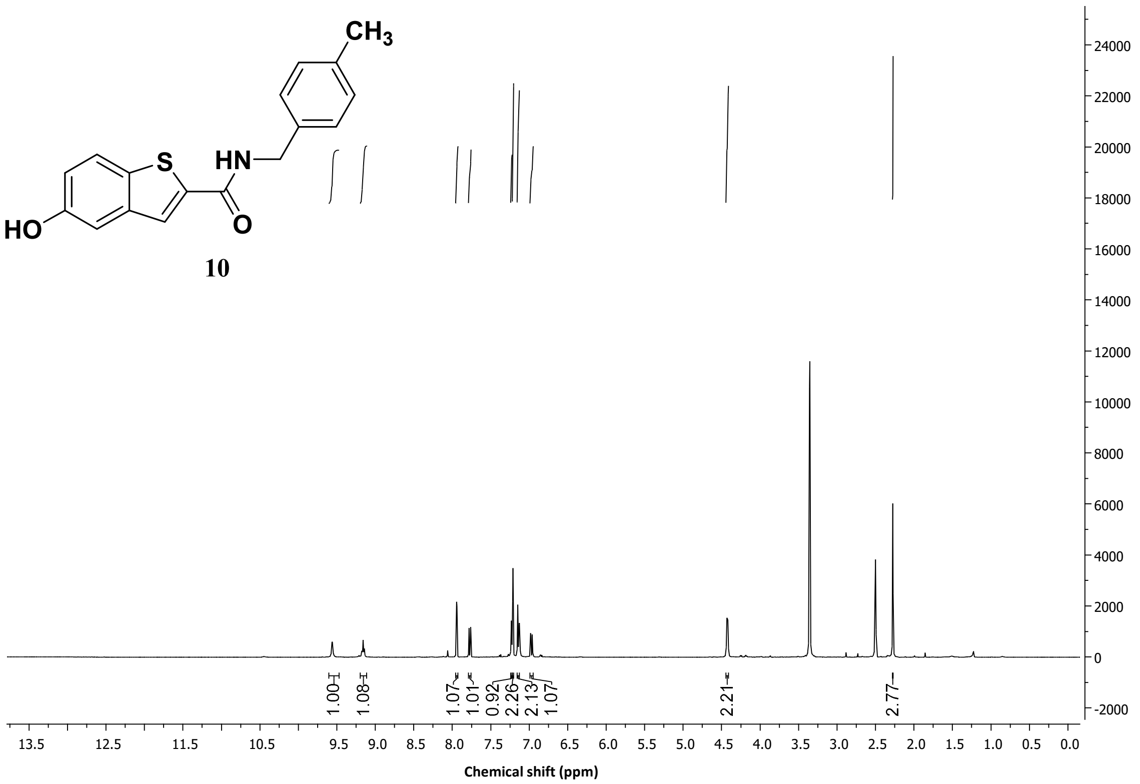

CH3
S
HN
HO
O
10
1.00
1.08
1.07
1.01
0.92
2.26
2.13
1.07
2.21
2.77
Chemical shift (ppm)

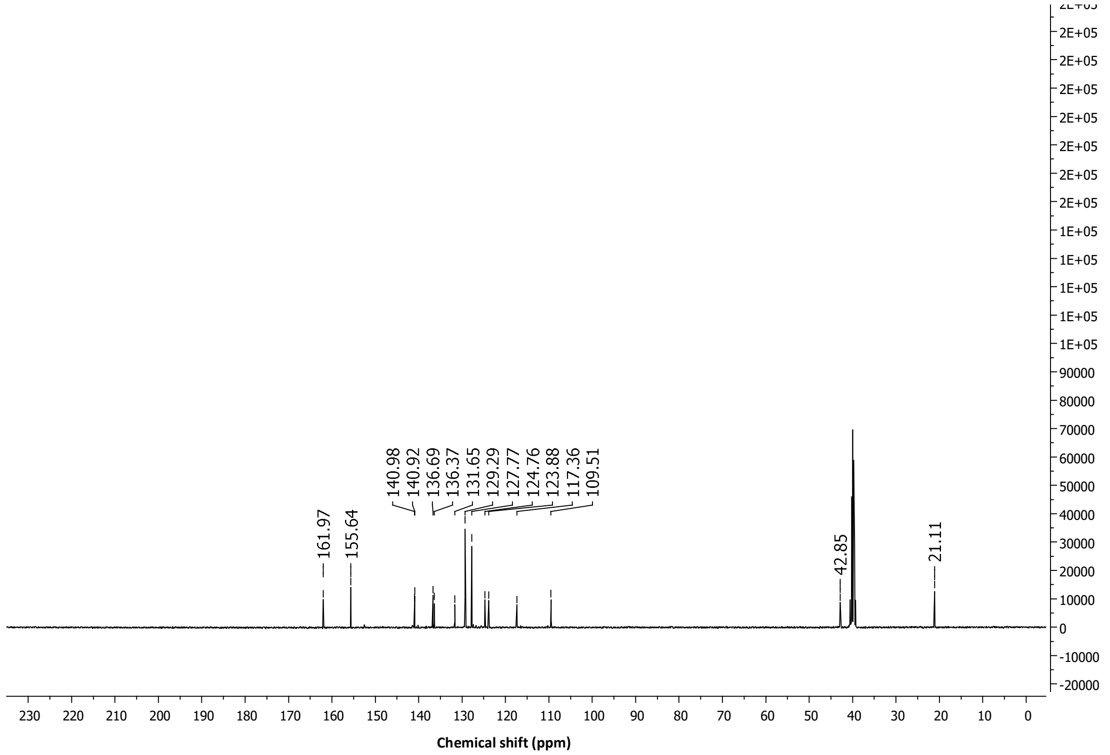

161.97
155.64
140.98
140.92
136.69
136.37
131.65
129.29
127.77
124.76
123.88
117.36
109.51
42.85
21.11
Chemical shift (ppm)

Compound 10

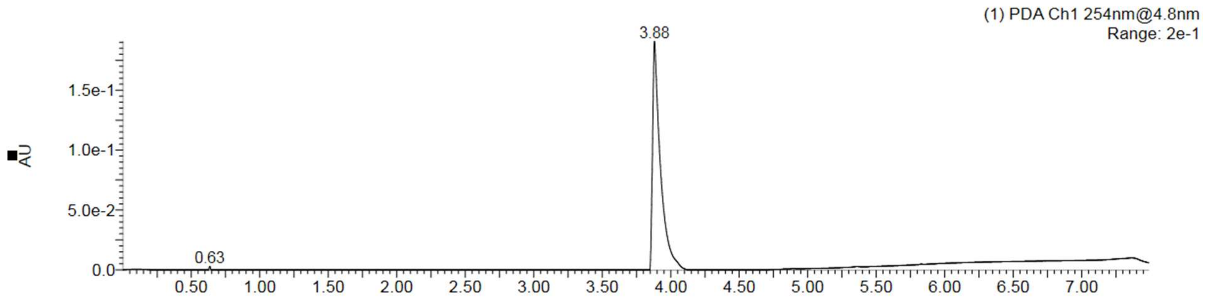
(1) PDA Ch1 254nm@4.8nm
Range: 2e-1
3.88
0.63
AU
1.5e-1
1.0e-1
5.0e-2
0.0
0.50 1.00 1.50 2.00 2.50 3.00 3.50 4.00 4.50 5.00 5.50 6.00 6.50 7.00

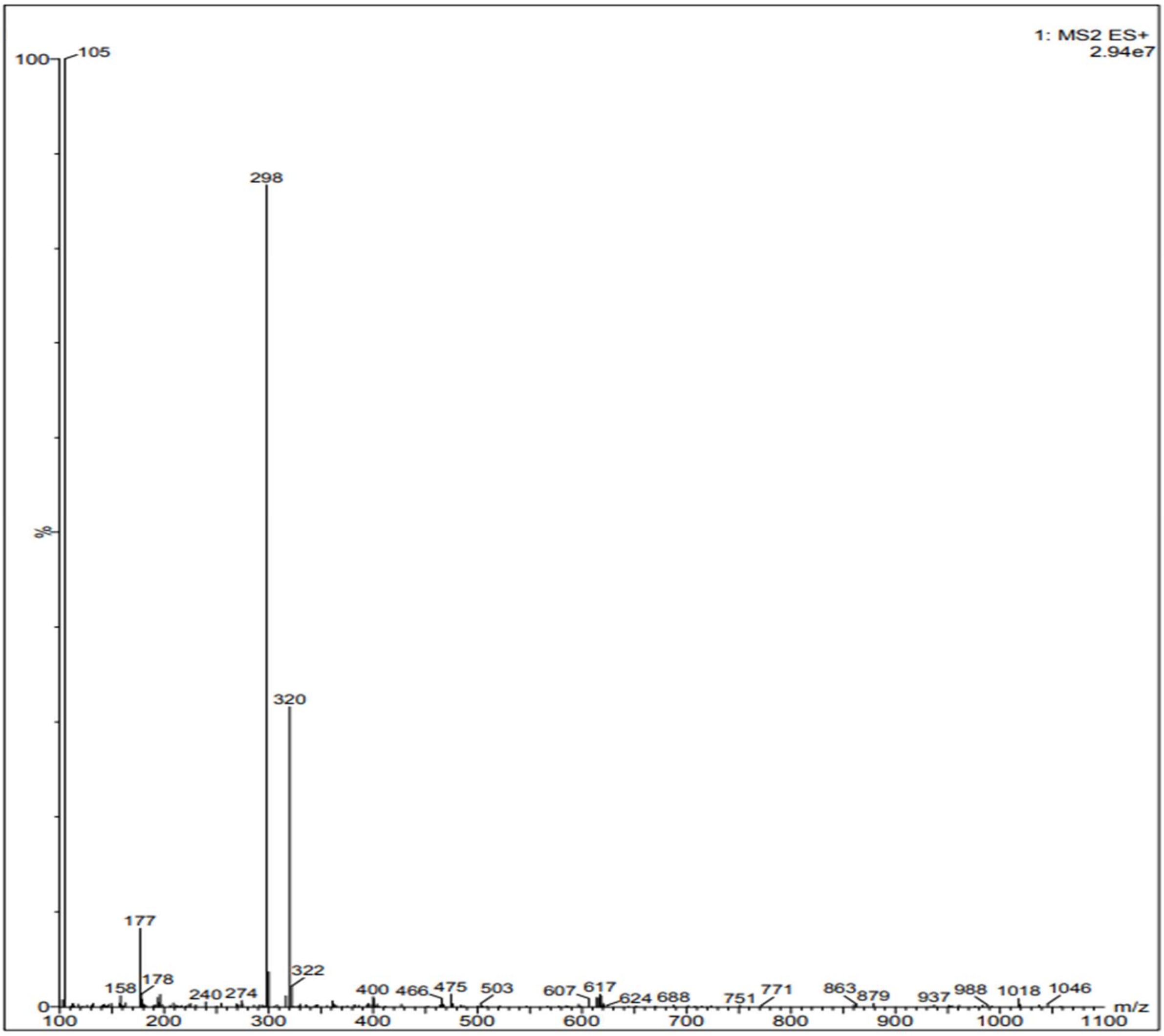
1: MS2 ES+
2.94e7
100
105
298
%
320
177
322
158 178 240 274 400 466 475 503 607 617 624 688 751 771 863 879 937 988 1018 1046
0
100 200 300 400 500 600 700 800 900 1000 1100
m/z

Compound 12

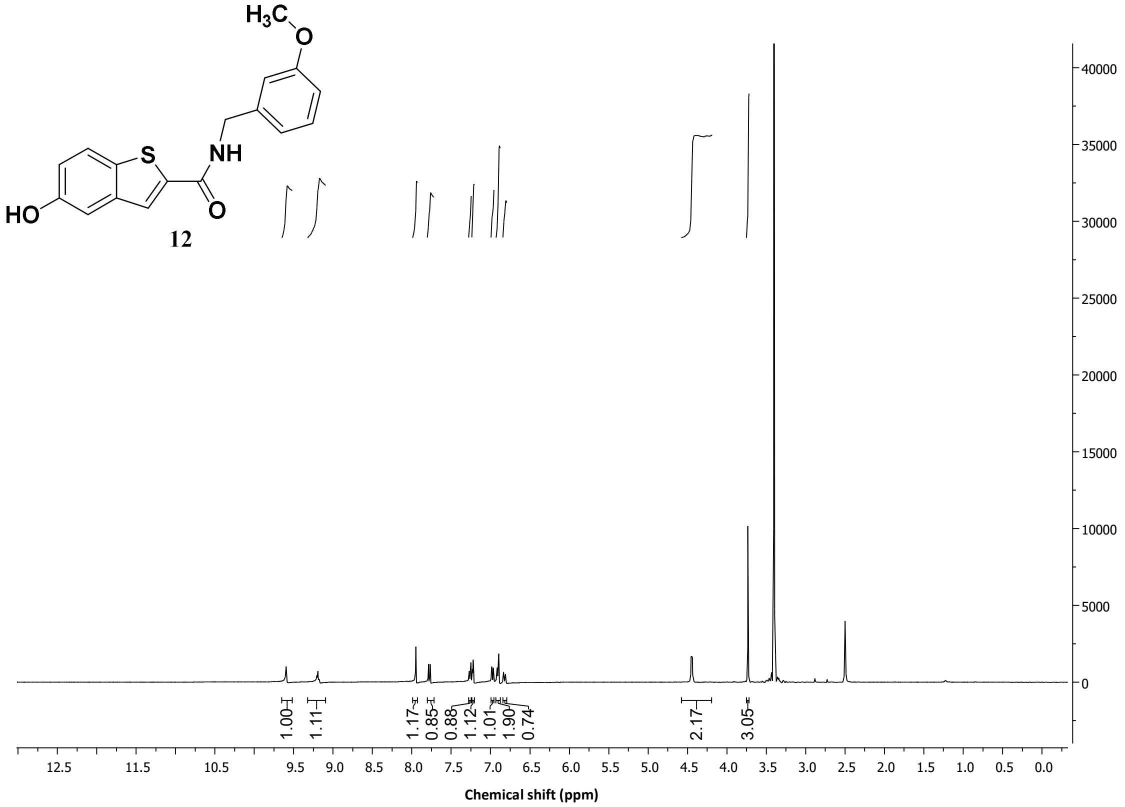
H3C
O
NH
S
O
HO
12
1.00
1.11
1.17
0.85
0.88
1.12
1.01
1.90
0.74
2.17
3.05
Chemical shift (ppm)

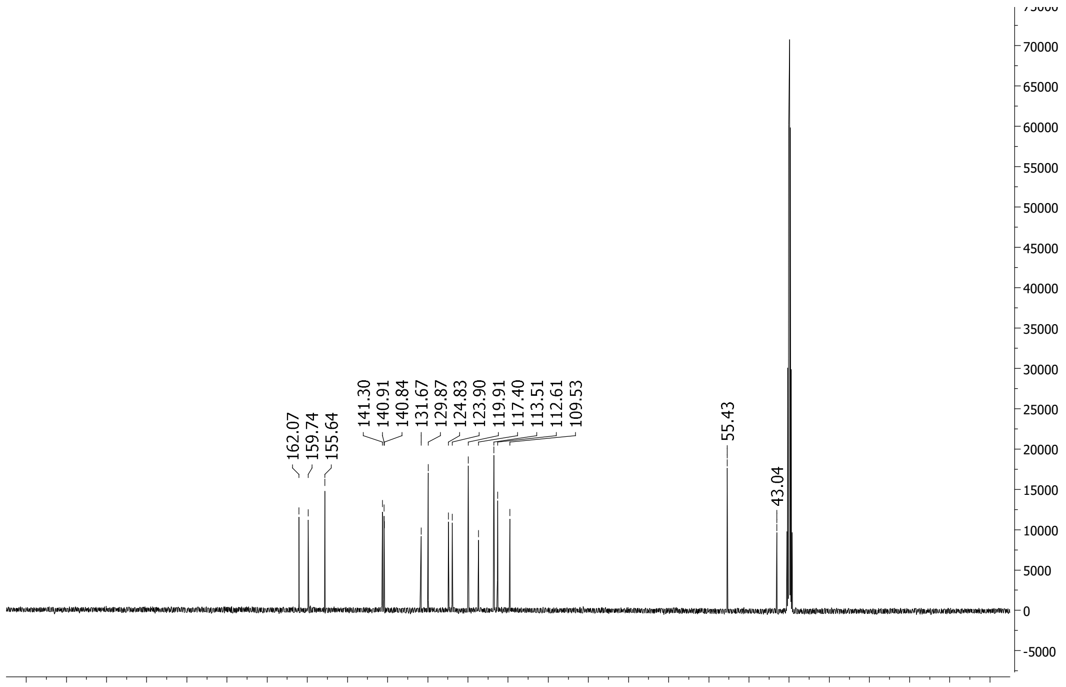
162.07
159.74
155.64
141.30
140.91
140.84
131.67
129.87
124.83
123.90
119.91
117.40
113.51
112.61
109.53
55.43
43.04
Chemical shift (ppm)

Compound 12

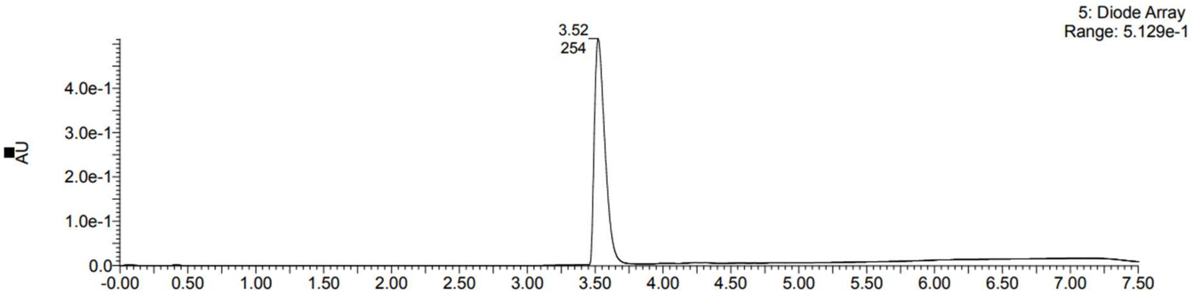

5: Diode Array
Range: 5.129e-1
3.52
254
AU
4.0e-1
3.0e-1
2.0e-1
1.0e-1
0.0
-0.00
0.50
1.00
1.50
2.00
2.50
3.00
3.50
4.00
4.50
5.00
5.50
6.00
6.50
7.00
7.50

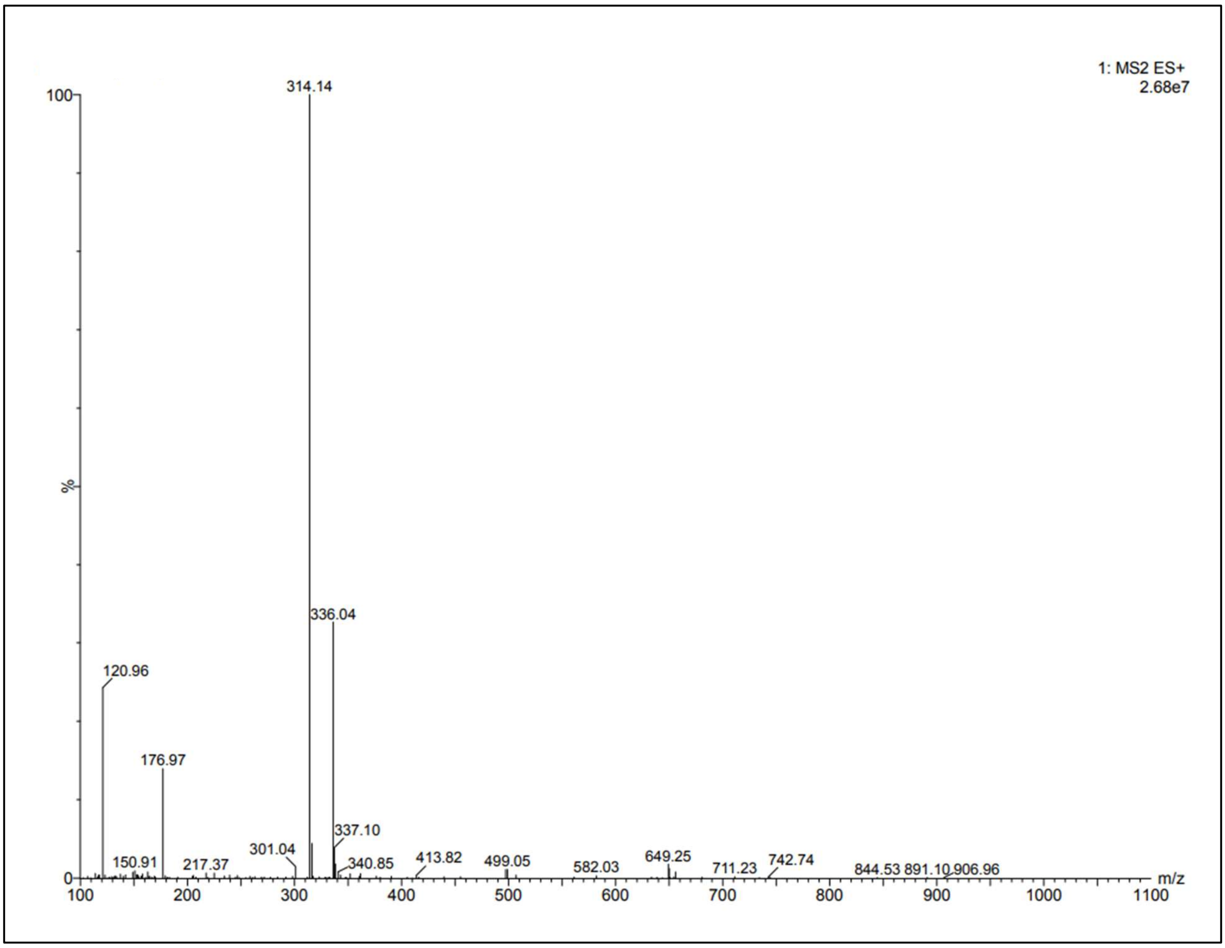

1: MS2 ES+
2.68e7
100
%
0
314.14
336.04
120.96
176.97
337.10
301.04
150.91
217.37
340.85
413.82
499.05
582.03
649.25
711.23
742.74
844.53 891.10 906.96
m/z
100
200
300
400
500
600
700
800
900
1000
1100

Compound 13

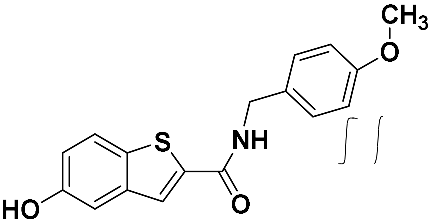

13

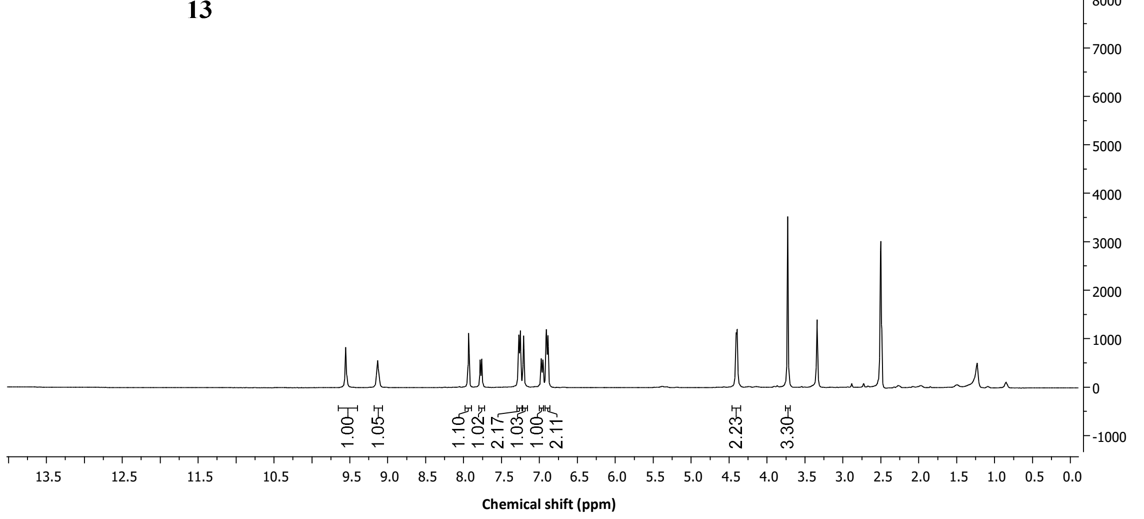

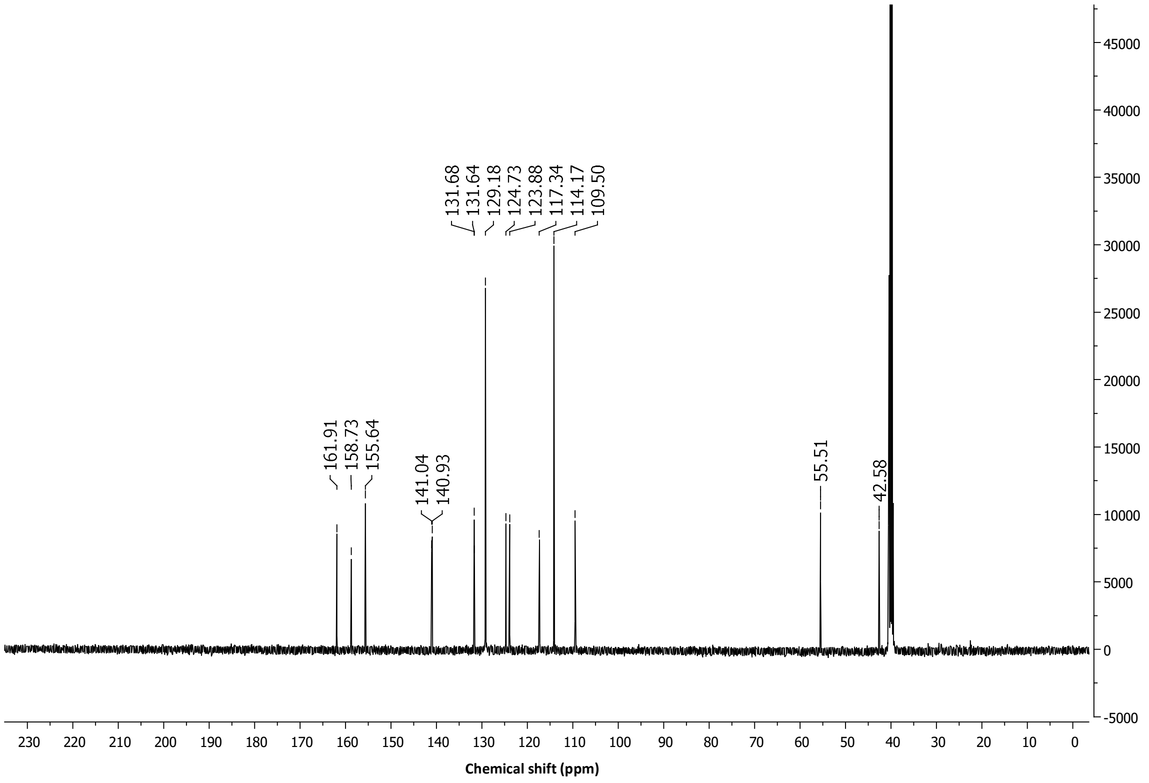

Compound 13

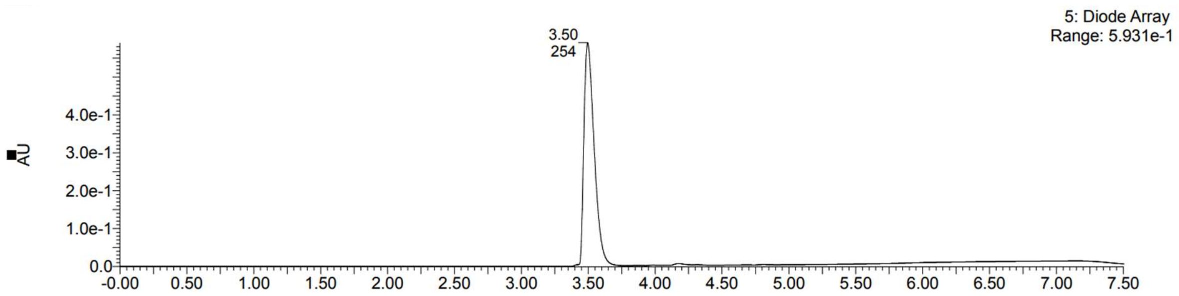

5: Diode Array
Range: 5.931e-1
3.50
254
AU
4.0e-1
3.0e-1
2.0e-1
1.0e-1
0.0
-0.00
0.50
1.00
1.50
2.00
2.50
3.00
3.50
4.00
4.50
5.00
5.50
6.00
6.50
7.00
7.50

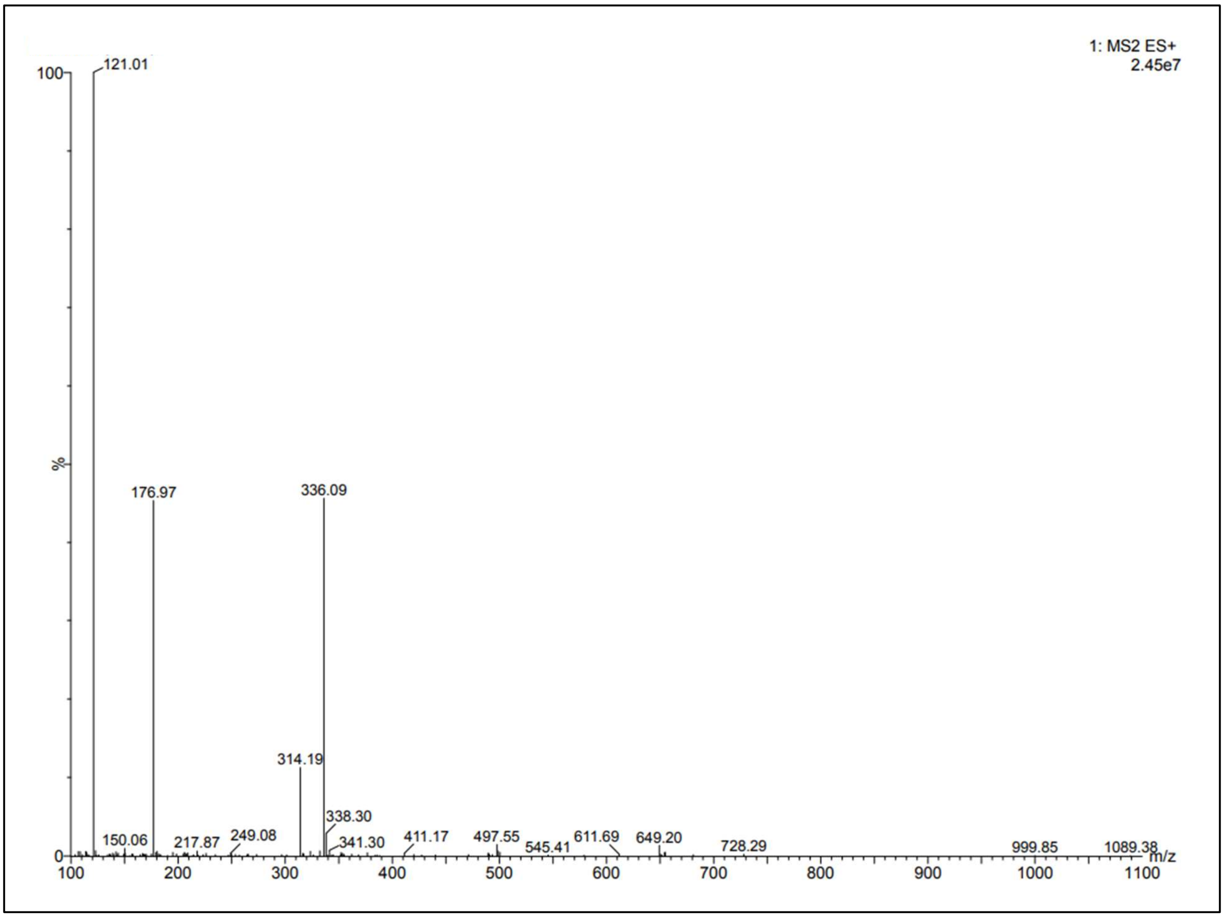

1: MS2 ES+
2.45e7
100
%
0
121.01
176.97
336.09
314.19
338.30
150.06
217.87
249.08
341.30
411.17
497.55
545.41
611.69
649.20
728.29
999.85
1089.38
m/z
100
200
300
400
500
600
700
800
900
1000
1100

Compound 17

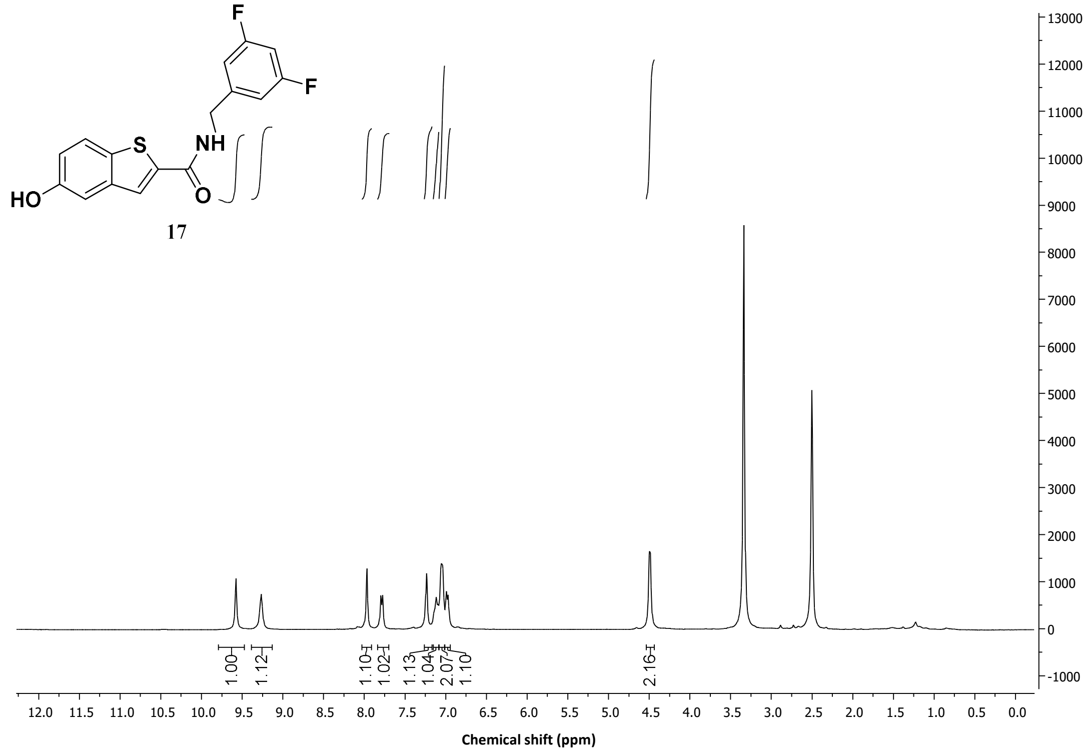

F
F
S
NH
HO
O
17
1.00
1.12
1.10
1.02
1.13
1.04
2.07
1.10
2.16
Chemical shift (ppm)

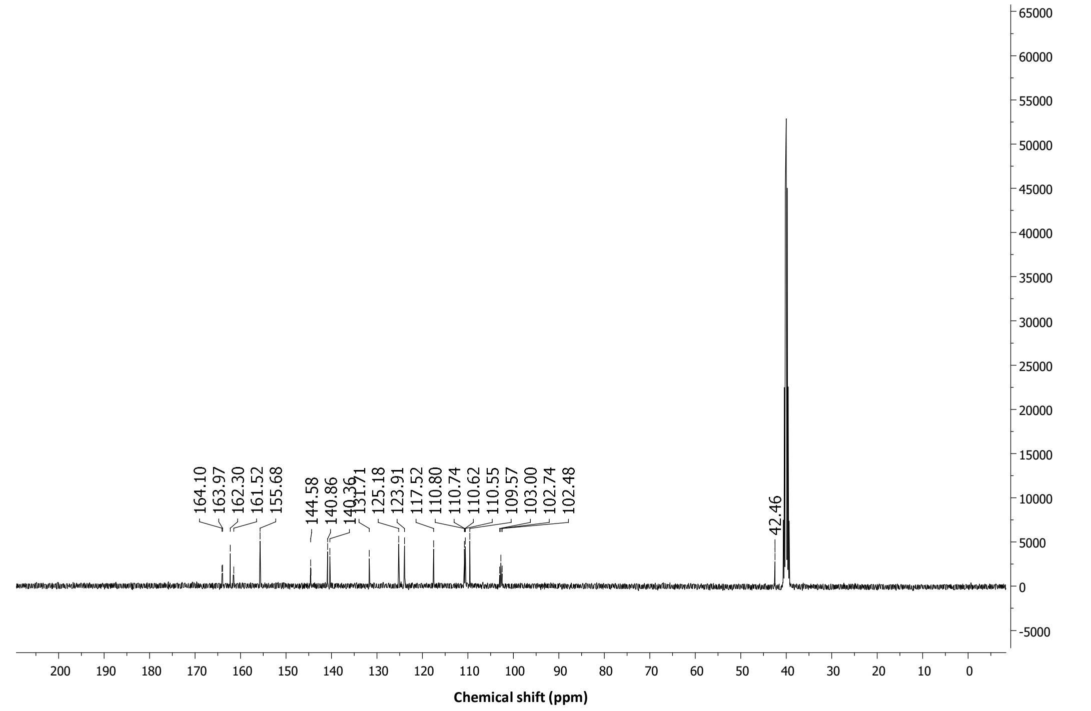

164.10
163.97
162.30
161.52
155.68
144.58
140.86
140.36
131.71
125.18
123.91
117.52
110.80
110.74
110.62
110.55
109.57
103.00
102.74
102.48
42.46
Chemical shift (ppm)

Compound 17

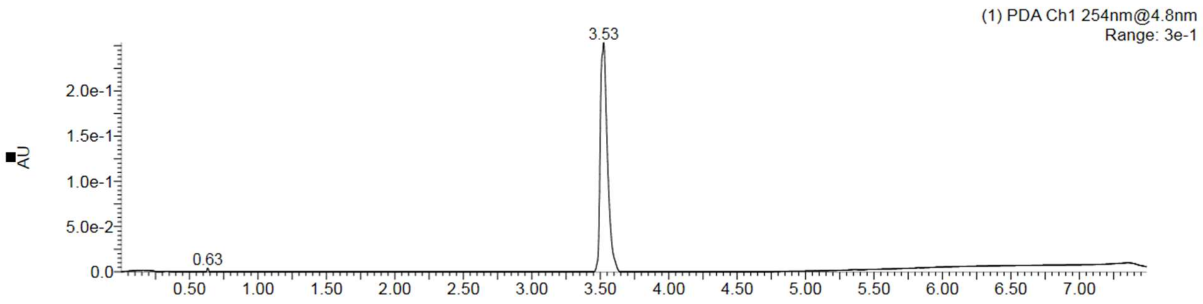
(1) PDA Ch1 254nm@4.8nm
Range: 3e-1
3.53
0.63
AU
2.0e-1
1.5e-1
1.0e-1
5.0e-2
0.0
0.50
1.00
1.50
2.00
2.50
3.00
3.50
4.00
4.50
5.00
5.50
6.00
6.50
7.00

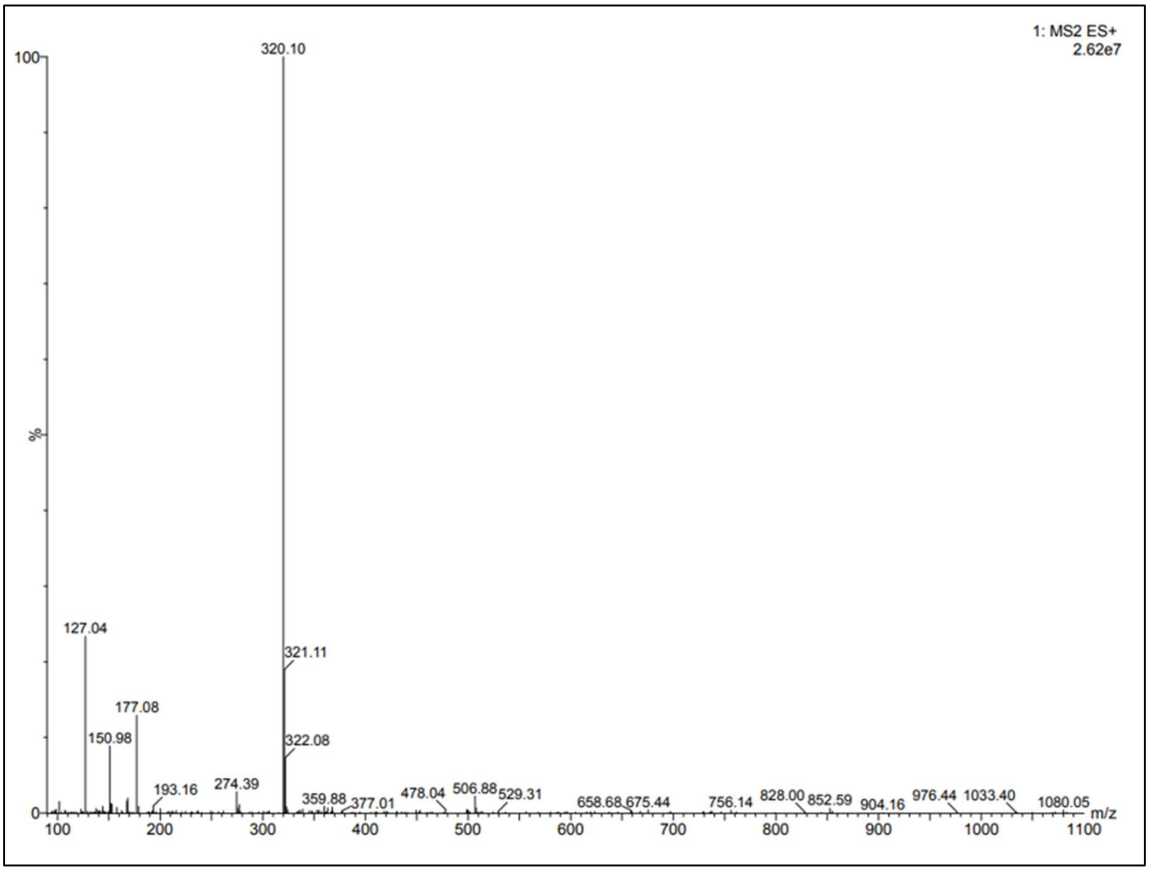
1: MS2 ES+
2.62e7
320.10
127.04
321.11
177.08
150.98
322.08
193.16
274.39
359.88
377.01
478.04
506.88
529.31
658.68
675.44
756.14
828.00
852.59
904.16
976.44
1033.40
1080.05
m/z
%
100
0
100
200
300
400
500
600
700
800
900
1000
1100

Compound 20

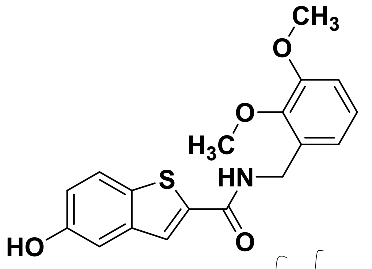

20

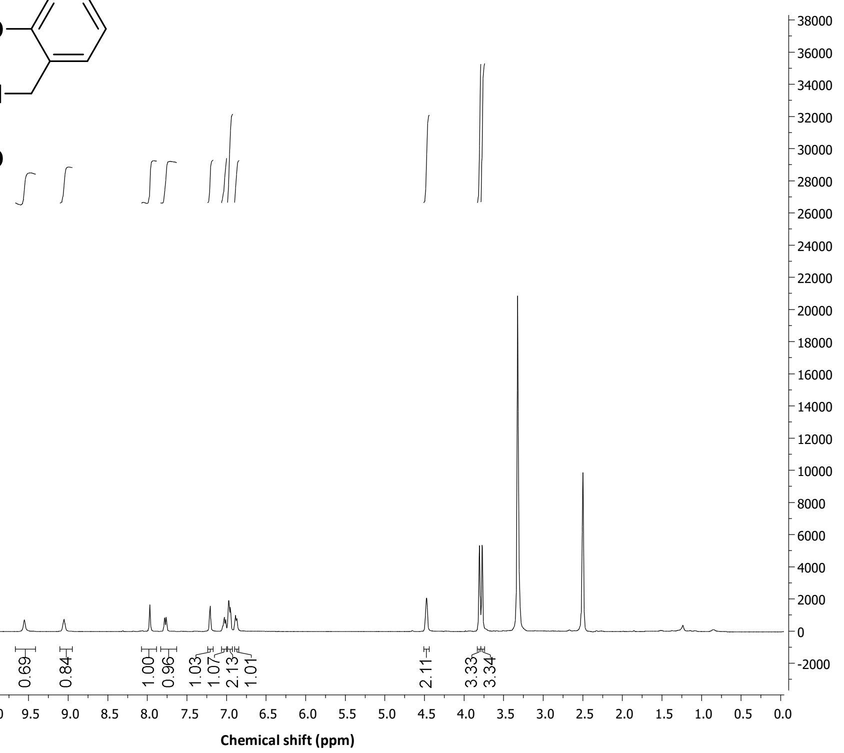

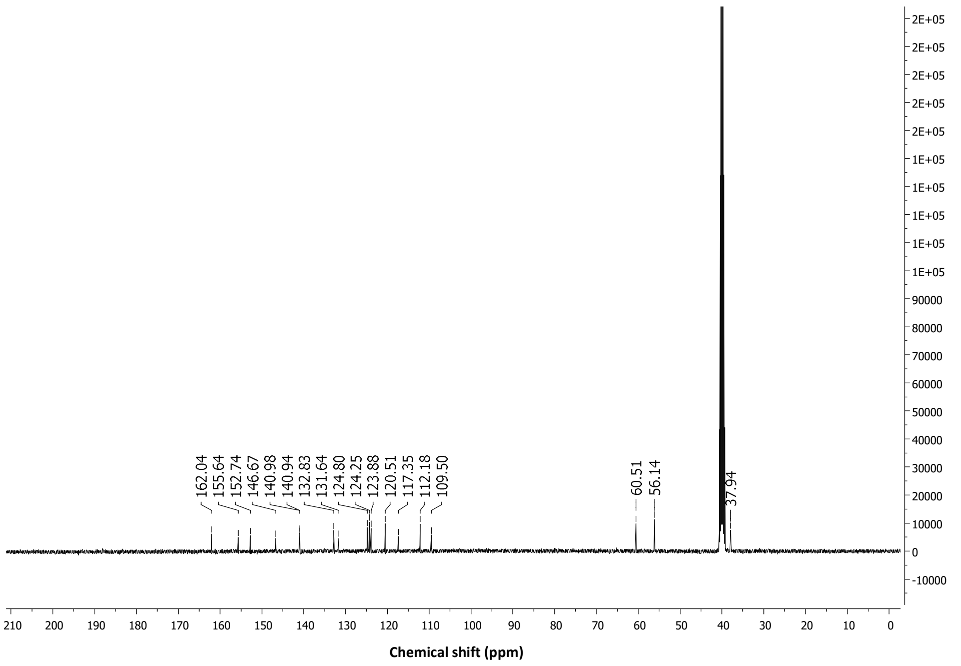

Compound 20

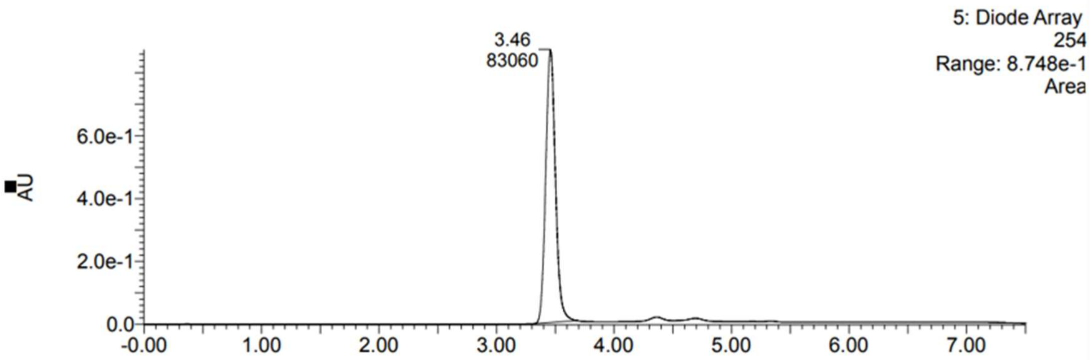

3.46
83060
5: Diode Array
254
Range: 8.748e-1
Area
AU
6.0e-1
4.0e-1
2.0e-1
0.0
-0.00
1.00
2.00
3.00
4.00
5.00
6.00
7.00

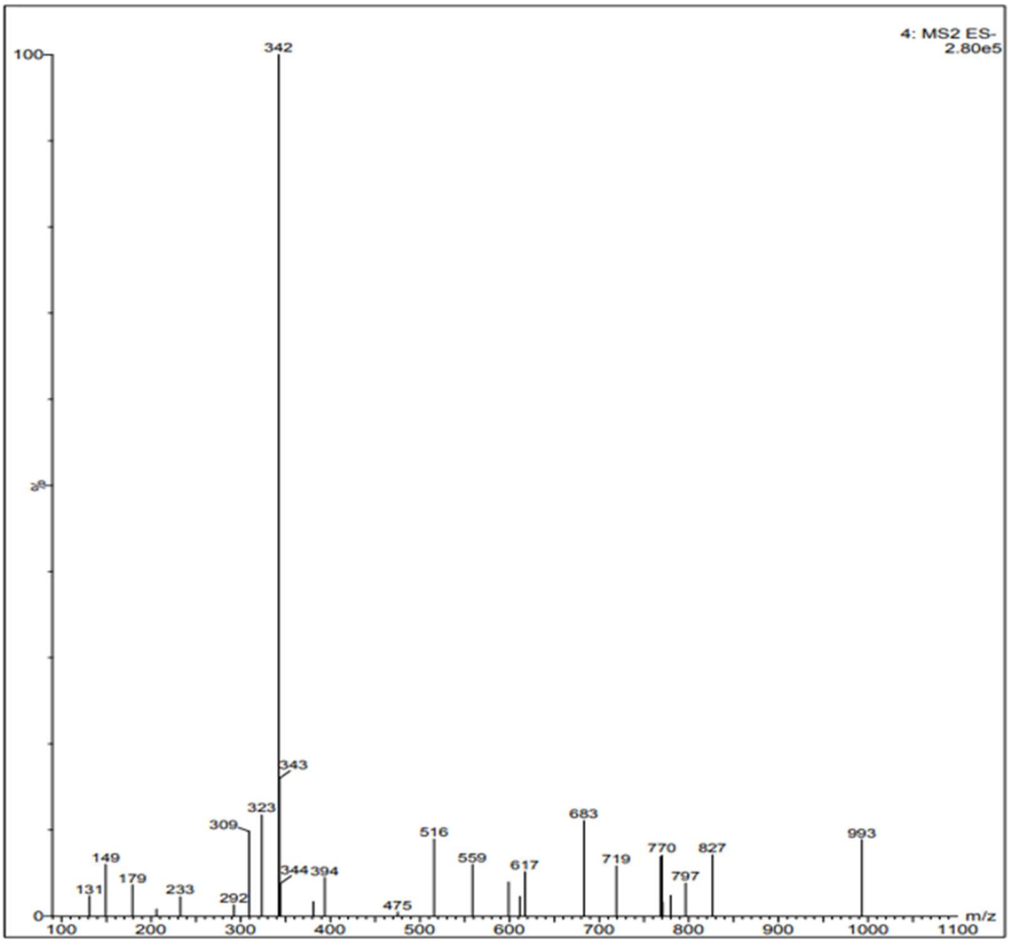

4: MS2 ES-
2.80e5
100
%
0
342
343
323
309
683
516
993
149
559
719
770
827
617
344
394
179
797
131
233
292
475
100
200
300
400
500
600
700
800
900
1000
1100
m/z

Compound 21

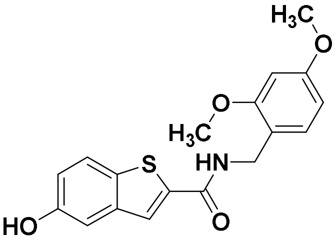

21

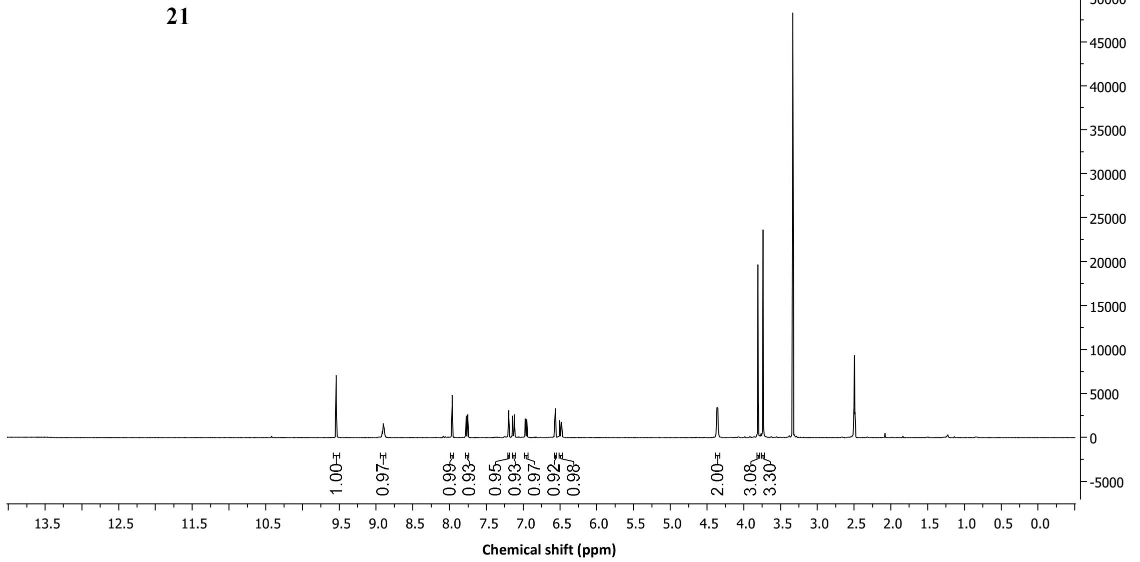

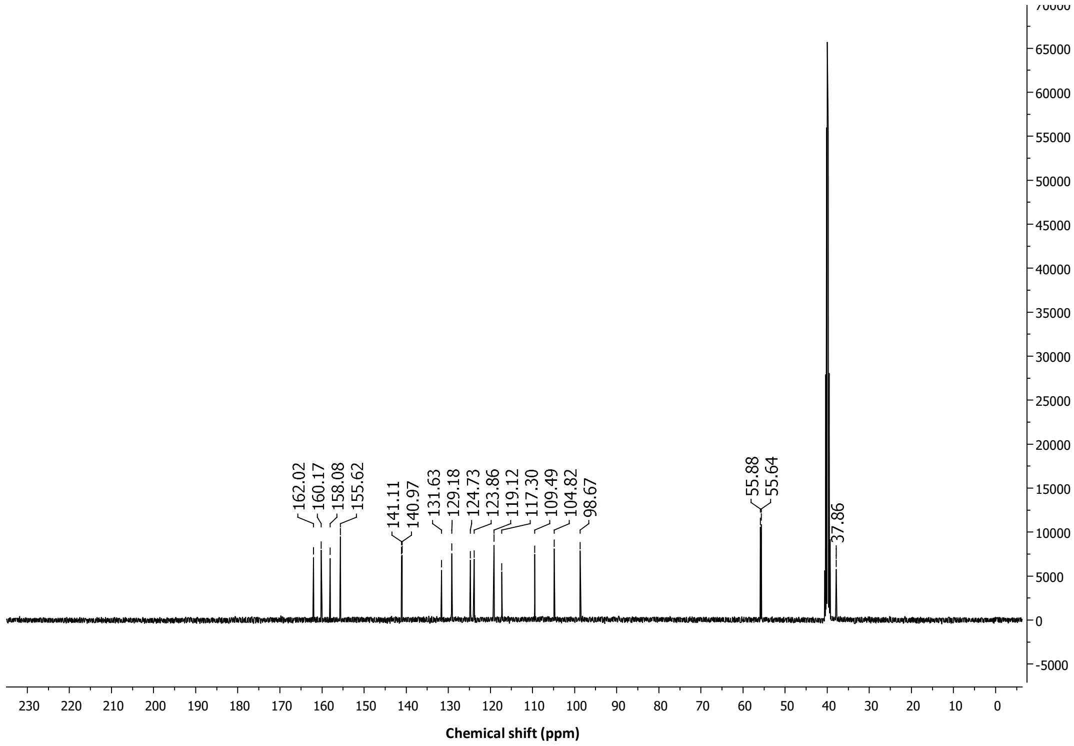

Compound 21

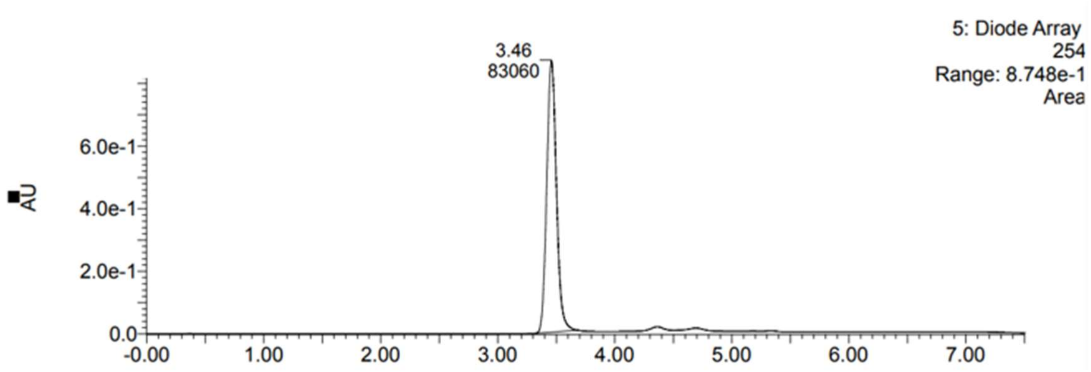
3.46
83060
5: Diode Array
254
Range: 8.748e-1
Area
AU
6.0e-1
4.0e-1
2.0e-1
0.0
-0.00
1.00
2.00
3.00
4.00
5.00
6.00
7.00

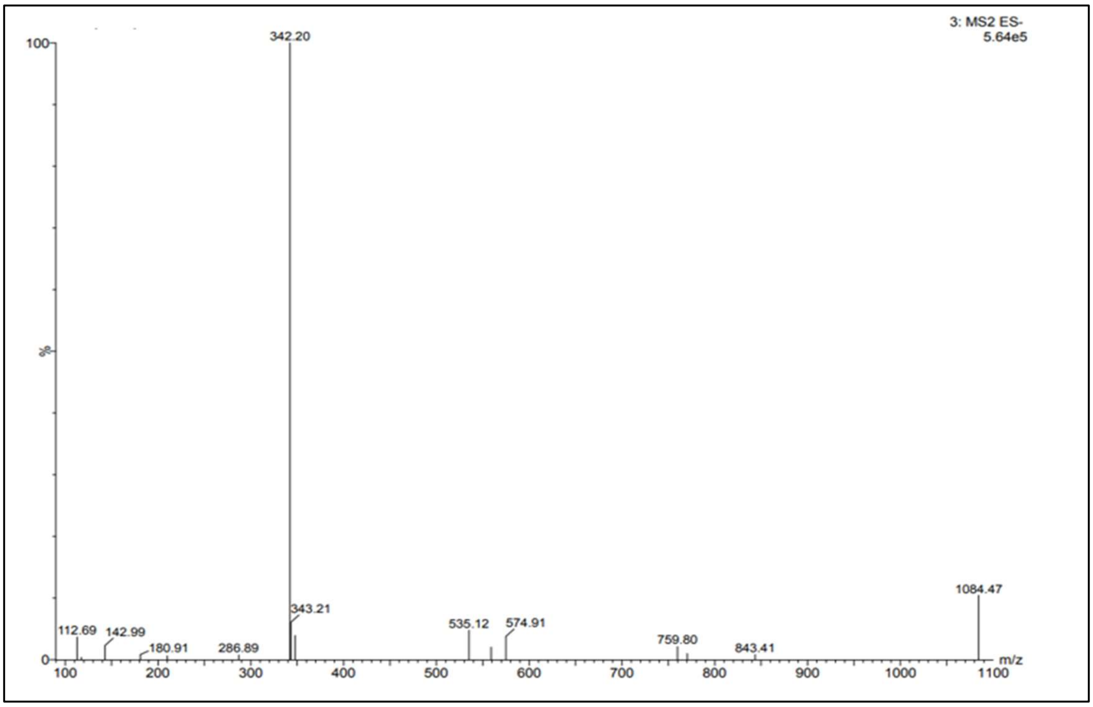
3: MS2 ES-
5.64e5
100
%
0
342.20
343.21
112.69
142.99
180.91
286.89
535.12
574.91
759.80
843.41
1084.47
m/z
100
200
300
400
500
600
700
800
900
1000
1100

3. Experimental procedure for protein kinases and inhibition assays.

3.1 The Z′-LYTE biochemical assay for screening and IC_{50} determination for Clk1, Dyrk1A, Dyrk1B, Clk2 and Clk3 kinases.

The assay employs a fluorescence-based, coupled-enzyme format and is based on the differential sensitivity of phosphorylated and non-phosphorylated peptides to proteolytic cleavage. The peptide substrate is labeled with two fluorophores-one at each end-that make up a FRET pair. In the primary reaction, the kinase transfers the gamma-phosphate of ATP to a single tyrosine, serine, or threonine residue in a synthetic FRET-peptide. In the secondary reaction, a site-specific protease recognizes and cleaves non-phosphorylated FRET-peptides. Phosphorylation of FRET-peptides suppresses cleavage by the Development Reagent. Cleavage disrupts FRET between the donor (i.e., coumarin) and acceptor (i.e., fluorescein) fluorophores on the FRET-peptide, whereas uncleaved, phosphorylated FRET-peptides maintain FRET. A ratiometric method, which calculates the ratio (the Emission Ratio) of donor emission to acceptor emission after excitation of the donor fluorophore at 400 nm, is used to quantitate reaction progress. Both cleaved and uncleaved FRET-peptides contribute to the fluorescence signals and therefore to the Emission Ratio. The extent of phosphorylation of the FRET-peptide can be calculated from the Emission Ratio. The Emission Ratio will remain low if the FRET-peptide is phosphorylated (i.e., no kinase inhibition) and will be high if the FRET-peptide is non-phosphorylated (i.e., kinase inhibition). The IC_{50} values were determined for compounds displaying significant % inhibition in the screening step. IC_{50} determination was done through testing at a range of concentrations with two replicates per concentration; the IC_{50} value recorded for each compound is an average of at least two independent experiments.

3.1.1 Clk1 kinase assay specific conditions:

The 2x Clk1 / Ser/Thr 09 mixture is prepared in 50 mM HEPES pH 7.5, 0.01% BRIJ-35, 10 mM $MgCl_2$, 1 mM EGTA. The final 10 µL kinase reaction consists of 30 - 120 ng Clk1 and 2 µM Ser/Thr 09 in 50 mM HEPES pH 7.5, 0.01% BRIJ-35, 10 mM $MgCl_2$, 1 mM EGTA. After the 1 h kinase reaction incubation, 5 µL of a 1:512 dilution of development Reagent A is added. Staurosporine (IC_{50} = 92.4 nM) was used as a positive control.

3.1.2 Dyrk1A kinase assay specific conditions:

The 2x Dyrk1A / Ser/Thr 18 mixture is prepared in 50 mM HEPES pH 7.5, 0.01% BRIJ-35, 10 mM $MgCl_2$, 1 mM EGTA. The final 10 µL kinase reaction consists of 0.77 - 3.08 ng Dyrk1A and 2 µM Ser/Thr 18 in 50 mM HEPES pH 7.5, 0.01% BRIJ-35, 10 mM $MgCl_2$, 1 mM EGTA. After the 1 h kinase reaction incubation, 5 µL of a 1:1024 dilution of Development Reagent A is added. Staurosporine (IC_{50} = 26.5 nM) was used as a positive control.

3.1.3 Dyrk1B kinase assay specific conditions:

The 2X Dyrk1B / Ser/Thr 18 mixture is prepared in 50 mM HEPES pH 6.5, 0.01% BRIJ-35, 10 mM MgCl2, 1 mM EGTA, 0.02% NaN3. The final 10 µL kinase reaction consists of 0.59 - 2.37 ng Dyrk1B and 2 µM Ser/Thr 18 in 50 mM HEPES pH 7.0, 0.01% BRIJ-35, 10 mM MgCl2, 1 mM EGTA, 0.01% NaN3. After the 1 hour Kinase Reaction incubation, 5 µL of a 1:1024 dilution of Development Reagent A is added. Staurosporine (IC_{50} = 5.85 nM) was used as a positive control.

3.1.4 Clk2 kinase assay specific conditions:

The 2X Clk2 / Ser/Thr 06 mixture is prepared in 50 mM HEPES pH 7.5, 0.01% BRIJ-35, 10 mM MgCl2, 1 mM EGTA. The final 10 µL kinase reaction consists of 0.97 - 10.4 ng CLK2 and 2 µM Ser/Thr 06 in 50 mM HEPES pH 7.5, 0.01% BRIJ-35, 10 mM MgCl2, 1 mM EGTA. After the 1 hour Kinase Reaction incubation, 5 µL of a 1:4096 dilution of Development Reagent A is added. Staurosporine (IC_{50} = 9.31 nM) was used as a positive control

3.1.5 Clk3 kinase assay specific conditions:

The 2X Clk3 / Ser/Thr 18 mixture is prepared in 50 mM Tris pH 8.5, 0.01% BRIJ-35, 10 mM MgCl2, 1 mM EGTA, 0.02% NaN3. The final 10 µL Kinase Reaction consists of 9.57 - 38.3 ng CLK3 and 2 µM Ser/Thr 18 in 50 mM Tris / HEPES pH 8.0, 0.01% BRIJ-35, 10 mM MgCl2, 1 mM EGTA, 0.01% NaN3. After the 1 hour Kinase Reaction incubation, 5 µL of a 1:1024 dilution of Development Reagent A is added. Staurosporine (IC_{50} = 1160 nM) was used as a positive control.

3.2 The Adapta universal kinase assay for screening and IC_{50} determination against haspin. The haspin kinase assay was done at Thermo Fisher Scientific, Madison, WI, USA using a fluorescence-based immunoassay known as 'The Adapta Universal Kinase Assay', which detects the ADP produced by kinases utilizing the TR-FRET technology. The final 10 μL kinase reaction consists of 0.25 - 1 ng GSG2 (Haspin) and 100 μM of the substrate (Histone H3 (1-20) peptide) in 32.5 mM HEPES pH 7.5, 0.005% BRIJ-35, 5 mM $MgCl_2$, 0.5 mM EGTA, in the presence of 25 μM ATP. After an incubation time of 1 h at RT, 5 μL of a detection mix was added. Staurosporine (IC_{50} = 7.7 nM) was used as a positive control. To determine the half maximal inhibitory concentration (IC_{50}), the assays were performed in duplicate in the absence or presence of increasing doses of the tested compounds.

4. Dose response curves.

IC_{50} against Clk1

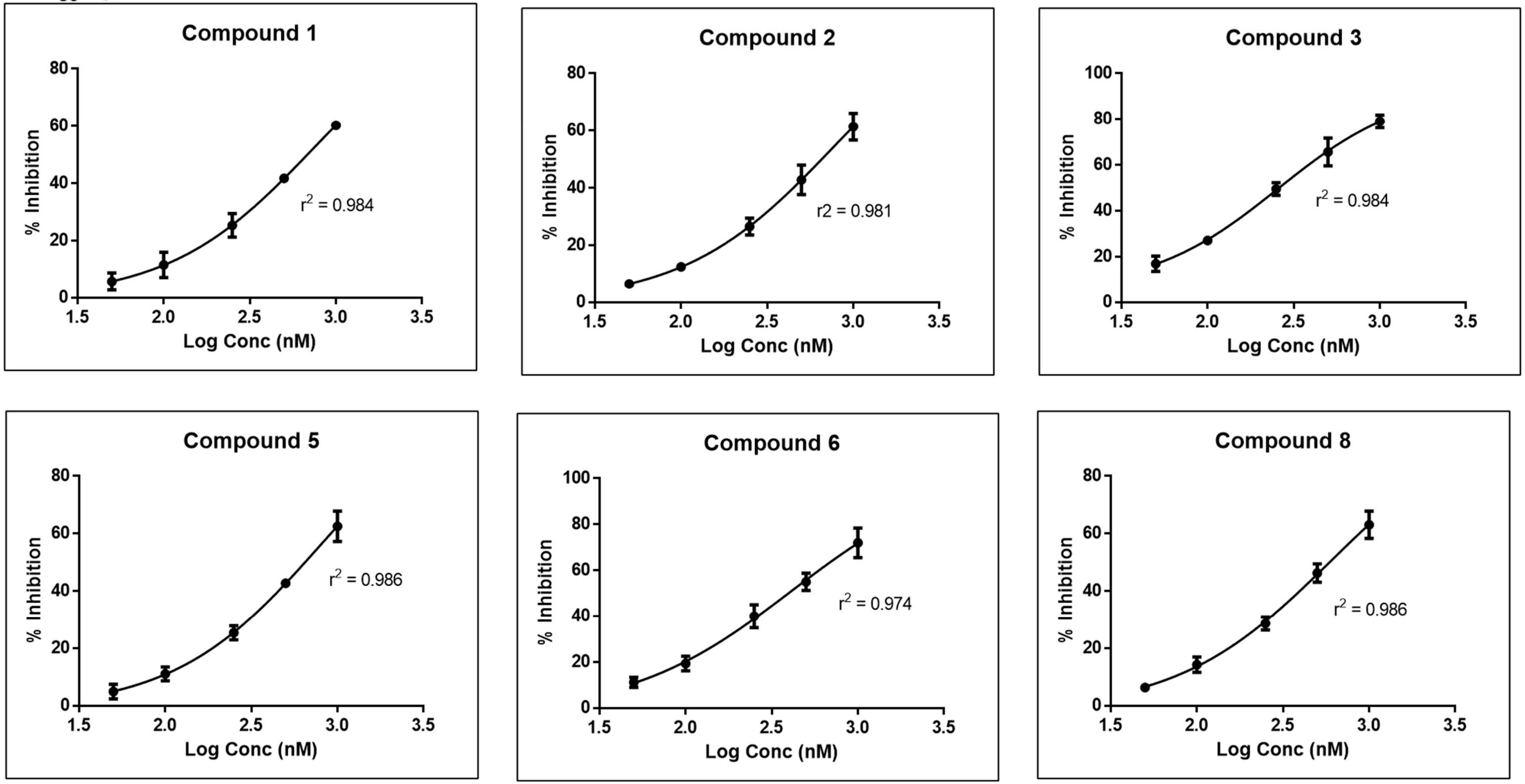

IC_{50} against Clk1

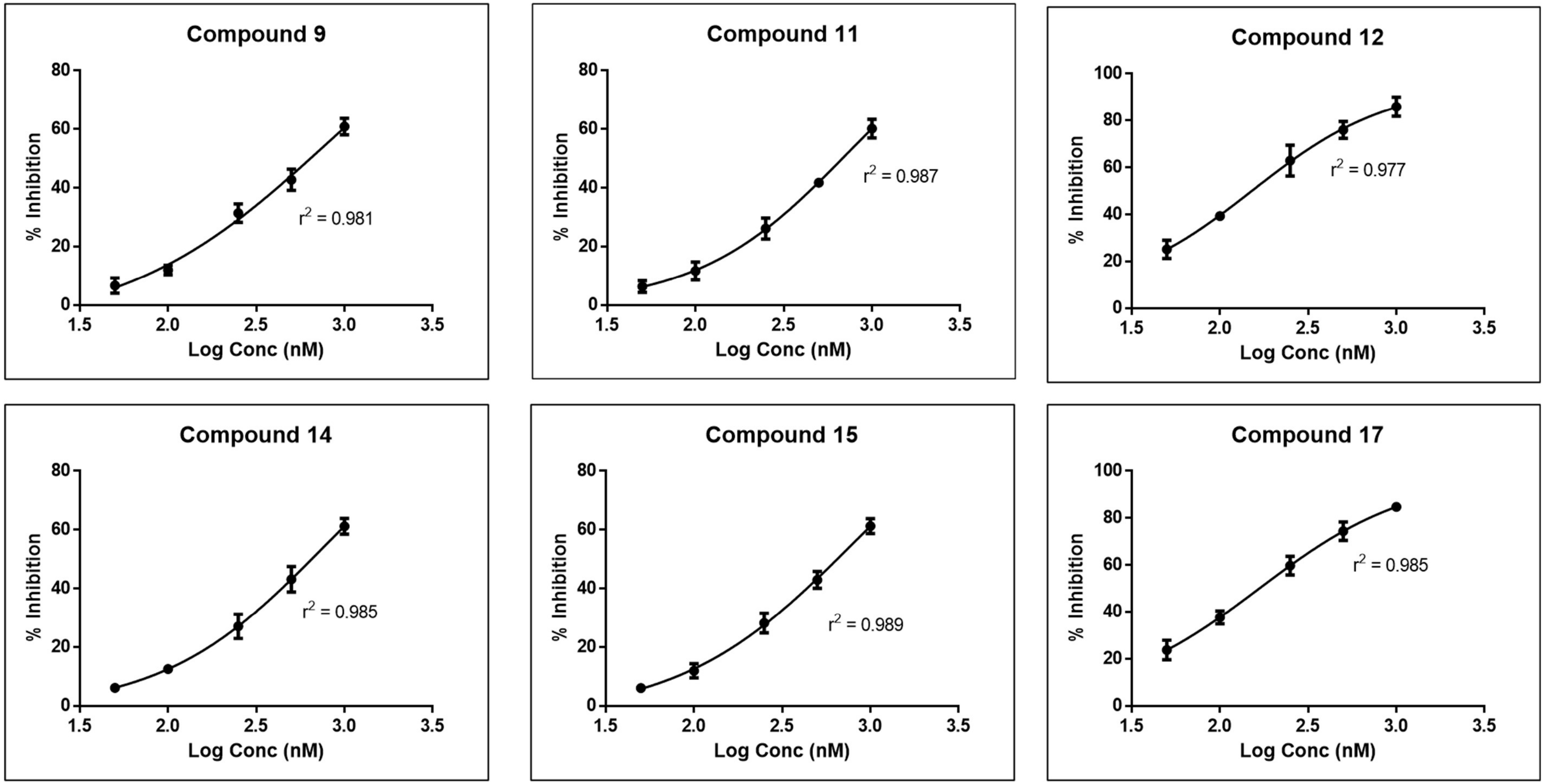

IC_{50} against Clk1

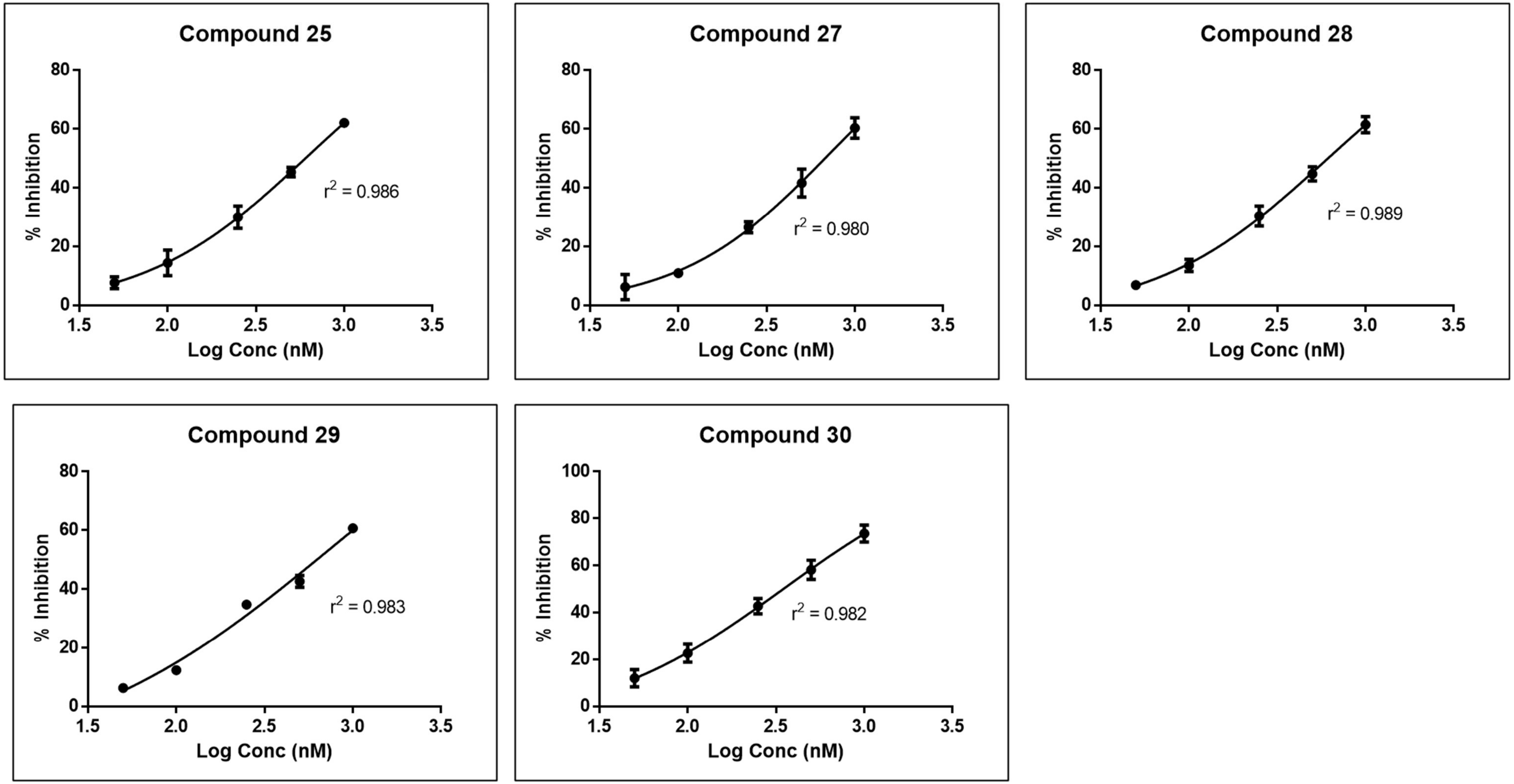

IC_{50} against Dyrk1A

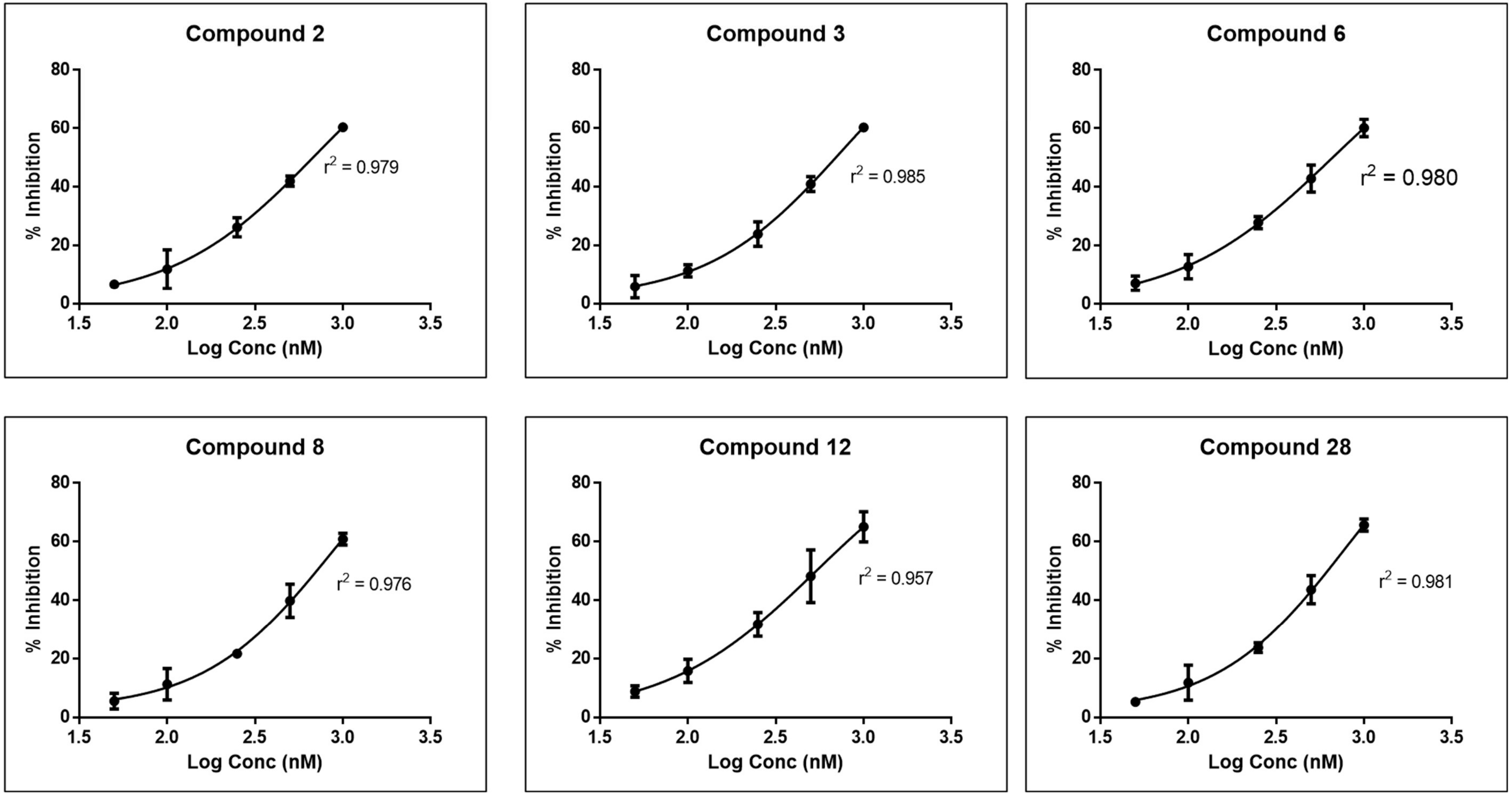

IC_{50} against Dyrk1A

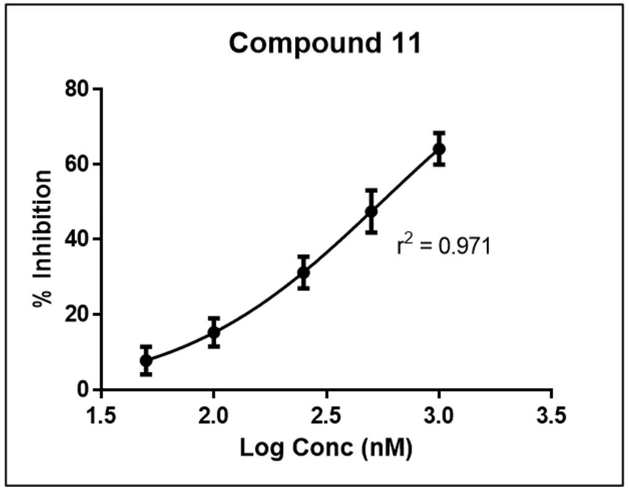

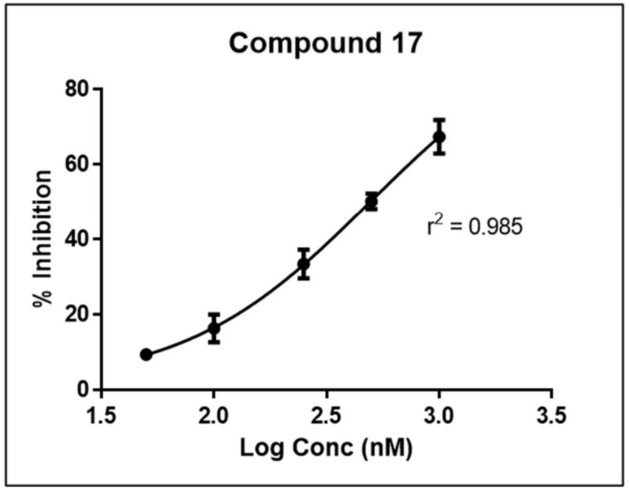

IC_{50} against Dyrk1B

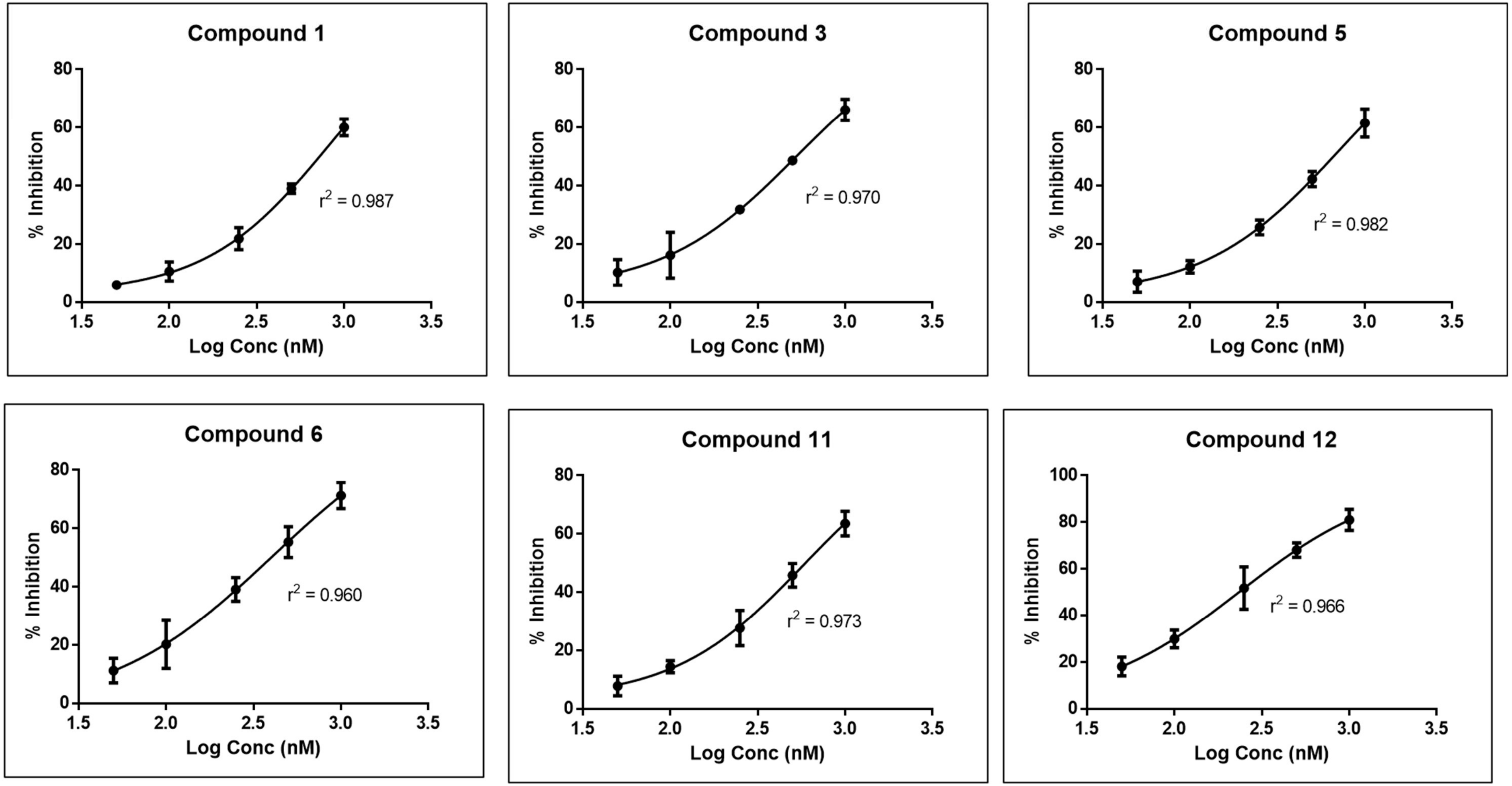

IC_{50} against Dyrk1B

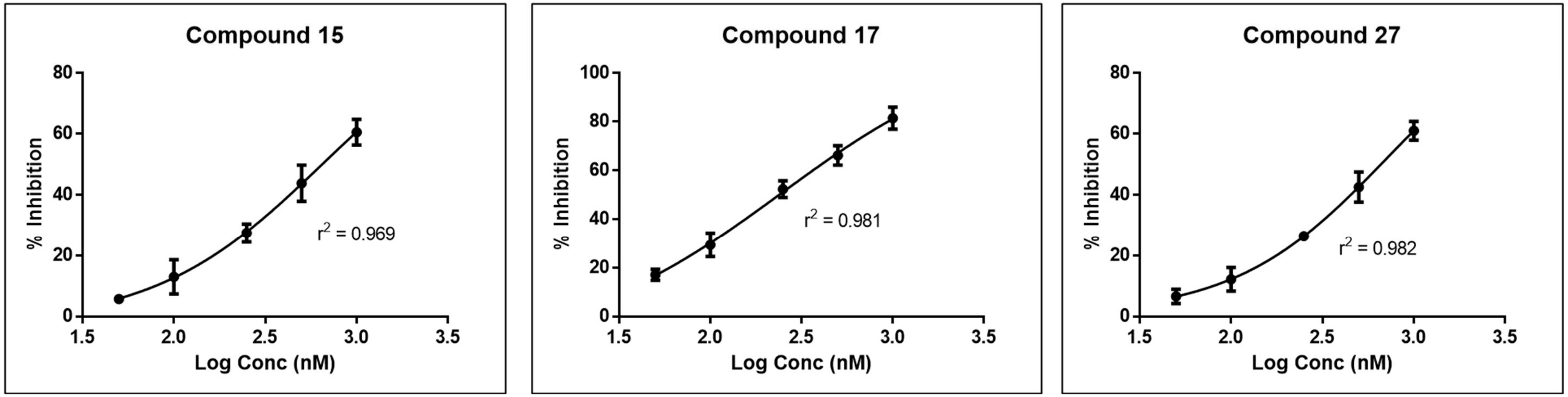

IC_{50} against Haspin

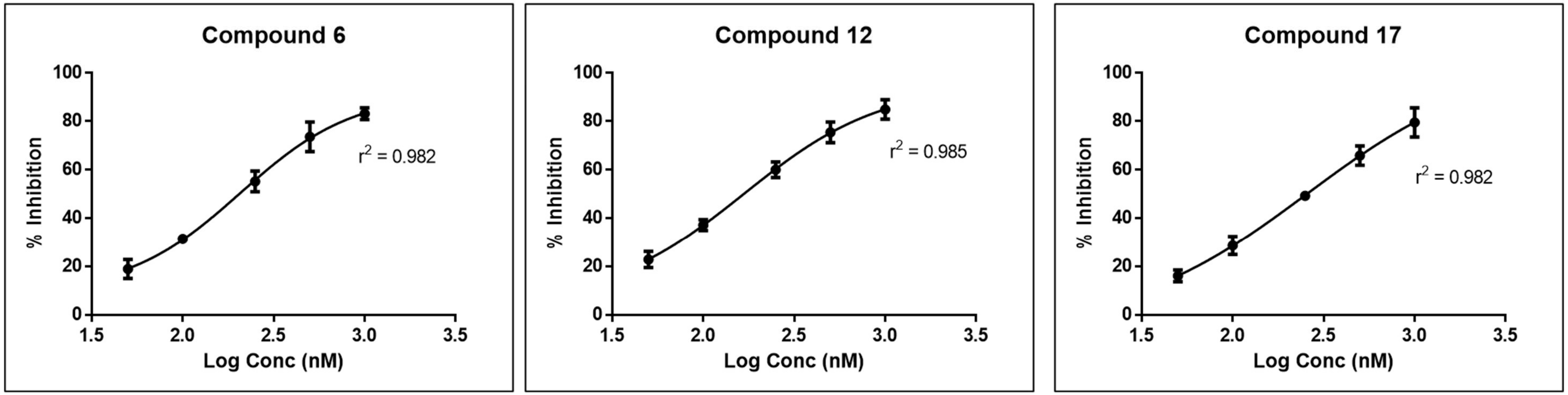

IC$_{50}$ against Clk2

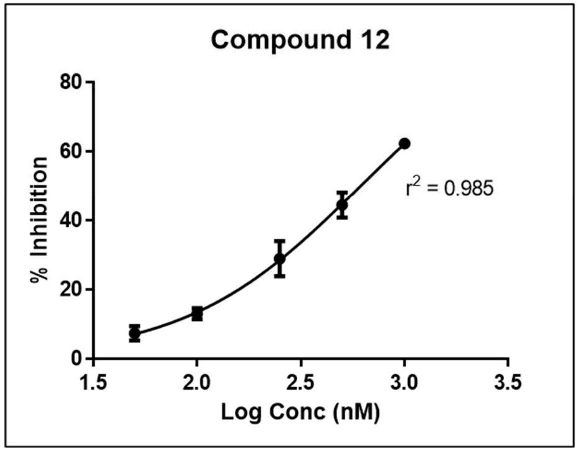

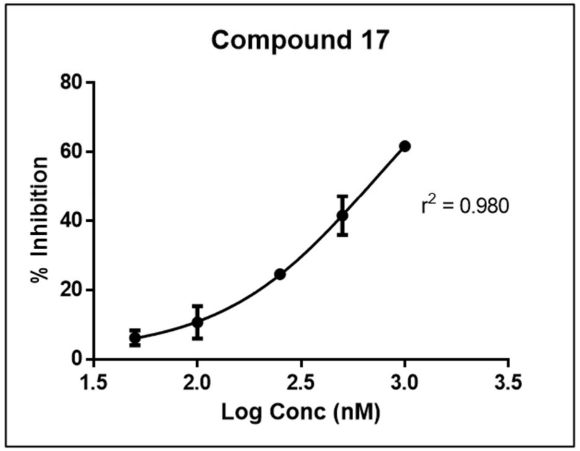

5. Uncropped western blot figures

12

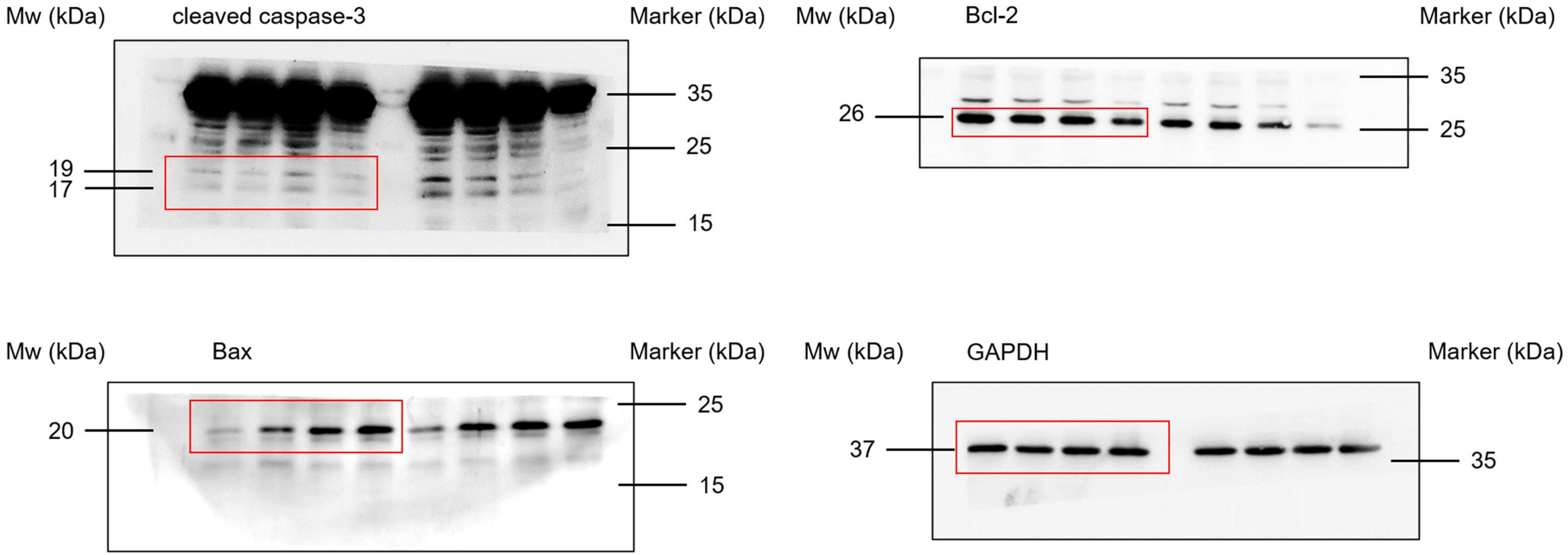

17

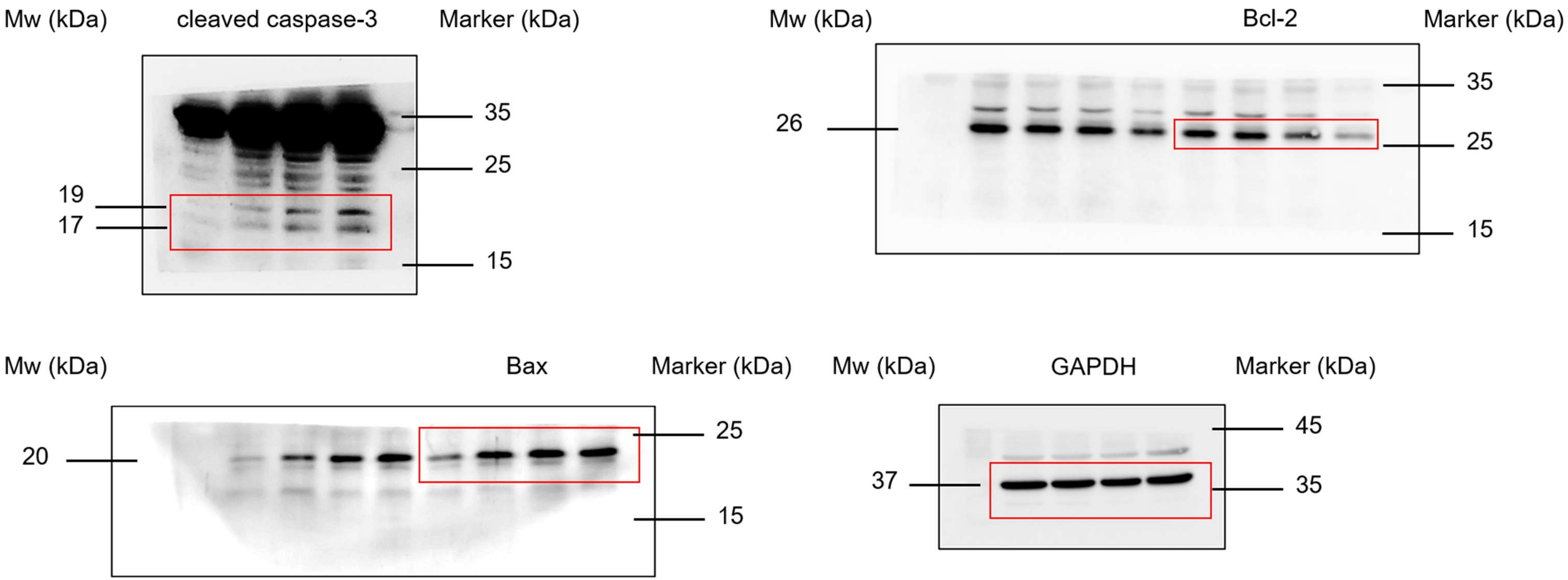
Mw (kDa)
cleaved caspase-3
Marker (kDa)
35
25
19
17
15
Mw (kDa)
Bcl-2
Marker (kDa)
35
26
25
15
Mw (kDa)
Bax
Marker (kDa)
25
20
15
Mw (kDa)
GAPDH
Marker (kDa)
45
37
35
